# Cardiovascular risk–prediction models in cancer survivors: a meta-analysis

**DOI:** 10.1093/eurheartj/ehag562

**Published:** 2026-07-28

**Authors:** Sina Kazemian, Azin Vakilpour, Marielle Scherrer-Crosbie

**Affiliations:** Tehran Heart Center, Cardiovascular Diseases Research Institute, Tehran University of Medical Sciences, Tehran, Iran; Division of Cardiovascular Medicine, Department of Medicine, Hospital of the University of Pennsylvania, 3400 Spruce Street, Philadelphia, PA 19104, USA; Division of Cardiovascular Medicine, Department of Medicine, Hospital of the University of Pennsylvania, 3400 Spruce Street, Philadelphia, PA 19104, USA

**Keywords:** Cancer survivors, Cardio-oncology, Cardiovascular risk score, Cancer therapy-related cardiac dysfunction, Risk prediction model

## Abstract

This systematic review and meta-analysis included 34 studies encompassing 983 438 adult cancer survivors and evaluated the performance of both general-population and cancer-specific cardiovascular risk–prediction models across vascular events, cancer therapy-related cardiac dysfunction or heart failure, arrhythmias, and composite cardiovascular event outcomes. In total, 27 unique risk scores were assessed.

Approximately one-third of model–outcome evaluations demonstrated acceptable-to-good discrimination, defined as an area under the curve of ≥0.70. Among general-population models, four risk scores demonstrated acceptable-to-good discrimination for vascular events, three models were acceptable for CTRCD/heart failure, and two models for arrhythmias. The New Zealand CVD score was the only composite outcome model with an AUC ≥0.70. Cancer-specific scores, such as HFA-ICOS and the Ezaz score, showed high specificity for selected outcomes and may support rule-in decisions, although their lower sensitivity limits use for ruling out risk of CTRCD/heart failure. In our subgroup analysis, model performance varied by cancer type, with more consistent discrimination in breast cancer cohorts and substantially poorer performance in survivors of haematologic malignancies, indicating limited transportability across cancer populations.

In summary, these findings provide a comprehensive overview of available cardiovascular risk–prediction tools and may assist clinicians in selecting appropriate models for primary cardiovascular prevention in cancer survivors while highlighting the need for exposure-aware, externally validated models tailored to specific cancer populations.

## Structured Graphical Abstract

**Structured Graphical Abstract:**
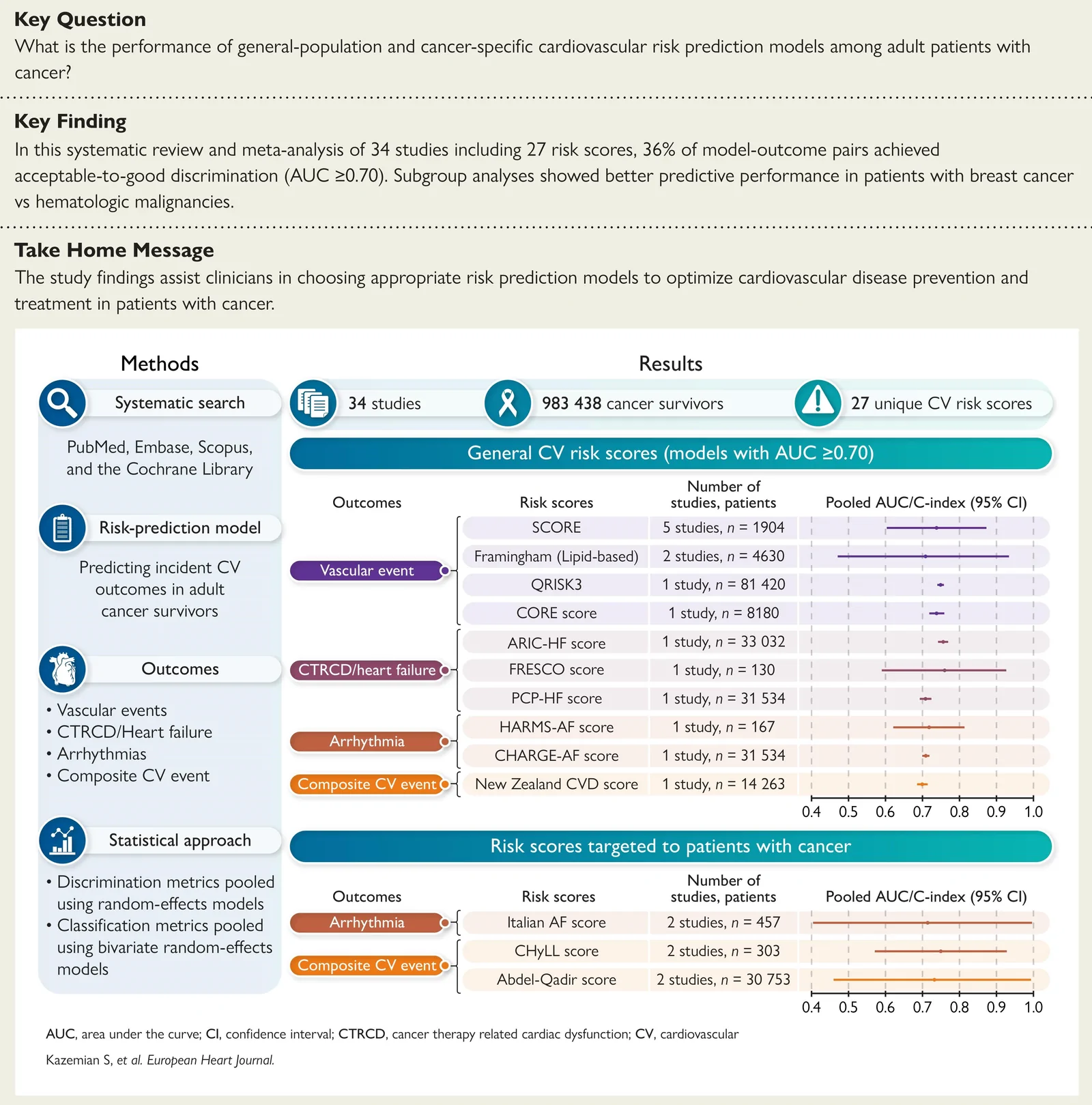

## Introduction

Advances in cancer screening, systemic therapy, and supportive care over recent decades have markedly improved survival across many malignancies, with more than 22 million survivors anticipated in 2035.^1^ Cardiovascular disease (CVD) is one of the leading causes of morbidity and mortality in cancer survivors, highlighting the need for more robust and accurate strategies to identify survivors at elevated risk of CVD.^2^ Current evidence shows that cancer survivors are at higher risk of CVD than individuals without cancer.^3^ The magnitude of this increased CVD risk is heterogeneous and reflects both conventional cardiovascular (CV) risk factors and the cardiotoxic effects of cancer-related treatments.^4^

Current guidelines from the 2022 European Society of Cardiology (ESC) and the American Society of Clinical Oncology (ASCO) recommend baseline CV risk stratification and risk-adapted surveillance before, during, and after potentially cardiotoxic therapy.^5,6^ General-population risk–prediction models were derived in cohorts without cancer and do not fully capture cancer-specific exposures.^7^ External evaluations of these risk scores in cancer survivor cohorts have shown heterogeneous results, with modest-to-good discrimination but frequent underestimation of absolute CVD risk.^7–10^ In response, multiple cancer-specific scores have been developed, which include treatment and disease variables.^5,11–14^ However, many of these models were developed in highly selective populations with limited sample sizes and few rigorous external validations, which constrains their generalizability across diverse cancer survivor populations.^13,14^

To address these gaps, this systematic review and meta-analysis aims to summarize all available evidence and evaluate the predictive performance of CVD risk models in adult cancer survivors. We assessed discrimination and classification performance across both general risk scores and risk scores targeted to patients with cancer for vascular events, cancer therapy-related cardiac dysfunction (CTRCD)/heart failure, arrhythmias, and composite CV event outcomes. Our objectives were to compare the predictive performance of existing risk scores, identify areas where performance is adequate or missing, and highlight methodological priorities for future model development, validation, and clinical use in cardio-oncology practice.

## Methods

This systematic review and meta-analysis was reported in accordance with the Preferred Reporting Items for Systematic Reviews and Meta-Analyses (PRISMA) 2020 statement.^15^ Since all included studies had prior approval from their respective institutional review boards or ethics committees, this study was exempted from additional ethical clearance. The study protocol was previously registered and published with the PROSPERO: International prospective register of systematic reviews database (registration ID: CRD420251054472).^16^

### Search strategy and eligibility criteria

A systematic search of PubMed, Embase, Scopus, and the Cochrane Library databases was performed from inception until 16 July 2025, to identify studies that developed or externally validated risk–prediction models for CV events in cancer survivors. The search strategy combined keywords related to [cancer survivors] AND [cardiovascular events] AND [risk–prediction models], along with their equivalent terms. Full search strategies and terms used for each database are provided in the Supplementary Material.

We included original studies of adults (≥18 years) with a history of cancer that externally validated existing multivariable models or developed and externally validated new multivariable models for predicting incident CV outcomes in survivors. We excluded non-original reports, studies without external validation, those assessing non-CV outcomes or genetic-only scores, and studies with unclear outcome definitions. Detailed eligibility criteria are provided in the Supplementary Material.

### Data extraction and outcomes

After the removal of duplicates, all retrieved records underwent title and abstract screening to identify studies focused on the development or external validation of CV risk–prediction models in cancer survivors. Further details on the screening process and data extraction are described in the Supplementary Material.

Cardiovascular disease outcomes were classified into four predefined groups: (i) vascular events (myocardial infarction, stroke, and peripheral artery disease); (ii) CTRCD/heart failure, defined by a decline in left ventricular ejection fraction and/or the presence of heart failure symptoms;^5^ (iii) arrhythmias (new-onset atrial fibrillation, atrial flutter, and malignant ventricular arrhythmias); and (iv) composite CV events (a combined outcome including any event listed in the previous categories). Risk–prediction model performance was evaluated using discrimination metrics, including the area under the receiver operating characteristic curve (AUC), C-statistic, or C-index, and threshold-based classification metrics, including sensitivity, specificity, and accuracy, within each CV outcome category.

### Quality assessment

The risk of bias in the included studies was evaluated using the prediction model risk of bias assessment tool (PROBAST), designed to evaluate the methodological quality of studies that develop or validate multivariable prediction models and evaluate the risk of bias across four domains.^17^ Two authors independently applied the PROBAST tool to all included studies, resolving disagreements through discussion and mutual consensus. Studies rated as high risk in ≥1 domain were considered to have an overall high risk of bias. Details about the risk of bias assessment across PROBAST domains are provided in the Supplementary Material.

### Statistical analysis

Numerical characteristics of the included studies, such as sample size, mean age, and follow-up duration, were summarized descriptively. Overall mean age and standard deviation (SD) were calculated from aggregate study-level summary statistics using standard formulas for combining independent groups; the mean was calculated as a sample-size-weighted combined mean, and the corresponding combined SD was derived from study-level sample sizes, means, and SDs. The performance metrics for each risk score were reported within the predefined CV outcome groups (vascular events, CTRCD/heart failure, arrhythmias, and composite outcomes) and presented separately for general-population and cancer-specific models.

For risk scores validated in ≥2 studies, discrimination metrics were synthesized using inverse-variance random-effects meta-analysis, with between-study variance estimated using the Sidik–Jonkman estimator. Random-effects 95% confidence intervals (CIs) were calculated using the Hartung–Knapp adjustment to account for the small number of studies included in each meta-analysis. Discrimination metrics reported as AUC, C-statistic, or C-index were considered equivalent and pooled together. Area under the receiver operating characteristic curve values were classified using the following thresholds: 0.50–0.69 (poor), 0.70–0.79 (acceptable/good), 0.80–0.89 (very good), and ≥0.90 (excellent). When only 95% CIs were reported, standard errors (SEs) were calculated by dividing the width of the 95% CI by 3.92. If neither the 95% CI nor SE was available, SEs were imputed from studies with comparable sample sizes and event counts. For risk scores that reported confusion matrix values (true positives, false positives, false negatives, and true negatives) at similar cut-off points, we applied a bivariate random-effects model to synthesize pooled sensitivity, specificity, and accuracy and generate summary receiver operating characteristic curves.^18^ Risk scores evaluated at noncomparable cut-off points across studies were not pooled and were summarized descriptively. Heterogeneity was assessed using Higgins and Thompson’s *I^2^* statistic and the between-study variance (*τ^2^*).

Subgroup analyses were conducted to assess pooled model performances (both discrimination and classification metrics) specifically in patients with breast cancer and haematologic malignancies, analysed separately. Sensitivity analyses were performed by (i) limiting the dataset to prospective studies to assess the impact of study design on risk score performance; (ii) comparing pooled AUCs between studies with >1-year and ≤1-year follow-up durations using a Z-test for independent AUC estimates, with SEs derived from the corresponding 95% CIs; and (iii) excluding studies for which SEs had been imputed to evaluate the robustness of pooled AUC estimates. Meta-regression analysis was conducted to assess the effect of median follow-up duration and population mean age on the discrimination performance (AUC/C-index) of CV risk–prediction models, analysed separately within each outcome group. The results were visualized using bubble plots. All analyses were conducted using R version 4.2.1 (The R Foundation, Vienna, Austria), using the *meta*, *mada*, and *ggplot2* packages. A *P*-value of <.05, based on a two-sided *t*-test, was considered statistically significant.

## Results

**Figure 1:**
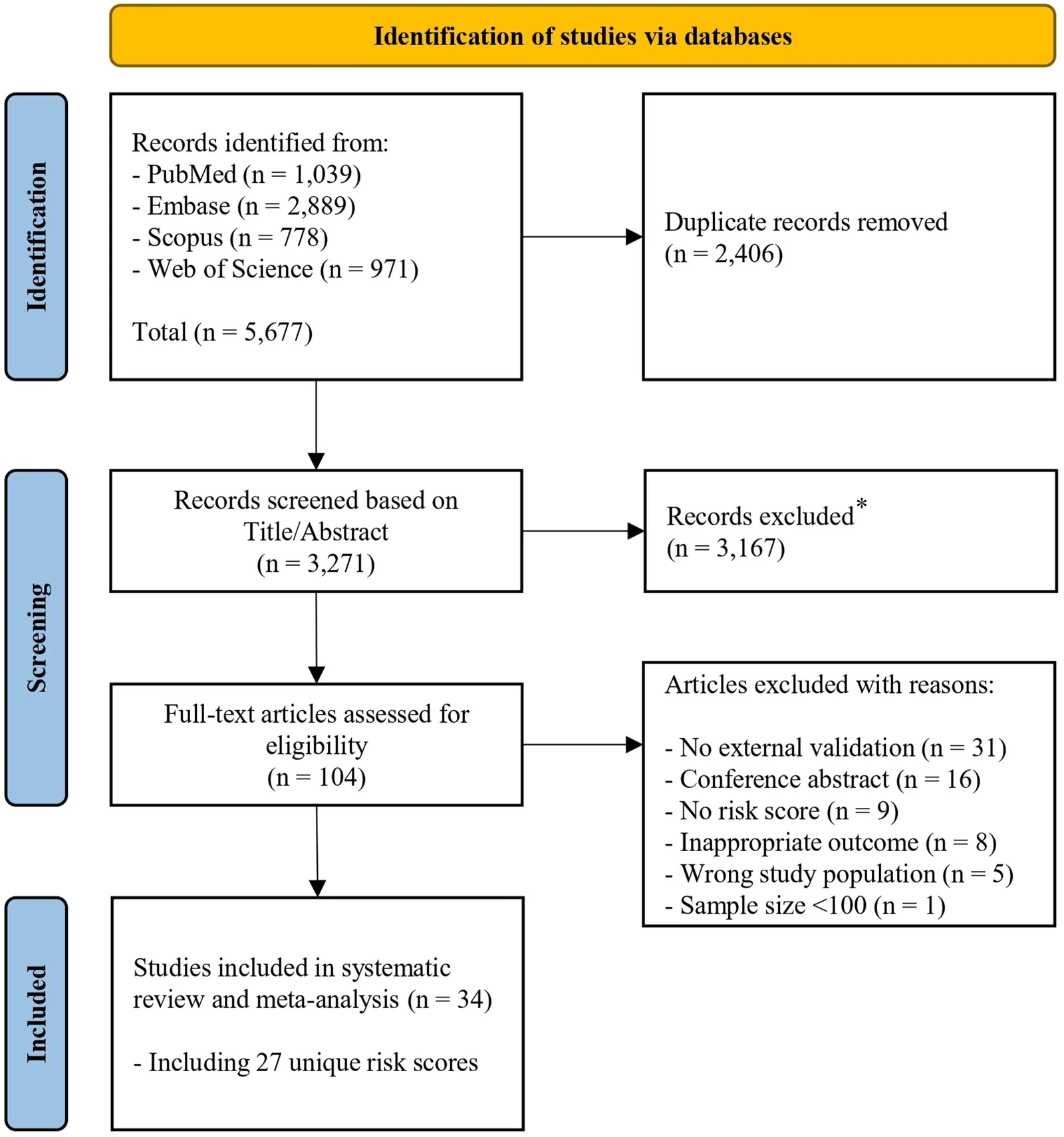
PRISMA flow diagram. *The search strategy was intentionally broad to ensure comprehensive capture. Most title/abstract exclusions reflected records that were not multivariable cardiovascular disease risk–prediction studies with external validation in adult cancer survivors (e.g. descriptive or toxicity reports, non-cardiovascular outcomes, genetic-only models without clinical predictors, or non-original publications)

The systematic search yielded 5677 records. After excluding 2406 duplicates, 3271 records were screened by title/abstract, and 104 full-text articles were assessed for eligibility. Ultimately, 34 studies evaluating 27 unique CV risk–prediction models were included in this systematic review and meta-analysis (*Figure 1*).^7–14,19–44^

### Characteristics of included studies

The included studies were published between 2012 and 2025, with approximately two-thirds published after 2021. Most studies were conducted in Europe (18 studies) and the USA (10 studies), followed by Canada (4 studies) and Taiwan and New Zealand (1 study each). Regarding study design, 27 studies (79%) were retrospective, and 7 studies (21%) were prospective. Key characteristics of the included studies are summarized in *Table 1*.

**Table 1:** Study characteristics of included studies

Study, year	Country	Study design	Number of participants	Median follow-up duration, month	Cancer type	Reported prediction model	Cardiovascular outcome
Ali *et al*., 2025^19^	USA	Retrospective	496	51.0	Breast cancer	HFA-ICOS	CTRCD + HF
Astarita *et al*., 2025^20^	Italy	Prospective	169	11.2	Haematologic malignancies	HFA-ICOS	Composite outcome
Rivero-Santana *et al*., 2025^21^	Spain	Prospective	1066	54.8	Breast cancer (64%), haematologic malignancies (22.3%)	HFA-ICOS	CTRCD + HF
Madaudo *et al*., 2025^22^	Italy	Retrospective	148	31.9	Haematologic malignancies	HFA-ICOS	Vascular event
Jacobson *et al*., 2025^23^	USA	Retrospective	167	64.6	Haematologic malignancies	C2HEST Score CHA_2_DS_2_-VASc HARMS2-AF Score Italian AF Score Mayo CLL AF Score	Arrhythmia
Abiodun *et al*., 2024^8^	UK	Retrospective	1898	24.5	Colorectal (48.6%), breast cancer (21.8%), oesophageal (7.9%), others (21.7%)	QRISK3	Composite outcome
McCracken *et al*., 2024^7^	UK	Retrospective	31 534	163.2	Breast cancer (32.2%), haematologic malignancies (12.0%), prostate (9.0%), brain/CNS (0.8%), lung (0.8%)	CHA_2_DS_2_-VASc CHARGE-AF Framingham (BMI-based) Framingham (lipid-based) PCP-HF Score QRISK3 Qstroke Score SCORE2/SCORE2-OP	Vascular event Arrhythmia CTRCD + HF Composite outcome
Soh and Marwick, 2024^24^	UK	Retrospective	33 032	93.1	Colorectal (48.6%), breast cancer (21.8%), oesophageal (7.9%), others (21.7%)	ARIC-HF Score HFA-ICOS	CTRCD + HF
Di Lisi *et al*., 2024^25^	Italy	Prospective	109	12.0	Breast cancer (32.2%), haematologic (12.0%), prostate (9.0%), brain/CNS (0.8%), lung (0.8%)	HFA-ICOS	CTRCD + HF
Polter *et al*., 2024^9^	USA	Prospective	1244	79.3	Breast cancer (29.4%), prostate (21.5%), colorectal (10.0%), others (39.1%)	10-y ASCVD/PCE	Vascular event
Fernando *et al*., 2024^26^	UK	Retrospective	229	62.9	Haematologic malignancies	HFA-ICOS	Vascular event Composite outcome
Tawfiq *et al*., 2023^27^	New Zealand	Retrospective	14 263	68.4	Breast cancer (27.1%), melanoma (18.6%), prostate (16.1%), colorectal (8.6%)	New Zealand CVD Score	Composite outcome
Cronin *et al*., 2023^28^	Ireland	Retrospective	507	Reported 5-year risk	Breast cancer	HFA-ICOS	CTRCD + HF
Tjong *et al*., 2023^11^	USA	Retrospective	Internal validation: 201 External validation: 102	23.5	Non-small cell lung cancer	CHyLL Score	Composite outcome
Mulas *et al*., 2024^10^	Italy	Retrospective	380	72.0	Haematologic malignancies	SCORE SCORE2/SCORE2-OP	Vascular event
Doukas *et al*., 2023^29^	USA	Retrospective	193	9.0	Haematologic malignancies	HFA-ICOS	CTRCD + HF
Mery *et al*., 2022^30^	France	Retrospective	943	Reported 10-year risk	Breast cancer	Abdel–Qadir score	Composite outcome
Strongman *et al*., 2022^31^	UK	Retrospective	81 420	62.4	Solid cancer (91.0%), haematologic malignancies (9.0%)	QRISK3	Vascular event
Jacobs *et al*., 2022^32^	Belgium	Prospective	324	14.8	Breast cancer (33%), haematologic malignancies (24%), gynaecological (5%)	ASCO Score Mayo Score	CTRCD + HF
Suntheralingam *et al*., 2022^33^	Canada	Retrospective	629	18.0	Breast cancer	Ezaz Score HFA-ICOS NSABP-31 Score	CTRCD + HF
Leoce *et al*., 2021^34^	USA	Retrospective	20 462	90.0	Breast cancer	CORE Score Framingham (lipid-based) SCORE SCORE2/SCORE2-OP	Vascular event
Battisti *et al*., 2021^35^	UK	Retrospective	931	5.3^a^	Breast cancer	HFA-ICOS	CTRCD + HF
Archibald *et al*., 2021^36^	USA	Retrospective	290	Reported 2-year risk	Haematologic malignancies	Framingham (BMI-based) Italian AF Score Mayo CLL AF Score	Arrhythmia
Caocci *et al*., 2019^37^	Italy	Retrospective	85	75.6	Haematologic malignancies	SCORE	Vascular event
Abdel-Qadir *et al*., 2019^12^	Canada	Retrospective	29 810	70.8	Breast cancer	Abdel–Qadir score	Composite outcome
Ferraro *et al*., 2019^38^	Spain	Prospective	130	81.3	Haematologic malignancies	FRESCO Score	CTRCD + HF
Moey *et al*., 2019^39^	USA	Retrospective	127	15	Breast cancer	Ezaz Score	CTRCD + HF
Rushton *et al*., 2017^40^	Canada	Retrospective	143	Reported 3-year risk	Breast cancer	Ezaz Score	CTRCD + HF
Law *et al*., 2017^41^	Canada	Retrospective	152	40.7	Breast cancer	Framingham (lipid-based)	Vascular event CTRCD + HF Arrhythmia Composite outcome
Hu *et al*., 2017^42^	Taiwan	Retrospective	760 339	36.2	Colon (13.8%), hepatoma (13.3%), breast cancer (11.4%), lung (11.2%), head and neck (10.5%), others (35.1%)	CHA_2_DS_2_-VASc CHADS_2_ Score HATCH Score	Arrhythmia
Rea *et al*., 2015^43^	France	Retrospective	57	47.0	Haematologic malignancies	SCORE	Vascular event
Breccia *et al*., 2015^44^	Italy	Retrospective	82	48.0	Haematologic malignancies	SCORE	Vascular event
Ezaz *et al*., 2014^13^	USA	Retrospective	832	Reported 3-year risk	Breast cancer	Ezaz Score	CTRCD + HF
Romond *et al*., 2012^14^	USA	Prospective	944	87.0	Breast cancer	NSABP-31 Score	CTRCD + HF

10-y ASCVD/PCE, 10-year atherosclerotic cardiovascular disease risk using pooled cohort equations; CNS, central nervous system; CTRCD, cancer therapy-related cardiac dysfunction; CVD, cardiovascular disease; HF, heart failure; HFA-ICOS, Heart Failure Association–International Cardio-Oncology Society; QRISK3, cardiovascular risk–prediction algorithm (version 3); USA, United States of America.

^a^In the study by Battisti *et al*., left ventricular ejection fraction was assessed by MUGA scan or echocardiography at baseline and at 16 and 23 weeks for taxane-only regimens and before and after anthracycline use for sequential regimens.

A total of 983 438 cancer survivors were included, with a mean age of 61.2 ± 14.3 years (range: 49.0–73.6 years); 69.8% were female. When stratified by outcomes, mean age was broadly similar across cohorts, ranging from 59.2 ± 8.8 years in studies predicting CTRCD/heart failure to 61.5 ± 11.4 years in studies predicting vascular event outcomes (see Supplementary data online, *Table S1*). Female representation varied substantially between outcomes, largely reflecting that some cohorts exclusively included patients with breast cancer. All studies included patients with a history of cancer according to predefined eligibility criteria, although some targeted specific cancer types. Specifically, 12 studies included only patients with breast cancer,^12–14,19,28,30,33–35,39–41^ 11 studies focused on haematologic malignancies,^10,20,22,23,26,29,36–38,43,44^ and 11 studies included mixed cancer types.^7–9,11,21,24,25,27,31,32,42^ Two studies reported outcomes separately for each cancer type.^7,27^ Details of demographic characteristics, follow-up durations, and eligibility criteria for each study are provided in Supplementary data online, *Table S2*.

### Cardiovascular risk–prediction models and predictors

We identified 27 unique CV risk–prediction models (18 developed for the general population and 9 specifically targeted to patients with cancer). These models incorporated a wide range of predictive factors, including demographics, comorbidities, anticancer treatments, radiation dose, serum biomarkers, and echocardiographic parameters. Across models, several predictors recurred consistently, highlighting their importance in CV risk estimation. The most commonly used variables were age (26 risk scores), systolic blood pressure or history of hypertension (25 risk scores), history of diabetes mellitus (19 risk scores), and smoking status (16 risk scores). The complete list of risk scores and their corresponding predictive factors is provided in Supplementary data online, *Table S3*.

The included studies evaluated various CV outcome groups: CTRCD/heart failure (16 studies), followed by vascular events (11 studies), composite CV events (9 studies), and arrhythmias (5 studies). Definitions of each outcome across studies are summarized in Supplementary data online, *Table S4*.

### Risk prediction in cancer survivors (any cancer)

**Figure 2:**
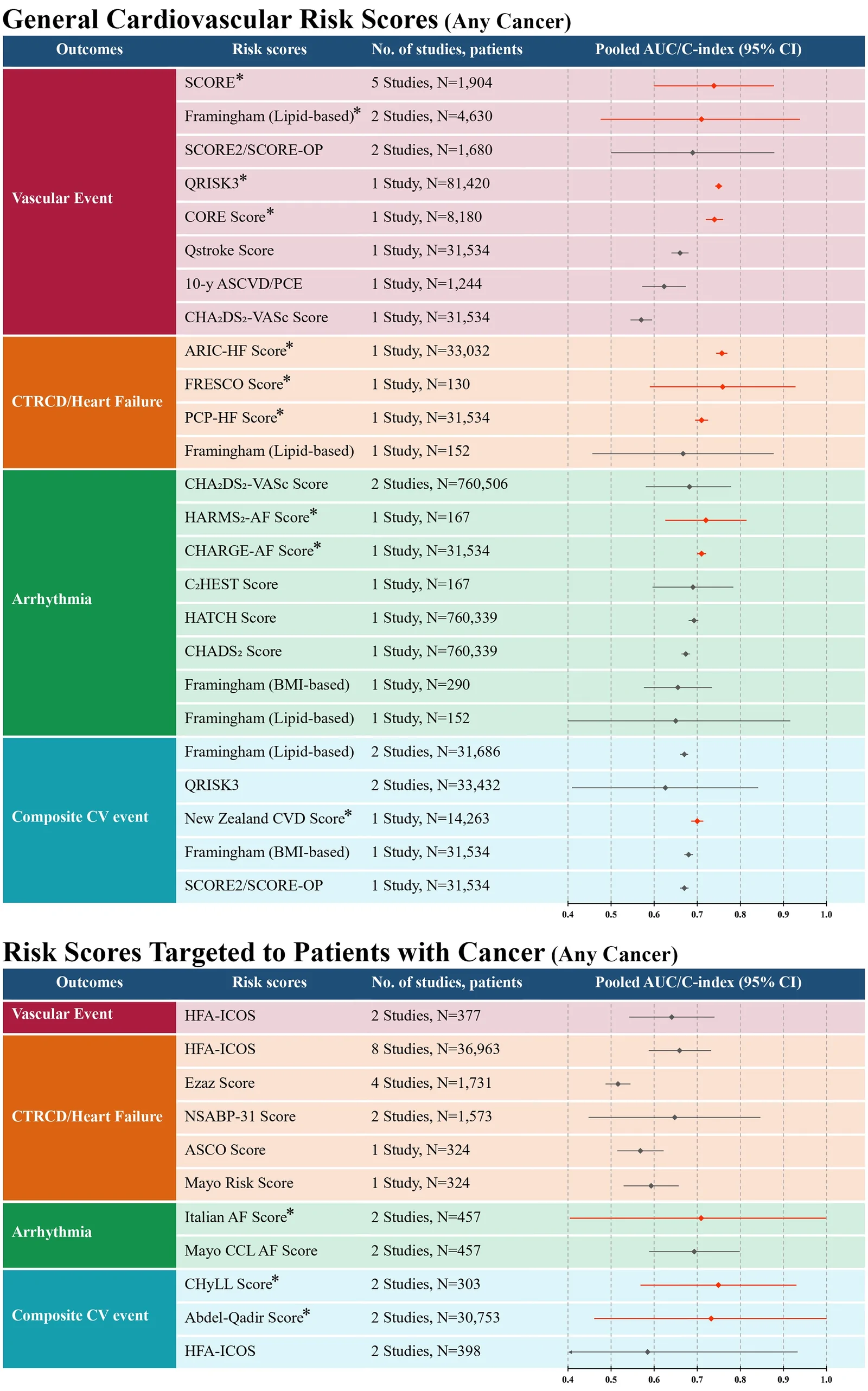
Performance of cardiovascular risk prediction models in cancer survivors. This figure summarizes the discrimination performance (AUC/C-index with 95% CI) of general and cancer-specific cardiovascular risk scores applied to survivors of any cancer type. Estimates were derived from random-effects meta-analysis when ≥2 studies were available; otherwise, single-study results are shown. Risk scores with AUC/C-index values ≥0.70 are marked with an asterisk (*)

Across all reported model evaluations for different CV outcomes, 15 models were validated in ≥2 studies, and 12 were evaluated in only a single study. Among general CV risk scores applied in cancer survivors, four models [SCORE, Framingham (lipid-based), QRISK3, and CORE] demonstrated acceptable/good discrimination (AUC ≥0.70) for predicting vascular events (*Figure 2*). Three risk scores (ARIC-HF, FRESCO, and PCP-HF) showed acceptable/good discrimination for CTRCD/heart failure, while two models (HARMS_2_-AF and CHARGE-AF) achieved AUCs above 0.70 for arrhythmia prediction. For composite CV events, only the New Zealand CVD score met this threshold with an AUC of 0.70 (95% CI 0.69–0.71). Summary ROC curves with summary points for each model are shown in Supplementary data online, *Figure S1*.

Classification metrics and performance (sensitivity, specificity, and accuracy) showed considerable heterogeneity across risk scores (*Table 2*). For vascular event prediction, SCORE ≥5% reported in four studies demonstrated low pooled sensitivity (54.2%) but the highest pooled specificity (81.9%) and moderate pooled accuracy (77.6%), with an *I^2^* of 32.1%. QRISK3 ≥10% reported in two studies achieved the highest pooled sensitivity (84.3%) but low pooled specificity (47.9%) and pooled accuracy (51.1%) with an *I^2^* of 0.1%, whereas Qstroke offered a more balanced profile. For CTRCD/heart failure, PCP-HF had the highest sensitivity (73.0%) and modest specificity (64.8%). For arrhythmia prediction, models showed distinct trade-offs with CHA_2_DS_2_-VASc ≥2 provided the highest sensitivity (79.3%) but low specificity (50.4%), whereas HATCH ≥3 had the highest specificity (87.2%) and accuracy (86.4%) but with very low sensitivity (30.9%). For composite CV events, models generally displayed moderate balance, with Framingham (lipid-based) ≥20% and Framingham (body mass index-based) ≥20% models achieving the highest specificity and sensitivity, respectively.

**Table 2:** Study characteristics of included studies

Outcomes	Risk scores	No. of studies, patients	Sensitivity	Specificity	Accuracy
General cardiovascular risk scores					
Vascular event	SCORE ≥5%	4 studies, *N* = 604	54.2%	81.9%	77.6%
	Framingham (lipid-based) ≥20%	1 study, *N* = 152	50.0%	69.3%	69.1%
	SCORE2/SCORE2-OP ≥7.5%	1 study, *N* = 380	60.6%	50.7%	51.6%
	QRISK3 ≥10%	1 study, *N* = 81 420	84.3%	47.9%	51.1%
	Qstroke Score (high vs low risk)	1 study, *N* = 31 534	67.0%	61.9%	62.0%
	CHA_2_DS_2_-VASc Score ≥2	1 study, *N* = 31 534	52.0%	56.1%	56.0%
CTRCD/Heart Failure	PCP-HF Score (high vs low risk)	1 study, *N* = 31 534	73.0%	64.8%	65.0%
	Framingham (lipid-based) ≥20%	1 study, *N* = 152	62.5%	70.8%	70.4%
Arrhythmia	CHA_2_DS_2_-VASc Score ≥2	1 study, *N* = 760 339	79.3%	50.4%	50.8%
	CHARGE-AF Score (high vs low risk)	1 study, *N* = 31 534	69.0%	64.8%	65.0%
	HATCH Score ≥3	1 study, *N* = 760 339	30.9%	87.2%	86.4%
	CHADS_2_ Score ≥2	1 study, *N* = 760 339	51.7%	73.1%	72.8%
	Framingham (BMI-based) ≥20%	1 study, *N* = 290	33.3%	85.4%	76.2%
	Framingham (lipid-based) ≥20%	1 study, *N* = 152	60.0%	70.1%	69.7%
Composite CV event	Framingham (lipid-based) ≥20%	2 studies, *N* = 31 686	63.0%	67.3%	62.1%
	QRISK3 ≥10%	2 studies, *N* = 33 432	61.7%	55.9%	56.2%
	Framingham (BMI-based) ≥20%	1 study, *N* = 31 534	67.0%	62.5%	63.0%
	SCORE2/SCORE2-OP ≥7.5%	1 study, *N* = 31 534	66.0%	62.6%	63.0%
Risk scores targeted to patients with cancer					
Vascular event	HFA-ICOS (very-high/high vs medium/low risk)	2 studies, *N* = 377	76.4%	65.6%	69.2%
CTRCD/Heart Failure	HFA-ICOS (very-high/high vs medium/low risk)	7 studies, *N* = 3931	19.3%	94.2%	81.7%
	Ezaz Score ≥4	4 studies, *N* = 1731	12.2%	88.0%	69.8%
	NSABP-31 Score ≥65	1 study, *N* = 629	40.4%	70.5%	63.3%
	ASCO Score (high vs low risk)	1 study, *N* = 324	64.3%	52.0%	56.8%
	Mayo Risk Score (high vs low risk)	1 study, *N* = 324	81.0%	37.9%	54.6%
Arrhythmia	Italian AF Score ≥5	1 study, *N* = 290	35.3%	92.1%	82.1%
	Mayo CCL AF Score ≥4	1 study, *N* = 290	64.7%	66.1%	65.9%
Composite CV event	CHyLL Score ≥5	2 studies, *N* = 303	90.0%	34.4%	39.9%
	HFA-ICOS (very-high/high vs medium/low risk)	2 studies, *N* = 398	53.3%	65.7%	62.7%

AF, atrial fibrillation; BMI, body mass index; CHARGE-AF, Cohorts for Heart and Aging Research in Genomic Epidemiology-Atrial Fibrillation; CTRCD, cancer therapy-related cardiac dysfunction; CV, cardiovascular; HF, heart failure; HFA-ICOS, Heart Failure Association–International Cardio-Oncology Society; NSABP-31, National Surgical Adjuvant Breast and Bowel Project trial 31; PCP-HF, Pooled Cohort Equations to Prevent Heart Failure; SCORE2/SCORE2-OP, Systematic COronary Risk Evaluation 2/SCORE2-Older Persons.

For scores developed in patients with cancer, no model demonstrated acceptable/good discrimination for vascular event or CTRCD/heart failure prediction. The Italian AF score was the only model with good discrimination for arrhythmia, and two risk scores (CHyLL and Abdel–Qadir scores) showed good discrimination for composite CV event prediction (*Figure 2*). For vascular events, the Heart Failure Association–International Cardio-Oncology Society (HFA-ICOS; very-high/high vs medium/low risk; reported in two studies) showed 76.4% pooled sensitivity and 65.6% pooled specificity (*I^2^*: 0%). For CTRCD/heart failure, the Mayo risk score achieved the highest sensitivity (81.0%), while HFA-ICOS (seven studies; pooled specificity: 94.2%; *I^2^*: 24.8%) and Ezaz ≥4 (four studies; pooled specificity: 88.0%; *I^2^*: 0%) achieved the highest specificities, respectively. In arrhythmia prediction, the Italian AF score ≥5 offered high specificity (92.1%) but low sensitivity (35.3%), whereas the Mayo CCL AF score provided more balanced values. For composite CV events, the CHyLL score reported in two studies had the highest pooled sensitivity (90.0%; *I^2^*: 28.0%), while HFA-ICOS reported in two studies demonstrated a more balanced performance with the highest pooled specificity (65.7%) and accuracy (62.7% *I^2^*: 0%) (*Table 2*).

### Breast cancer survivors

**Figure 3:**
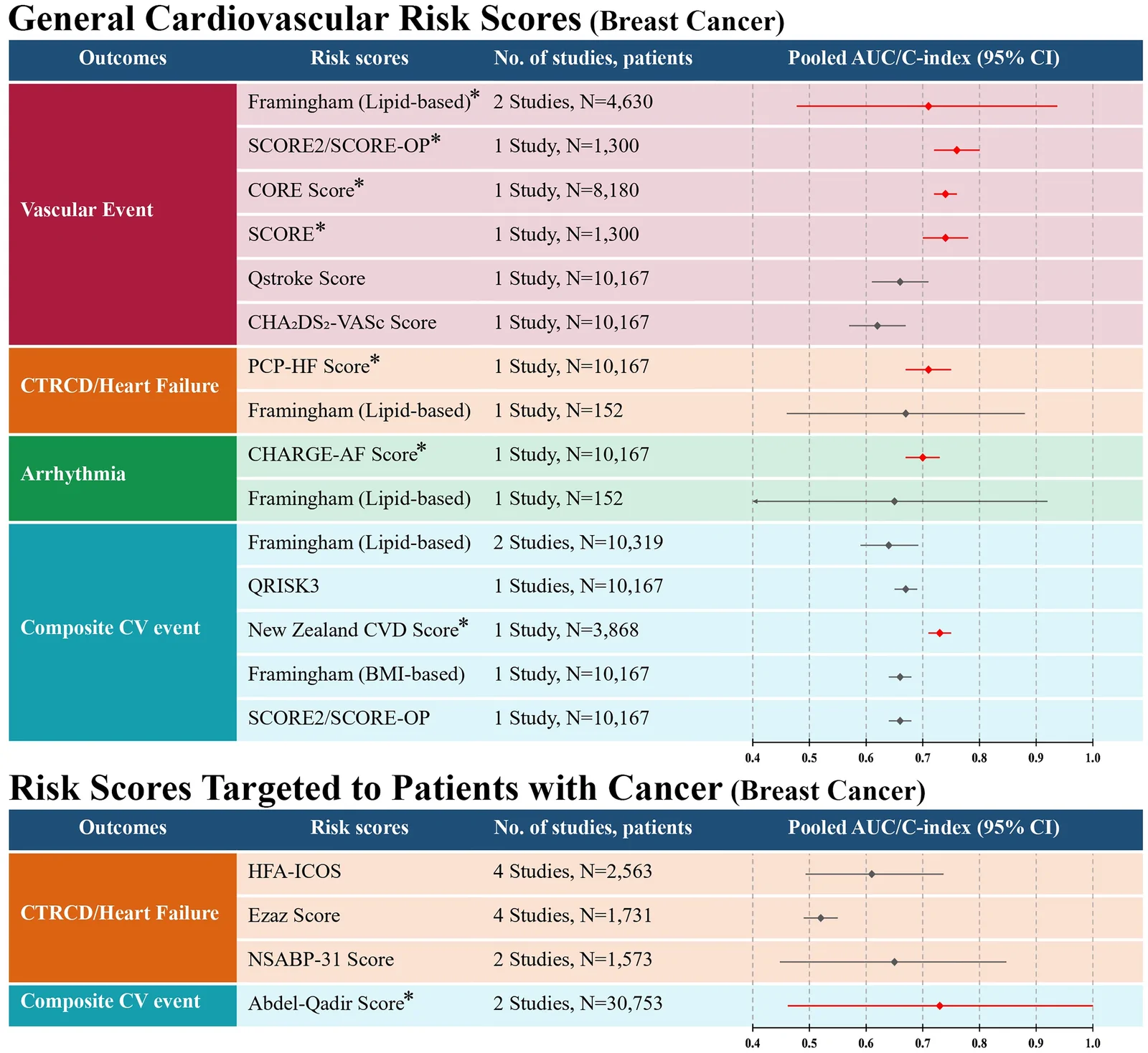
Discrimination of cardiovascular risk scores in breast cancer survivors. This figure summarizes the discrimination performance (AUC/C-index with 95% CI) of general and cancer-specific cardiovascular risk scores applied to only survivors of breast cancer. Estimates were derived from random-effects meta-analysis when ≥2 studies were available; otherwise, single-study results are shown. Risk scores with AUC/C-index values ≥0.70 are marked with an asterisk (*)

In a subgroup analysis of patients with breast cancer, 19 CV risk–prediction models were evaluated and 8 (42%) demonstrated acceptable/good discrimination (AUC ≥0.70).^7,12–14,19,27,28,30,33–35,39–41^ Among general CV risk scores, four models [Framingham (lipid-based), SCORE2/SCORE2-OP, CORE, and SCORE] achieved this threshold for vascular event prediction, while only the PCP-HF score showed good discrimination for CTRCD/heart failure prediction. For arrhythmia and composite CV events, CHARGE-AF [AUC: 0.70 (95% CI 0.67–0.73)] and New Zealand CVD score [AUC: 0.73 (95% CI 0.71–0.75)] were the only risk–prediction models with good discrimination, respectively (*Figure 3*).

Among cancer-specific scores in breast cancer survivors, none demonstrated good discrimination for CTRCD/heart failure prediction. The Abdel–Qadir score was the only model with good discrimination for composite CV event prediction [two studies; pooled AUC: 0.73 (95% CI 0.46–1.00); *I^2^*: 94.9%] (*Figure 3*). We observed high variability in classification performance across risk scores; detailed results for breast cancer cohorts are presented in Supplementary data online, *Table S5*, with summary ROC curves in Supplementary data online, *Figure S2*.

### Haematologic malignancy survivors

**Figure 4:**
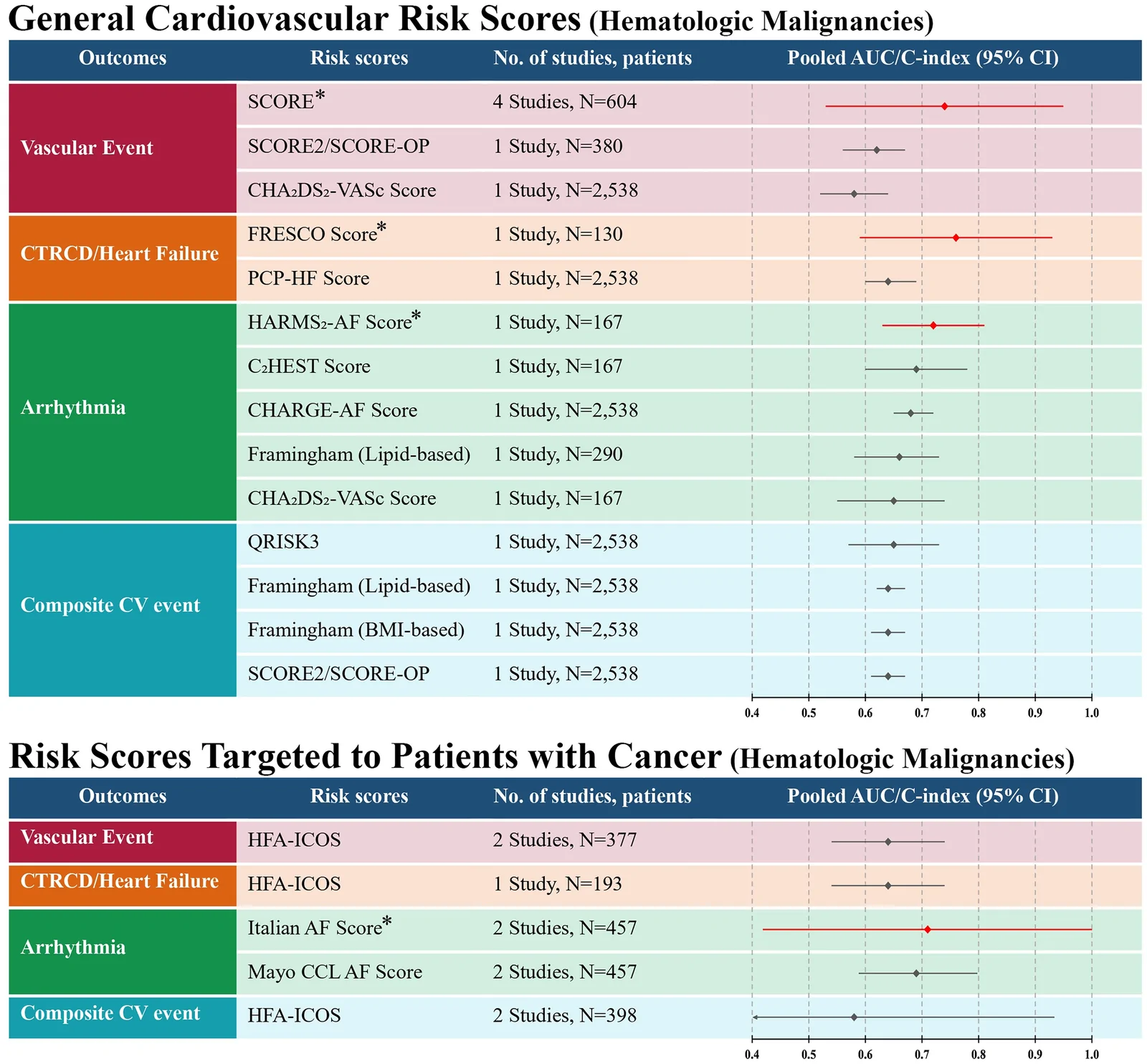
Discrimination of cardiovascular risk scores in haematologic malignancy survivors. This figure summarizes the discrimination performance (AUC/C-index with 95% CI) of general and cancer-specific cardiovascular risk scores applied to only survivors of haematologic malignancies. Estimates were derived from random-effects meta-analysis when ≥2 studies were available; otherwise, single-study results are shown. Risk scores with AUC/C-index values ≥0.70 are marked with an asterisk (*)

In subgroup analysis of patients with haematologic malignancies, 20 CV risk–prediction models were assessed, but only 4 (20%) demonstrated acceptable/good discrimination.^7,10,20,22,23,26,29,36–38,43,44^ Among general CV risk scores, SCORE [four studies; pooled AUC: 0.74 (95% CI 0.53–0.95); *I^2^*: 90.0%] and FRESCO scores [AUC: 0.76 (95% CI 0.59–0.93)] showed good discrimination for the prediction of vascular events and CTRCD/heart failure, respectively. For arrhythmia, only HARMS_2_-AF achieved this threshold (AUC: 0.72; 95% CI 0.63–0.81), whereas no general model had an AUC >0.70 for composite CV events (*Figure 4*).

Among risk scores targeted to patients with haematologic malignancies, none demonstrated good discrimination for predicting vascular events, CTRCD/heart failure, or composite CV events. For arrhythmia, only the Italian AF score [two studies; pooled AUC: 0.71 (95% CI 0.42–1.00); *I^2^*: 0%] showed good discriminative performance (*Figure 4*). Classification metrics demonstrated substantial variability across risk scores; however, sensitivities and specificities were low. Detailed results for haematologic malignancy cohorts are presented in Supplementary data online, *Table S6*, with summary ROC curves in Supplementary data online, *Figure S3*.

### Sensitivity analysis

To investigate the impact of study design on model performance, we restricted the analysis to the seven prospective studies in patients with any cancer type.^9,14,20,21,25,32,38^ Of the seven risk scores assessed, three demonstrated acceptable/good discrimination (AUC ≥0.70; Supplementary data online, *Figure S4*). For CTRCD/heart failure, the HFA-ICOS and NSABP-31 scores showed small, non-significant improvements in discrimination compared with the overall analysis including all studies (ΔAUC: +0.04 and +0.05, respectively). For composite CV events, the predictive performance of HFA-ICOS exhibited a slight, non-significant decline (ΔAUC: −0.02).

Across the included studies, four (11.8%) studies had a median follow-up duration of ≤1 year. In a further sensitivity analysis, we compared the discriminatory performance of risk scores between studies with ≤1-year and >1-year follow-up durations. For CTRCD/heart failure, the HFA-ICOS score demonstrated significantly better discrimination in studies with >1-year follow-up compared with those with ≤1-year follow-up [pooled AUC: 0.70 (95% CI 0.59–0.80); *I^2^*: 93.8% vs 0.59 (95% CI 0.45–0.72); *I^2^*: 51.0%; *P*: .019]. In contrast, for composite CV events, no significant difference in predictive performance was observed between the two follow-up duration groups (see Supplementary data online, *Table S7*).

Another sensitivity analysis was conducted after excluding AUC estimates from studies for which SEs had been imputed. Overall, 6 of 34 studies (17.6%) included at least one imputed SE for an AUC estimate. Exclusion of these estimates resulted in minimal changes in AUC estimates (ΔAUC range: −0.05 to +0.07), with no statistically significant differences observed (see Supplementary data online, *Table S8*).

### Meta-regression

Meta-regression analyses demonstrated that median follow-up duration had a significant modifying effect on the discrimination performance of CV risk scores. Longer follow-up was associated with higher AUC values for predicting CTRCD/heart failure (estimated regression coefficient ($\hat{\beta }$) per year: 0.017; *P*: .003; residual between-study *I^2^*: 93.1%) and arrhythmia ($\hat{\beta }$: 0.003; *P*: .006; residual between-study *I^2^*: 69.2%). In contrast, the modifying effect of follow-up duration on the discrimination performance for vascular events and composite CV events was small and of uncertain magnitude. Also, meta-regression analyses assessing cohort mean age demonstrated that its modifying effect on the discrimination performance of risk scores was small and of uncertain magnitude across all four cardiovascular outcomes. The corresponding bubble plots for each outcome are illustrated in Supplementary data online, *Figures S5*–*S12*.

### Quality assessment and applicability

The methodological quality of the included studies, evaluated using the PROBAST tool, was heterogeneous. The majority of studies (68%) had a high risk of bias in at least one domain, most commonly due to small sample sizes or retrospective design, and 41% presented concerns regarding applicability. Only 24% of studies were rated as overall low risk of bias, and 50% demonstrated low applicability concerns. Comprehensive domain-level assessments of risk of bias and applicability are reported in the Supplementary Results, and summary graphs are provided in Supplementary data online, *Figures S13* and *S14*.

## Discussion

In this systematic review and meta-analysis of 34 studies evaluating 27 unique CV risk scores in 983 438 cancer survivors, we report the following findings: (i) across all cancer types (36 risk–prediction models), 13 risk scores (36%) achieved an acceptable-to-good predictive value (10 general-population and 3 cancer-specific scores); (ii) among breast cancer survivors (19 risk–prediction models), 8 (42%) met this threshold (7 general-population and 1 cancer-specific scores); (iii) in haematologic malignancy survivors (20 risk–prediction models), 4 (20%) reached this threshold (3 general-population and 1 cancer-specific scores); (iv) risk–prediction models discriminated vascular events more reliably than other CV outcomes, a pattern observed consistently in pooled analyses of any cancer type, breast cancer, and haematologic malignancy survivors. When comparing cancer types, the proportion of models with good discrimination was highest in breast cancer cohorts, followed by pooled analyses of any cancer type and haematologic malignancy cohorts (*Structured Graphical Abstract*).

### Differences in risk–prediction models across cardiovascular outcomes

For vascular events, sensitivities and specificities of the CV risk scores targeted to the general population commonly reached or exceeded 80% thresholds, suggesting that the scores performed better than for other CV outcomes. This finding can be attributed to differences in both the development of the risk–prediction models and in the definition of these CV outcomes. The majority of general-population CV risk calculators were originally developed for atherosclerotic disease and emphasize conventional CV risk factors that were most frequently included across models. Moreover, vascular events are captured through established diagnostic pathways and standardized coding, which reduces misclassification. In contrast, the detection of CTRCD/heart failure and arrhythmias requires protocolized follow-up, by echocardiography and continuous rhythm monitoring, respectively.

All risk scores performed less well in the prediction of CTRCD/heart failure than for vascular events. In particular, sensitivity was comparatively low in cancer-targeted risk scores, so these tools are not effective in ruling out risk and should likely be combined with biomarkers and echocardiographic findings. It is noteworthy that the CTRCD/heart failure risk depends on therapy exposures not included in calculators (such as cumulative anthracycline dose, number of HER2-targeted therapy cycles, and radiation field). A sensitivity analysis restricted to prospective cohorts demonstrated similar patterns, although larger prospective validations with standardized follow-up and explicit threshold reporting are needed to strengthen classification and clinical utility.

### Differences in risk–prediction models across cancer types

To our knowledge, this is the first study to systematically synthesize all available evidence on the performance of CV risk–prediction models across cancer survivor populations and to compare results by cancer type. The proportion of models achieving acceptable/good discrimination (AUC ≥0.70) was highest in breast cancer cohorts (42%) and lowest in haematologic malignancies (20%). These findings indicate that established risk scores transfer more readily to breast cancer survivors than to survivors of haematologic malignancies. Several measurable factors likely explain this difference. Exposure profiles in breast cancer are comparatively homogeneous and better documented (such as cumulative anthracycline dose and radiation fields), and survivorship programmes more often include structured follow-up with systematic capture of CV risk factors and outcomes, which facilitates local recalibration and more reliable ascertainment.^45^ In addition, several breast cancer treatments demonstrated dose–response associations with vascular outcomes, exemplified by the proportional increase in ischaemic heart disease with higher mean heart dose after radiotherapy, which supports the transferability of vascular risk–prediction models once calibration is addressed.^46^

In contrast, haematologic malignancies usually involve heterogeneous multi-agent regimens and targeted therapies, together with recurrent infections and high inflammation, which elevate non-ischaemic risks. Reviews in haematologic cardio-oncology and drug-specific analyses document elevated rates of atrial fibrillation with Bruton tyrosine kinase inhibitors and broader cardiotoxicity profiles across these treatments, reinforcing the need for exposure-aware prediction in this population.^47^ Furthermore, the optimal type, frequency, and duration of imaging surveillance for haematologic malignancy survivors, particularly for those treated with emerging therapies such as immune checkpoint inhibitors, remain understudied.^48^ These gaps of knowledge can limit the consistent ascertainment of cardiotoxic outcomes that require planned follow-up echocardiography or rhythm monitoring.^49^ Ultimately, current evidence supports using general vascular risk–prediction models in breast cancer, while in haematologic malignancies, model estimates should be interpreted cautiously, with lower action thresholds and closer follow-up than score values alone would suggest.

### Clinical implications and future directions

The findings of the present study may assist clinicians in making more informed decisions when selecting risk–prediction models for primary CV prevention in cancer survivors. In practice, the choice of model can be guided by its intended application: risk scores with high sensitivity (such as QRISK3, CHyLL, and Mayo Risk Scores) may be preferable when ruling out risk is required, whereas models with high specificity (such as HFA-ICOS, Ezaz, SCORE, and HATCH scores) are better suited to confirm it. A parallel testing strategy that integrates risk scores with complementary diagnostic characteristics—for instance, combining a model with high sensitivity for initial screening with another with high specificity for confirmation—may improve overall predictive certainty by mitigating the limitations inherent to any single model.^50^ Ultimately, in line with the 2022 ESC cardio-oncology guideline recommendations, clinical decision-making should integrate risk–score estimates with individual patients’ factors, including specific treatment exposures, biomarker profiles, and imaging findings.^5^

We must highlight that our findings should be interpreted in the context of the existing evidence base, which is predominantly from populations treated with anthracyclines, HER2-directed therapies, and less frequently tyrosine kinase inhibitor-based chemotherapy regimens. Cardiovascular disease risk data for patients receiving emerging treatments, such as immune checkpoint inhibitors and other immunotherapy, remain limited.^48^ This underscores a critical need for future validation studies to enable better risk stratification in these populations. In addition, we observed frequent incomplete reporting of key performance metrics, including classification and reclassification indices. Future validation studies should adhere to standardized reporting guidelines to enable a more comprehensive and clinically meaningful assessment of the utility of CV risk–prediction models.^51^

Finally, recent advances in using artificial intelligence (AI) and machine learning algorithms have offered a promising opportunity to improve CV risk prediction in cardio-oncology.^52^ These approaches can help discover novel risk factors by integrating different data types, including clinical variables, genetics, biomarkers, and imaging data, and support the development of high-dimensional, non-linear models capable of identifying complex associations that may not be captured by conventional statistical approaches or single risk scores.^53,54^ Moreover, integrating AI-based risk–prediction tools into electronic health record-driven clinical workflows can enable real-time CV risk stratification and facilitate the identification of patients with cancer who would benefit from intensified CV surveillance.^55,56^

### Limitations

The findings of this study should be interpreted in light of several limitations. First, most included studies were observational and predominantly retrospective, increasing risks of selection bias, information bias, and residual confounding. Second, outcome definitions, surveillance methods, and operating thresholds varied across studies; many cohorts lacked standardized follow-up echocardiography or rhythm monitoring, likely introducing misclassification for CTRCD/heart failure and arrhythmias and influencing pooled classification estimates. Third, stratified meta-analysis by specific anticancer treatments was not feasible because therapeutic regimens were highly heterogeneous (different drug classes, cumulative doses, combinations, sequencing, and conditioning regimens), preventing meaningful aggregation into discrete, comparable categories for pooled analysis. Fourth, several risk–prediction models had limited external validation and were evaluated in only a single study, which limits the generalizability of their reported performance. Fifth, heterogeneity across included cohorts may have influenced pooled estimates of model performance. We explored potential sources of heterogeneity through subgroup analyses by cancer type, sensitivity analyses restricted to prospective studies, and meta-regression by cohort mean age and follow-up duration. These analyses demonstrated that discrimination for risk scores predicting CTRCD/heart failure and arrhythmia outcomes improved with longer follow-up duration, indicating that differences in the timing and length of follow-up across studies may influence comparisons of model performance. Nevertheless, residual heterogeneity remains, and larger, well-designed external validation studies are needed to confirm the robustness and transferability of these models across cancer survivor populations. Sixth, because CV outcomes in several included studies were assessed during or early after cancer treatment, competing risks such as cancer-related mortality may have limited the observation of cardiovascular events, potentially affecting estimates of model performance. These estimates may also have been influenced by survivor bias, as CV outcomes were more likely to be captured among individuals who survived long enough to undergo follow-up assessment. Finally, geographic representation was concentrated in Europe and North America, and some cancer types (e.g. central nervous system and sarcoma) were sparsely represented, potentially constraining the generalizability of findings.

## Conclusion

In this systematic review and meta-analysis of 34 studies evaluating 27 CV risk–prediction models in 983 438 cancer survivors, general-population scores discriminated vascular events more reliably than CTRCD/heart failure, arrhythmias, or composite CV outcomes, while classification characteristics varied across models and thresholds. Cancer-targeted scores such as HFA-ICOS and Ezaz score demonstrated high specificity for CTRCD/heart failure and may assist with rule-in decisions, but their lower sensitivity limits use for ruling out risk. Performance was more consistent in breast cancer cohorts and less consistent in haematologic malignancy survivors, indicating differential transferability by cancer type.

## Disclosure of AI programmes

We utilized AI tools, specifically ChatGPT 5.2 and Grammarly GO, to aid in rephrasing portions of this article. The purpose was to improve clarity and readability only. Nevertheless, every piece of AI-generated content was carefully reviewed and verified by the authors to ensure accuracy and integrity.

## Supplementary Material

ehag562_Supplementary_Data

## Declarations

### Data Availability

The data that support the findings of this study are available from the corresponding author upon reasonable request.

## References

[1] Wagle NS, Nogueira L, Devasia TP, Mariotto AB, Yabroff KR, Islami F, et al Cancer treatment and survivorship statistics, 2025. CA Cancer J Clin 2025;75:308–340. doi: 10.3322/caac.70011 40445120 PMC12223361

[2] Sturgeon KM, Deng L, Bluethmann SM, Zhou S, Trifiletti DM, Jiang C, et al A population-based study of cardiovascular disease mortality risk in US cancer patients. Eur Heart J 2019;40:3889–97. doi: 10.1093/eurheartj/ehz766 31761945 PMC6925383

[3] Li Q, Zhang G, Li X, Xu S, Wang H, Deng J, et al Risk of cardiovascular disease among cancer survivors: systematic review and meta-analysis. EClinicalMedicine 2025;84:. doi: 10.1016/j.eclinm.2025.103274 PMC12169785 40524799

[4] Wilcox NS, Amit U, Reibel JB, Berlin E, Howell K, Ky B. Cardiovascular disease and cancer: shared risk factors and mechanisms. Nat Rev Cardiol 2024;21:617–31. doi: 10.1038/s41569-024-01017-x 38600368 PMC11324377

[5] Lyon AR, López-Fernández T, Couch LS, Asteggiano R, Aznar MC, Bergler-Klein J, et al 2022 ESC guidelines on cardio-oncology developed in collaboration with the European Hematology Association (EHA), the European Society for Therapeutic Radiology and Oncology (ESTRO) and the International Cardio-Oncology Society (IC-OS). Eur Heart J 2022;43:4229–361. doi: 10.1093/eurheartj/ehac244 36017568

[6] Armenian SH, Lacchetti C, Barac A, Carver J, Constine LS, Denduluri N, et al Prevention and monitoring of cardiac dysfunction in survivors of adult cancers: American Society of Clinical Oncology clinical practice guideline. J Clin Oncol 2017;35:893–911. doi: 10.1200/jco.2016.70.5400 27918725

[7] McCracken C, Condurache DG, Szabo L, Elghazaly H, Walter FM, Mead AJ, et al Predictive performance of cardiovascular risk scores in cancer survivors from the UK biobank. JACC CardioOncol 2024;6:575–88. doi: 10.1016/j.jaccao.2024.05.015 39239345 PMC11372025

[8] Abiodun A, Shawe-Taylor M, Tyebally S, Bagkeris E, Bajomo O, Artico J, et al Predicting cardiovascular events with fluoropyrimidine chemotherapy using a standard cardiovascular risk calculator. ESC Heart Fail 2024;11:3041–51. doi: 10.1002/ehf2.14879 38845140 PMC11424348

[9] Polter EJ, Blaes A, Wolfson J, Lutsey PL, Florido R, Joshu CE, et al Performance of the pooled cohort equations in cancer survivors: the atherosclerosis risk in communities study. J Cancer Surviv 2024;18:124–34. doi: 10.1007/s11764-023-01379-0 37140677 PMC11050671

[10] Mulas O, Abruzzese E, Luciano L, Iurlo A, Attolico I, Castagnetti F, et al The new systematic coronary risk evaluation (SCORE2 and SCORE2-OP) estimates the risk of arterial occlusive events in chronic myeloid leukemia patients treated with nilotinib or ponatinib. Ann Hematol 2024;103:427–36. doi: 10.1007/s00277-023-05556-0 38012435 PMC10798925

[11] Tjong MC, Zhang SC, Gasho JO, Silos KD, Gay C, McKenzie EM, et al External validation of cardiac disease, hypertension, and logarithmic left anterior descending coronary artery radiation dose (CHyLL) for predicting major adverse cardiac events after lung cancer radiotherapy. Clin Transl Radiat Oncol 2023;42:100660. doi: 10.1016/j.ctro.2023.100660 37545790 PMC10403724

[12] Abdel-Qadir H, Thavendiranathan P, Austin PC, Lee DS, Amir E, Tu JV, et al Development and validation of a multivariable prediction model for major adverse cardiovascular events after early stage breast cancer: a population-based cohort study. Eur Heart J 2019;40:3913–20. doi: 10.1093/eurheartj/ehz460 31318428

[13] Ezaz G, Long JB, Gross CP, Chen J. Risk prediction model for heart failure and cardiomyopathy after adjuvant trastuzumab therapy for breast cancer. J Am Heart Assoc 2014;3:e000472. doi: 10.1161/jaha.113.000472 24584736 PMC3959671

[14] Romond EH, Jeong JH, Rastogi P, Swain SM, Geyer CE Jr, Ewer MS, et al Seven-year follow-up assessment of cardiac function in NSABP B-31, a randomized trial comparing doxorubicin and cyclophosphamide followed by paclitaxel (ACP) with ACP plus trastuzumab as adjuvant therapy for patients with node-positive, human epidermal growth factor receptor 2-positive breast cancer. J Clin Oncol 2012;30:3792–9. doi: 10.1200/jco.2011.40.0010 22987084 PMC3478574

[15] Page MJ, McKenzie JE, Bossuyt PM, Boutron I, Hoffmann TC, Mulrow CD, et al The PRISMA 2020 statement: an updated guideline for reporting systematic reviews. BMJ 2021;372:n71. doi: 10.1136/bmj.n71 33782057 PMC8005924

[16] Kazemian S, Scherrer-Crosbie M. *Performance of Cardiovascular Risk Prediction Models in Cancer Survivors: A Systematic Review and Meta-analysis. PROSPERO 2025 CRD420251054472*. https://www.crd.york.ac.uk/PROSPERO/view/CRD420251054472 (20 July 2025, date last accessed).

[17] Wolff RF, Moons KGM, Riley RD, Whiting PF, Westwood M, Collins GS, et al PROBAST: a tool to assess the risk of bias and applicability of prediction model studies. Ann Intern Med 2019;170:51–8. doi: 10.7326/m18-1376 30596875

[18] Reitsma JB, Glas AS, Rutjes AW, Scholten RJ, Bossuyt PM, Zwinderman AH. Bivariate analysis of sensitivity and specificity produces informative summary measures in diagnostic reviews. J Clin Epidemiol 2005;58:982–90. doi: 10.1016/j.jclinepi.2005.02.022 16168343

[19] Ali A, Koutroumpakis E, Song J, Booser D, Barcenas CH, Tripathy D, et al Risk stratification for trastuzumab-induced cardiac dysfunction and potential implications for surveillance. JACC CardioOncol 2025;7:203–15. doi: 10.1016/j.jaccao.2024.12.007 40246379 PMC12046757

[20] Astarita A, Mingrone G, Airale L, Colomba A, Catarinella C, Cesareo M, et al Validation of the HFA-ICOS score for carfilzomib-induced cardiotoxicity in multiple myeloma: a real-life perspective study. Cancers (Basel) 2025;17:2353. doi: 10.3390/cancers17142353 40723237 PMC12293333

[21] Rivero-Santana B, Saldaña-García J, Caro-Codón J, Zamora P, Moliner P, Martínez Monzonis A, et al Anthracycline-induced cardiovascular toxicity: validation of the Heart Failure Association and International Cardio-Oncology Society risk score. Eur Heart J 2025;46:273–84. doi: 10.1093/eurheartj/ehae496 39106857

[22] Madaudo C, Di Lisi D, Cannatà A, Manfrè F, Vullo C, Santoro M, et al Cardiovascular toxicity induced by TKIs in patients with chronic myeloid leukaemia: are women and men different? ESC Heart Fail 2025;12:1447–54. doi: 10.1002/ehf2.15165 39780756 PMC11911597

[23] Jacobson TA, Peigh G, Patel R, Issa RP, Akhter N. Developing a simple clinical risk score for ibrutinib-associated atrial fibrillation. J Interv Card Electrophysiol 2025;68:933–42. doi: 10.1007/s10840-025-01990-4 40090956

[24] Soh CH, Marwick TH. Comparison of heart failure risk assessment tools among cancer survivors. Cardiooncology 2024;10:67. doi: 10.1186/s40959-024-00267-5 39394611 PMC11468191

[25] Di Lisi D, Madaudo C, Faro DC, Rossetto L, Triolo OF, Losi V, et al The added value of the HFA/ICOS score in the prediction of chemotherapy-related cardiac dysfunction in breast cancer. J Cardiovasc Med (Hagerstown) 2024;25:218–24. doi: 10.2459/jcm.0000000000001589 38305134

[26] Fernando F, Andres MS, Claudiani S, Kermani NZ, Ceccarelli G, Innes AJ, et al Cardiovascular events in CML patients treated with nilotinib: validation of the HFA-ICOS baseline risk score. Cardiooncology 2024;10:42. doi: 10.1186/s40959-024-00245-x 39010172 PMC11247904

[27] Tawfiq E, Selak V, Elwood JM, Pylypchuk R, Tin ST, Harwood M, et al Performance of cardiovascular disease risk prediction equations in more than 14 000 survivors of cancer in New Zealand primary care: a validation study. Lancet 2023;401:357–65. doi: 10.1016/s0140-6736(22)02405-9 36702148

[28] Cronin M, Crowley A, Davey MG, Ryan P, Abdelshafy M, Elkoumy A, et al Heart Failure Association-International Cardio-Oncology Society risk score validation in HER2-positive Breast Cancer. J Clin Med 2023;12:1278. doi: 10.3390/jcm12041278 36835818 PMC9963986

[29] Doukas PG, Cascino GJ, Meng Z, Baldridge AS, Kang Y, Scherrer-Crosbie M, et al External validation of a heart failure risk score in patients with acute myeloid leukemia. Leuk Lymphoma 2023;64:445–53. doi: 10.1080/10428194.2022.2140289 36331544

[30] Mery B, Rowinski E, Rivier C, Bouleftour W, Sotton S, Tinquaut F, et al Cardiovascular diseases following Breast Cancer: towards a case-by-case assessment through a prediction risk score model in 943 patients. Am J Clin Oncol 2022;45:155–60. doi: 10.1097/coc.0000000000000904 35320816

[31] Strongman H, Herrett E, Jackson R, Sweeting M, Lyon AR, Stanway S, et al Cancer history as a predictor in cardiovascular risk scores: a primary care cohort study. Br J Gen Pract 2023;73:e34–42. doi: 10.3399/bjgp.2022.0088 36443065 PMC9710863

[32] Jacobs JEJ, Guler I, Duchenne J, Janssens S, Van Aelst LNL. Predictability of cardiotoxicity: experience of a Belgian cardio-oncology clinic. Int J Cardiol 2022;363:119–22. doi: 10.1016/j.ijcard.2022.06.063 35777489

[33] Suntheralingam S, Fan CS, Calvillo-Argüelles O, Abdel-Qadir H, Amir E, Thavendiranathan P. Evaluation of risk prediction models to identify cancer therapeutics related cardiac dysfunction in women with HER2+ Breast Cancer. J Clin Med 2022;11:847. doi: 10.3390/jcm11030847 35160296 PMC8836544

[34] Leoce NM, Jin Z, Kehm RD, Roh JM, Laurent CA, Kushi LH, et al Modeling risks of cardiovascular and cancer mortality following a diagnosis of loco-regional breast cancer. Breast Cancer Res 2021;23:91. doi: 10.1186/s13058-021-01469-w 34579765 PMC8474887

[35] Battisti NML, Andres MS, Lee KA, Ramalingam S, Nash T, Mappouridou S, et al Incidence of cardiotoxicity and validation of the Heart Failure Association-International Cardio-Oncology Society risk stratification tool in patients treated with trastuzumab for HER2-positive early breast cancer. Breast Cancer Res Treat 2021;188:149–63. doi: 10.1007/s10549-021-06192-w 33818652

[36] Archibald WJ, Rabe KG, Kabat BF, Herrmann J, Ding W, Kay NE, et al Atrial fibrillation in patients with chronic lymphocytic leukemia (CLL) treated with ibrutinib: risk prediction, management, and clinical outcomes. Ann Hematol 2021;100:143–55. doi: 10.1007/s00277-020-04094-3 32488603 PMC8772341

[37] Caocci G, Mulas O, Abruzzese E, Luciano L, Iurlo A, Attolico I, et al Arterial occlusive events in chronic myeloid leukemia patients treated with ponatinib in the real-life practice are predicted by the systematic coronary risk evaluation (SCORE) chart. Hematol Oncol 2019;37:296–302. doi: 10.1002/hon.2606 30892724 PMC6766852

[38] Ferraro MP, Gimeno-Vazquez E, Subirana I, Gómez M, Díaz J, Sánchez-González B, et al Anthracycline-induced cardiotoxicity in diffuse large B-cell lymphoma: NT-proBNP and cardiovascular score for risk stratification. Eur J Haematol 2019;102:509–15. doi: 10.1111/ejh.13234 30972815

[39] Moey MYY, Liles DK, Carabello BA. Concomitant use of renin-angiotensin-aldosterone system inhibitors prevent trastuzumab-induced cardiotoxicity in HER2+ breast cancer patients: an institutional retrospective study. Cardiooncology 2019;5:9. doi: 10.1186/s40959-019-0043-8 32154015 PMC7048102

[40] Rushton M, Johnson C, Dent S. Trastuzumab-induced cardiotoxicity: testing a clinical risk score in a real-world cardio-oncology population. Curr Oncol 2017;24:176–80. doi: 10.3747/co.24.3349 28680277 PMC5486382

[41] Law W, Johnson C, Rushton M, Dent S. The framingham risk score underestimates the risk of cardiovascular events in the HER2-positive breast cancer population. Curr Oncol 2017;24:e348–53. doi: 10.3747/co.24.3684 29089804 PMC5659158

[42] Hu WS, Lin CL. Comparison of CHA(2)DS(2)-VASc, CHADS(2) and HATCH scores for the prediction of new-onset atrial fibrillation in cancer patients: a nationwide cohort study of 760,339 study participants with competing risk analysis. Atherosclerosis 2017;266:205–11. doi: 10.1016/j.atherosclerosis.2017.10.007 29049919

[43] Rea D, Mirault T, Raffoux E, Boissel N, Andreoli AL, Rousselot P, et al Usefulness of the 2012 European CVD risk assessment model to identify patients at high risk of cardiovascular events during nilotinib therapy in chronic myeloid leukemia. Leukemia 2015;29:1206–9. doi: 10.1038/leu.2014.342 25482133

[44] Breccia M, Molica M, Zacheo I, Serrao A, Alimena G. Application of systematic coronary risk evaluation chart to identify chronic myeloid leukemia patients at risk of cardiovascular diseases during nilotinib treatment. Ann Hematol 2015;94:393–7. doi: 10.1007/s00277-014-2231-9 25304102

[45] Gent DG, Rebecca D. The 2022 European Society of Cardiology Cardio-oncology guidelines in focus. Eur Cardiol 2023;18:e16. doi: 10.15420/ecr.2022.63 37405348 PMC10316349

[46] Darby SC, Ewertz M, McGale P, Bennet AM, Blom-Goldman U, Brønnum D, et al Risk of ischemic heart disease in women after radiotherapy for breast cancer. N Engl J Med 2013;368:987–98. doi: 10.1056/NEJMoa1209825 23484825

[47] Alexandre J, Font J, Angélique DS, Delapierre B, Damaj G, Plane AF, et al Is ibrutinib-related atrial fibrillation dose dependent? Insights from an individual case level analysis of the World Health Organization pharmacovigilance database. Leukemia 2024;38:2628–35. doi: 10.1038/s41375-024-02413-5 39300222 PMC11588646

[48] Blaes A, Nohria A, Armenian S, Bergom C, Thavendiranathan P, Barac A, et al Cardiovascular considerations after Cancer therapy: gaps in evidence and JACC: cardioOncology expert panel recommendations. JACC CardioOncol 2025;7:1–19. doi: 10.1016/j.jaccao.2024.06.006 39896126 PMC11782100

[49] Mandala E, Lafara K, Kokkinovasilis D, Kalafatis I, Koukoulitsa V, Katodritou E, et al Applied cardio-oncology in hematological malignancies: a narrative review. Life (Basel) 2024;14:524. doi: 10.3390/life14040524 38672794 PMC11050930

[50] Böttcher L, Felder S. Determining the optimal sequence of multiple tests. Stat Med 2025;44:e70254. doi: 10.1002/sim.70254 41105104 PMC12533563

[51] Collins GS, Reitsma JB, Altman DG, Moons KG. Transparent reporting of a multivariable prediction model for individual prognosis or diagnosis (TRIPOD): the TRIPOD statement. BMJ 2015;350:g7594. doi: 10.1136/bmj.g7594 25569120

[52] Ma Z, Caldwell R, Attia Z, Friedman P, Lerman A, Ng C, et al Harnessing artificial intelligence for cardio-oncology:towards a new future of cardiovascular care for the Cancer patient. Trends Cardiovasc Med 2026;36:389–99. doi: 10.1016/j.tcm.2026.01.006 41633441

[53] Al-Droubi SS, Jahangir E, Kochendorfer KM, Krive M, Laufer-Perl M, Gilon D, et al Artificial intelligence modelling to assess the risk of cardiovascular disease in oncology patients. Eur Heart J Digit Health 2023;4:302–15. doi: 10.1093/ehjdh/ztad031 37538144 PMC10393891

[54] Yagi R, Goto S, Himeno Y, Katsumata Y, Hashimoto M, MacRae CA, et al Artificial intelligence-enabled prediction of chemotherapy-induced cardiotoxicity from baseline electrocardiograms. Nat Commun 2024;15:2536. doi: 10.1038/s41467-024-45733-x 38514629 PMC10957877

[55] Li C, Liu X, Shen P, Sun Y, Zhou T, Chen W, et al Improving cardiovascular risk prediction through machine learning modelling of irregularly repeated electronic health records. Eur Heart J Digit Health 2024;5:30–40. doi: 10.1093/ehjdh/ztad058 38264696 PMC10802828

[56] Tsai ML, Chen KF, Chen PC. Harnessing electronic health records and artificial intelligence for enhanced cardiovascular risk prediction: a comprehensive review. J Am Heart Assoc 2025;14:e036946. doi: 10.1161/jaha.124.036946 40079336 PMC12132661

