# Supplementary material for: Cardiovascular risk–prediction models in cancer survivors: a meta-analysis
Source: Eur Heart J. 2026 Jul 28;47(35):4941–56. doi: 10.1093/eurheartj/ehag562 (PMC13577736; doi:10.1093/eurheartj/ehag562)
Supplement: Supplementary file 1 [file ehag562_supplementary_data.pdf]

Cardiovascular Risk Prediction Models in Cancer Survivors: A Meta-Analysis

Supplementary Material

Supplementary Methods 3

Search Strategy 3

PubMed 3

Embase 4

Scopus 5

Web of Science 6

Total Results 6

Eligibility Criteria 7

Inclusion Criteria 7

Exclusion Criteria 7

Quality Assessment 8

Data Extraction 8

Risk of Bias Assessment Across PROBAST Domains 9

Results 10

Supplementary Table 1. Demographic characteristics of included studies stratified by cardiovascular outcomes 10

Supplementary Table 2. Demographic characteristics and follow-up duration of included studies 11

Supplementary Table 3. Cardiovascular risk prediction models with predictor components 15

Supplementary Table 4. Cardiovascular outcome definitions 19

Subgroup Analysis – Patients with Breast Cancer 24

Supplementary Table 5. Cardiovascular risk prediction models classification metrics in patients with breast cancer 24

Supplementary Figure 2. The summary ROC curves and summary estimation points in patients with breast cancer 25

Subgroup Analysis – Patients with Hematologic Malignancies 26

Supplementary Table 6. Cardiovascular risk prediction models classification metrics in patients with hematologic malignancies 26

Supplementary Figure 3. The summary ROC curves and summary estimation points in patients with hematologic malignancies 27

Sensitivity Analysis 28

Supplementary Figure 4. Sensitivity analysis of prospective studies summarizing pooled discrimination performance for general and cancer-specific cardiovascular risk scores across outcomes 28

Supplementary Table 7. Sensitivity analysis comparing pooled AUCs of risk scores for predicting cardiovascular outcomes according to follow-up duration 29

Supplementary Table 8. Sensitivity analysis of AUC estimates after excluding studies with imputed standard errors 30

Meta-Regression 32

Supplementary Figure 5. Bubble plot of the meta-regression (median follow-up) for predicting vascular events 32

Supplementary Figure 6. Bubble plot of the meta-regression (median follow-up) for predicting CTRCD/heart failure .. 33

Supplementary Figure 7. Bubble plot of the meta-regression (median follow-up) for predicting arrhythmia 34

Supplementary Figure 8. Bubble plot of the meta-regression (median follow-up) for predicting composite cardiovascular outcome 35

Supplementary Figure 9. Bubble plot of the meta-regression (cohort mean age) for predicting vascular events 36

Supplementary Figure 10. Bubble plot of the meta-regression (cohort mean age) for predicting CTRCD/heart failure .. 37

Supplementary Figure 11. Bubble plot of the meta-regression (cohort mean age) for predicting arrhythmia 38

Supplementary Figure 12. Bubble plot of the meta-regression (cohort mean age) for predicting composite cardiovascular outcome 39

Quality Assessment 40

PROBAST Quality Assessment Results for Risk of Bias and Applicability 40

Supplementary Figure 13. Methodological quality summary of the included studies based on PROBAST criteria 41

Supplementary Figure 14. Methodological quality graph of the included studies based on PROBAST criteria 42

PRISMA 2020 Checklist 43

Supplementary Methods

Search Strategy

PubMed (July 16, 2025)

Search	Query	Results (No.)
#1	"cancer survivor"[Title/Abstract] OR "cancer survivors"[Title/Abstract] OR "cancer patient"[Title/Abstract] OR "cancer patients"[Title/Abstract] OR survivorship[Title/Abstract] OR "cancer survivorship"[Title/Abstract] OR "long-term cancer"[Title/Abstract] OR "post-cancer"[Title/Abstract] OR "cancer follow-up"[Title/Abstract] OR "follow-up cancer"[Title/Abstract] OR "cardio-oncology"[Title/Abstract] OR "breast cancer survivor"[Title/Abstract] OR "prostate cancer survivor"[Title/Abstract] OR "testicular cancer survivor"[Title/Abstract] OR "lymphoma survivor"[Title/Abstract] OR "leukemia survivor"[Title/Abstract] OR "breast cancer patient"[Title/Abstract] OR "hematologic malignancy"[Title/Abstract] OR "treatment-related cardiovascular"[Title/Abstract] OR "cancer-related cardiovascular"[Title/Abstract] OR "cancer treatment"[Title/Abstract] OR "cancer therapy"[Title/Abstract] OR "cardiotoxicity"[Title/Abstract]	464,687
#2	cardiovascular[Title/Abstract] OR "cardiovascular disease"[Title/Abstract] OR CVD[Title/Abstract] OR "heart disease"[Title/Abstract] OR "coronary artery disease"[Title/Abstract] OR CAD[Title/Abstract] OR atherosclerosis[Title/Abstract] OR stroke[Title/Abstract] OR "ischemic heart disease"[Title/Abstract] OR myocardial[Title/Abstract] OR infarction[Title/Abstract] OR MI[Title/Abstract] OR "heart failure"[Title/Abstract] OR HF[Title/Abstract] OR arrhythmia[Title/Abstract] OR "atrial fibrillation"[Title/Abstract] OR AF[Title/Abstract] OR "vascular event"[Title/Abstract] OR "major adverse cardiovascular events"[Title/Abstract] OR MACE[Title/Abstract] OR "Cardiovascular Diseases"[MeSH Terms]	3,625,326
#3	"risk score"[Title/Abstract] OR "risk prediction"[Title/Abstract] OR "prediction model"[Title/Abstract] OR "prognostic model"[Title/Abstract] OR "clinical risk score"[Title/Abstract] OR "cardiovascular risk algorithm"[Title/Abstract] OR "risk algorithm"[Title/Abstract] OR "clinical prediction rule"[Title/Abstract] OR "deep learning"[Title/Abstract] OR "machine learning"[Title/Abstract] OR "AI model"[Title/Abstract] OR "model performance"[Title/Abstract] OR "model validation"[Title/Abstract] OR "external validation"[Title/Abstract] OR discrimination[Title/Abstract] OR classification[Title/Abstract] OR "C-statistic"[Title/Abstract] OR AUC[Title/Abstract] OR "area under curve"[Title/Abstract] OR "net reclassification"[Title/Abstract] OR NRI[Title/Abstract] OR IDI[Title/Abstract] OR "decision curve analysis"[Title/Abstract] OR QRISK[Title/Abstract] OR Framingham[Title/Abstract] OR "Pooled Cohort Equation"[Title/Abstract] OR ASCVD[Title/Abstract] OR CHEMO-RADIAT[Title/Abstract] OR "Reynolds risk score"[Title/Abstract] OR MESA[Title/Abstract] OR SCORE2[Title/Abstract]	713,779
#4	#1 AND #2 AND #3	1,039

Embase (July 16, 2025)

Search	Query	Results (No.)
#1	'cancer survivor':ti,ab OR 'cancer survivors':ti,ab OR 'cancer patient':ti,ab OR 'cancer patients':ti,ab OR survivorship:ti,ab OR 'cancer survivorship':ti,ab OR 'long-term cancer':ti,ab OR 'post-cancer':ti,ab OR 'cancer follow-up':ti,ab OR 'follow-up cancer':ti,ab OR 'cardio-oncology':ti,ab OR 'breast cancer survivor':ti,ab OR 'prostate cancer survivor':ti,ab OR 'testicular cancer survivor':ti,ab OR 'lymphoma survivor':ti,ab OR 'leukemia survivor':ti,ab OR 'breast cancer patient':ti,ab OR 'hematologic malignancy':ti,ab OR 'treatment-related cardiovascular':ti,ab OR 'cancer-related cardiovascular':ti,ab OR 'cancer treatment':ti,ab OR 'cancer therapy':ti,ab OR cardiotoxicity:ti,ab	713,868
#2	cardiovascular:ti,ab OR "cardiovascular disease":ti,ab OR CVD:ti,ab OR "heart disease":ti,ab OR "coronary artery disease":ti,ab OR CAD:ti,ab OR atherosclerosis:ti,ab OR stroke:ti,ab OR "ischemic heart disease":ti,ab OR myocardial:ti,ab OR infarction:ti,ab OR MI:ti,ab OR "heart failure":ti,ab OR HF:ti,ab OR arrhythmia:ti,ab OR "atrial fibrillation":ti,ab OR AF:ti,ab OR "vascular event":ti,ab OR "major adverse cardiovascular events":ti,ab OR MACE:ti,ab OR 'cardiovascular disease'/exp	6,674,269
#3	"risk score":ti,ab OR "risk prediction":ti,ab OR "prediction model":ti,ab OR "prognostic model":ti,ab OR "clinical risk score":ti,ab OR "cardiovascular risk algorithm":ti,ab OR "risk algorithm":ti,ab OR "clinical prediction rule":ti,ab OR "deep learning":ti,ab OR "machine learning":ti,ab OR "AI model":ti,ab OR "model performance":ti,ab OR "model validation":ti,ab OR "external validation":ti,ab OR discrimination:ti,ab OR classification:ti,ab OR "C-statistic":ti,ab OR AUC:ti,ab OR "area under curve":ti,ab OR "net reclassification":ti,ab OR NRI:ti,ab OR IDI:ti,ab OR "decision curve analysis":ti,ab OR QRISK:ti,ab OR Framingham:ti,ab OR "Pooled Cohort Equation":ti,ab OR ASCVD:ti,ab OR CHEMO-RADIAT:ti,ab OR "Reynolds risk score":ti,ab OR MESA:ti,ab OR SCORE2:ti,ab	909,045
#4	#1 AND #2 AND #3	2,889

Scopus (July 16, 2025)

Search	Query	Results (No.)
#1	TITLE-ABS("cancer survivor") OR TITLE-ABS("cancer survivors") OR TITLE-ABS("cancer patient") OR TITLE-ABS("cancer patients") OR TITLE-ABS(survivorship) OR TITLE-ABS("cancer survivorship") OR TITLE-ABS("long-term cancer") OR TITLE-ABS("post-cancer") OR TITLE-ABS("cancer follow-up") OR TITLE-ABS("follow-up cancer") OR TITLE-ABS("cardio-oncology") OR TITLE-ABS("breast cancer survivor") OR TITLE-ABS("prostate cancer survivor") OR TITLE-ABS("testicular cancer survivor") OR TITLE-ABS("lymphoma survivor") OR TITLE-ABS("leukemia survivor") OR TITLE-ABS("breast cancer patient") OR TITLE-ABS("hematologic malignancy") OR TITLE-ABS("treatment-related cardiovascular") OR TITLE-ABS("cancer-related cardiovascular") OR TITLE-ABS("cancer treatment") OR TITLE-ABS("cancer therapy") OR TITLE-ABS(cardiotoxicity)	584,982
#2	TITLE-ABS(cardiovascular) OR TITLE-ABS("cardiovascular disease") OR TITLE-ABS(CVD) OR TITLE-ABS("heart disease") OR TITLE-ABS("coronary artery disease") OR TITLE-ABS(CAD) OR TITLE-ABS(atherosclerosis) OR TITLE-ABS(stroke) OR TITLE-ABS("ischemic heart disease") OR TITLE-ABS(myocardial) OR TITLE-ABS(infarction) OR TITLE-ABS(MI) OR TITLE-ABS("heart failure") OR TITLE-ABS(HF) OR TITLE-ABS(arrhythmia) OR TITLE-ABS("atrial fibrillation") OR TITLE-ABS(AF) OR TITLE-ABS("vascular event") OR TITLE-ABS("major adverse cardiovascular events") OR TITLE-ABS(MACE)	2,627,657
#3	TITLE-ABS("risk score") OR TITLE-ABS("risk prediction") OR TITLE-ABS("prediction model") OR TITLE-ABS("prognostic model") OR TITLE-ABS("clinical risk score") OR TITLE-ABS("cardiovascular risk algorithm") OR TITLE-ABS("risk algorithm") OR TITLE-ABS("clinical prediction rule") OR TITLE-ABS("deep learning") OR TITLE-ABS("machine learning") OR TITLE-ABS("AI model") OR TITLE-ABS("model performance") OR TITLE-ABS("model validation") OR TITLE-ABS("external validation") OR TITLE-ABS(discrimination) OR TITLE-ABS(classification) OR TITLE-ABS("C-statistic") OR TITLE-ABS(AUC) OR TITLE-ABS("area under curve") OR TITLE-ABS("net reclassification") OR TITLE-ABS(NRI) OR TITLE-ABS(IDI) OR TITLE-ABS("decision curve analysis") OR TITLE-ABS(QRISK) OR TITLE-ABS(Framingham) OR TITLE-ABS("Pooled Cohort Equation") OR TITLE-ABS(ASCVD) OR TITLE-ABS(CHEMO-RADIAT) OR TITLE-ABS("Reynolds risk score") OR TITLE-ABS(MESA) OR TITLE-ABS(SCORE2)	2,263,258
#4	#1 AND #2 AND #3	778

Web of Science (July 16, 2025)

Search	Query	Results (No.)
#1	TS=("cancer survivor") OR TS=("cancer survivors") OR TS=("cancer patient") OR TS=("cancer patients") OR TS=(survivorship) OR TS=("cancer survivorship") OR TS=("long-term cancer") OR TS=("post-cancer") OR TS=("cancer follow-up") OR TS=("follow-up cancer") OR TS=("cardio-oncology") OR TS=("breast cancer survivor") OR TS=("prostate cancer survivor") OR TS=("testicular cancer survivor") OR TS=("lymphoma survivor") OR TS=("leukemia survivor") OR TS=("breast cancer patient") OR TS=("hematologic malignancy") OR TS=("treatment-related cardiovascular") OR TS=("cancer-related cardiovascular") OR TS=("cancer treatment") OR TS=("cancer therapy") OR TS=(cardiotoxicity)	546,460
#2	TS=(cardiovascular) OR TS=("cardiovascular disease") OR TS=(CVD) OR TS=("heart disease") OR TS=("coronary artery disease") OR TS=(CAD) OR TS=(atherosclerosis) OR TS=(stroke) OR TS=("ischemic heart disease") OR TS=(myocardial) OR TS=(infarction) OR TS=(MI) OR TS=("heart failure") OR TS=(HF) OR TS=(arrhythmia) OR TS=("atrial fibrillation") OR TS=(AF) OR TS=("vascular event") OR TS=("major adverse cardiovascular events") OR TS=(MACE)	2,264,721
#3	TS=("risk score") OR TS=("risk prediction") OR TS=("prediction model") OR TS=("prognostic model") OR TS=("clinical risk score") OR TS=("cardiovascular risk algorithm") OR TS=("risk algorithm") OR TS=("clinical prediction rule") OR TS=("deep learning") OR TS=("machine learning") OR TS=("AI model") OR TS=("model performance") OR TS=("model validation") OR TS=("external validation") OR TS=(discrimination) OR TS=(classification) OR TS=("C-statistic") OR TS=(AUC) OR TS=("area under curve") OR TS=("net reclassification") OR TS=(NRI) OR TS=(IDI) OR TS=("decision curve analysis") OR TS=(QRISK) OR TS=(Framingham) OR TS=("Pooled Cohort Equation") OR TS=(ASCVD) OR TS=(CHEMO-RADIAT) OR TS=("Reynolds risk score") OR TS=(MESA) OR TS=(SCORE2)	1,776,270
#4	#1 AND #2 AND #3	971

Total Results (July 16, 2025)

**Data base**	**Results (No.)**
PubMed	1,039
Embase	2,889
Scopus	778
Web of Science	971
**Total**	**5,677**

Eligibility Criteria

Inclusion Criteria

We included original research articles that met the following criteria: (1) adult participants aged ≥18 years with a confirmed history of any cancer type; (2) reporting CV outcomes in cancer survivors without prior CV disease; (3) development and/or external validation of a multivariable risk-prediction model to estimate future CV risk in this population; and (4) publication in the English language.

Exclusion Criteria

Studies were excluded if they: (1) evaluated risk scores that were not externally validated beyond the derivation cohort; (2) focused on non-CV outcomes such as venous thromboembolism, pulmonary embolism, bleeding, or infection; (3) assessed polygenic or genetic-only risk scores without clinical or multimodal predictors; (4) were non-original publications (editorials, conference abstracts, reviews, or meta-analyses); or (5) lacked a clearly defined, reproducible outcome definition for model prediction.

Quality Assessment

Data Extraction

After the removal of duplicates, all retrieved records underwent title and abstract screening to identify studies focused on the development or external validation of cardiovascular risk prediction models in cancer survivors. Two authors (S.K. and A.V.) independently screened titles and abstracts based on the predefined eligibility criteria. Full texts of potentially eligible studies were then assessed for inclusion. Discrepancies at any stage were resolved through discussion and mutual consensus with a third author (M.SC.). The following predefined study-level variables were extracted: study design (retrospective or prospective), country, publication year, eligibility criteria, cancer type (breast cancer, hematologic malignancies, or mixed), cancer treatment, cardiovascular outcome, number of patients, age, sex, follow-up duration, risk score type (general cardiovascular risk score or risk score targeted to patients with cancer), measures of discrimination (area under the ROC curve (AUC), C-statistic, or C-index with its 95% confidence intervals), and risk score's cut-off point with its confusion matrix values (true positives, false positives, false negatives, and true negatives) for detection of cardiovascular outcome.

Risk of Bias Assessment Across PROBAST Domains

PROBAST assesses risk of bias across 4 domains: participants, predictors, outcome, and analysis. Judgments for each domain are categorized as low, high, or unclear risk of bias, with the first 3 domains also assessed for applicability to the review question. In the *Participants* domain, retrospective studies using baseline and follow-up echocardiographic data were generally rated as high risk due to selection bias. These studies frequently included narrowly defined patient cohorts, limiting generalizability. The *Predictors* domain focused on whether candidate variables were clearly defined, consistently measured, and applicable at the point of clinical use. In the *Outcome* domain, studies predicting CTRCD/heart failure or new-onset arrhythmia outcomes were commonly rated as high risk, especially when outcomes were evaluated with inconsistent protocols for follow-up imaging or rhythm monitoring. The absence of standardized, prospective definitions or timing of outcome assessment increased the potential for bias. The *Analysis* domain examined the adequacy of statistical methods, particularly in relation to sample size and model overfitting. A key PROBAST criterion concerns whether a sufficient number of events occurred for reliable model estimation. Following established recommendations, we considered an events-per-variable ratio of ≥10 as low risk of bias and <10 as high risk. Studies with low events-per-variable were considered at increased risk of model overfitting, especially in small cohorts or in those with a small number of events.

Supplementary Results

Results

Supplementary Table 1. Demographic characteristics of included studies stratified by cardiovascular outcomes

Outcomes	Number of studies[*]	Number of participants	Mean age ± SD, year	Female sex, %
Vascular event	11 studies	135,793	61.5±11.4	66.3%
CTRCD/Heart failure	16 studies	70,642	59.2±8.8	78.1%
Arrhythmia	5 studies	792,482	61.1±14.7	45.7%
Composite CV event	9 studies	79,301	60.5±12.7	77.4%

Abbreviations: CTRCD: cancer therapy-related cardiac dysfunction, CV: cardiovascular, SD: standard deviation.

* Please note that some studies reported the performance of multiple risk scores for predicting more than one clinical outcome; however, in this table, each study was counted only once within each outcome category.

Supplementary Table 2. Demographic characteristics, follow-up duration, inclusion and exclusion criteria of included studies

Study, year	Mean age±standard deviation, year	Female sex, %	Inclusion criteria	Exclusion criteria
A. Ali et al., 2025	52.3±13.2	100%	- HER2-positive breast cancer<br>- Neoadjuvant or adjuvant Trastuzumab treatment, planned for 1 year	- Lack of echocardiographic examination before and after trastuzumab initiation
A. Astarita et al., 2025	70.0	54.4%	- Age ≥18 years<br>- Multiple myeloma cancer<br>- Carfilzomib treatment	- Patients previously treated with Carfilzomib<br>- Other hematologic malignancies<br>- Cardiac amyloidosis<br>- Patients who did not experience cardiovascular events but still had ongoing Carfilzomib treatment at the end of this study were excluded because their outcome was not defined
B. Rivero-Santana et al., 2025	54±14	81.9%	- Any type of cancer<br>- Anthracycline-based chemotherapy<br>- expected life survival of >6 months	- Baseline LVEF <40%<br>- Missing both baseline and follow-up TTE
C. Madaudo et al., 2025	58.0±14.2	44.6%	- Age ≥18 years and <75 years<br>- Chronic myeloid leukemia cancer<br>- TKIs (imatinib, nilotinib, dasatinib, ponatinib, bosutinib and asciminib) treatment for at least 1 year<br>- Baseline LVEF >50%.	- Recent revascularization (<30 days)<br>- Severe concomitant diseases such as cerebrovascular diseases with sequelae<br>- Severe renal dysfunction (GFR <30 mL/min/1.73 m^2)<br>- Severe liver dysfunction (liver cirrhosis or Child-Pugh Class C)<br>- History of collagenopathy<br>- Moderate valvular defects<br>- Complex cardiac arrhythmias<br>- Life expectancy <1 year
T.A. Jacobson et al., 2025	65.9±11.1	30.0%	- Any type of cancer<br>- Ibrutinib treatment	- Prior history of atrial fibrillation
A. Abiodun et al., 2024	63.3±13.3	55.1%	- Age ≥18 years<br>- 5-fluorouracil or Capecitabine based chemotherapy	- Lack of 6-month follow-up data
C. McCracken et al., 2024	59.4±7.2	66.5%	- Age ≥18 years<br>- Any type of cancer	- Missing baseline data or lost to follow-up<br>- Pre-existing heart disease
C.H. Soh et al., 2024	59.5±7.0	86.3%	- Age ≥18 years<br>- Any type of cancer	- Missing baseline data or lost to follow-up<br>- Prior heart failure diagnosis
D. Di Lisi et al., 2024	56.0±10.7	100%	- Age ≥18 years<br>- Breast cancer<br>- Anthracyclines ± subsequential Trastuzumab treatment	- Severe renal or hepatic failure<br>- Inadequate echocardiographic acoustic window<br>- Previous radiotherapy

Study, year	Mean age±standard deviation, year	Female sex, %	Inclusion criteria	Exclusion criteria
E.J. Polter et al., 2024	71.9±8.6	46.1%	- Age ≥18 years<br>- Any type of cancer	- Races other than White or Black<br>- Pre-existing heart disease
F. Fernando et al., 2024	51.8±17.8	41.3%	- Chronic myeloid leukemia cancer<br>- Nilotinib treatment for any duration of time	- Missing baseline data or lost to follow-up
E. Tawfiq et al., 2023	60.4±8.5	57.0%	- Age ≥18 years<br>- Any type of cancer excluding squamous and basal cell skin cancer	- Pre-existing heart disease<br>- Renal failure (eGFR <30 mL/min per 1·73 m^2)<br>- Diabetes and eGFR <45 mL/min per 1·73 m^2<br>- Diabetic or overt nephropathy<br>- Developed cancer after the index date<br>- Diagnosed with cancer <2 years before index date<br>- Missing data on TC/HDL cholesterol
M. Cronin et al., 2023	NR	100%	- Age ≥18 years<br>- HER2-positive breast cancer<br>- ≥5 years of follow-up	- Missing baseline data or <5-years of follow-up
M.C. Tjong et al., 2023	Internal validation: 70.5±9.6<br>External validation: 66.3±11.1	Internal validation: 54.9%<br>External validation: 51.2%	- Age ≥18 years<br>- Lung cancer<br>- Radiotherapy treatment	—
O. Mulas et al., 2023	51.5±17.5	44.6%	- Age ≥18 years<br>- Chronic myeloid leukemia cancer<br>- Nilotinib and Ponatinib treatment	- Pre-existing heart disease<br>- History of diabetes
P.G. Doukas et al., 2023	54±14	43.5%	- Age ≥18 years<br>- Acute myeloid leukemia cancer<br>- Anthracycline-based treatment<br>- Available pretreatment TTE	- Did not receive Anthracycline chemotherapy<br>- Poor transthoracic echocardiogram image<br>- Missing baseline data or lost to follow-up
B. Mery et al., 2022	56.6	100%	- Age ≥18 years<br>- Breast cancer diagnosis<br>- ≥5 years of follow-up	- Breast cancer recurrence
H. Strongman et al., 2022	62.7±12.7	58.2%	- Age ≥18 years<br>- Any type of cancer	- Prior cardiovascular disease<br>- Current statin use
J.E.J. Jacobs et al., 2022	63.4±16.0	68.2%	- Age ≥18 years<br>- Any type of cancer	—

Study, year	Mean age±standard deviation, year	Female sex, %	Inclusion criteria	Exclusion criteria
S. Suntheralingam et al., 2022	52.4±10.9	100%	- Age ≥18 years<br>- HER2-positive breast cancer<br>- Trastuzumab ± Anthracycline treatment<br>- ≥3 follow-up echocardiographs	- Patients with stage IV cancer and those who were deemed palliative
N.M. Leoce et al., 2021	60.0	100%	- Age ≥18 years<br>- Stages I to III breast cancer	- Patients with stage IV cancer<br>- Recurrent cancer<br>- Missing baseline data or lost to follow-up
N.M.L. Battisti et al., 2021	54.3±11.9	100%	- Age ≥18 years<br>- HER2-positive breast cancer<br>- Trastuzumab treatment	- Patients who received part of the course of treatment in another centre<br>- Advanced-stage breast cancer
W.J. Archibald et al., 2020	66.0±14.5	29.5%	- Age ≥18 years<br>- Chronic lymphocytic leukemia cancer<br>- Ibrutinib treatment	—
G. Caocci et al., 2019	52.5±14.5	44.7%	- Age ≥18 years<br>- Chronic myeloid leukemia cancer<br>- Ponatinib treatment	—
H. Abdel-Qadir et al., 2019	61.0±14.8	100%	- Age ≥18 years<br>- Breast cancer	- Patients with in situ (Stage 0) and stage IV breast cancer<br>- Patients diagnosed with breast cancer on autopsy<br>- Distant lymph nodes or solid metastases within 180 days before or 365 days after the index date<br>- Residents of long-term care institutions<br>- Missing baseline data or lost to follow-up
M.P. Ferraro et al., 2019	65.7±15.6	49.2%	- Age ≥18 years<br>- Diffuse large B-cell lymphoma cancer<br>- Anthracycline-containing chemotherapy treatment	- Chemotherapy without anthracyclines<br>- Palliative treatment<br>- Overt or unequivocal heart failure<br>- Human immunodeficiency virus<br>- Eastern Cooperative Oncology Group performance status ≥4<br>- Life expectancy of <3 months
M.Y.Y. Moey et al., 2019	57±11	100%	- Age ≥18 years<br>- HER2-positive breast cancer<br>- Trastuzumab treatment	- Lacked baseline cardiac imaging or did not have a follow-up cardiac imaging at 2 to 4 months from baseline<br>- Pre-existing structural heart disease, valvular heart disease, congestive heart failure or cardiomyopathy with an EF < 50%
M. Rushton et al., 2017	56.0±11.7	100%	- Age ≥18 years<br>- Stages I to III breast cancer<br>- Adjuvant Trastuzumab treatment	- Patients with stage IV cancer<br>- Metastatic cancer diagnosis<br>- History of heart failure

Study, year	Mean age±standard deviation, year	Female sex, %	Inclusion criteria	Exclusion criteria
W. Law et al., 2017	58.5±13.5	100%	- Age ≥18 years<br>- HER2-positive breast cancer	- Anthracycline exposure before their breast cancer diagnosis<br>- Missing baseline data or lost to follow-up
WS. Hu et al., 2017	61.2±14.9	44.8%	- Age ≥18 years<br>- Any type of cancer	- History of atrial fibrillation
D. Rea et al., 2015	49.2±14.2	49.1%	- Age ≥18 years<br>- Chronic myeloid leukemia cancer<br>- Nilotinib treatment	—
M. Breccia et al., 2015	52.0±15.5	41.5%	- Age ≥18 years<br>- Chronic myeloid leukemia cancer<br>- Nilotinib treatment	—
G. Ezaz et al., 2014	73.6±5.3	100%	- Age ≥18 years<br>- Stages I to III breast cancer<br>- Adjuvant Trastuzumab treatment	- Pre-existing heart failure and cardiomyopathy<br>- Pre-existing cancer diagnosis<br>- chemotherapy or Trastuzumab initiated >9 months after surgery<br>- Failure to have continuous fee-for-service Medicare Part A or Part B coverage and at least one approved Medicare claim from 2 years before breast cancer diagnosis through death or end of follow-up<br>- Breast cancer diagnosis from autopsy or death certificate<br>- The month of breast cancer diagnosis was missing or the patient died during the month of diagnosis<br>- Tumor stage recorded as missing or histology was reported to be not of epithelial origin
E.H. Romond et al., 2012	49.0	100%	- Age ≥18 years<br>- HER2-positive breast cancer<br>- Normal baseline LVEF as assessed by multiple-gated acquisition scans	- Distant metastatic cancer disease<br>- Significant past or active cardiac disease

Abbreviations: EF: ejection fraction, ECOG: Eastern Cooperative Oncology Group, eGFR: estimated glomerular filtration rate, GFR: glomerular filtration rate, HDL: high-density lipoprotein, HER2: human epidermal growth factor receptor 2, HIV: human immunodeficiency virus, LVEF: left ventricular ejection fraction, TC: total cholesterol, TKI: tyrosine kinase inhibitor, TTE: transthoracic echocardiogram.

Supplementary Table 3. Cardiovascular risk prediction models with predictor components across included studies

Risk score	Number of studies	Predictive factors	
**General Cardiovascular Risk Scores (N=18)**			
SCORE	5	● Age<br>● Sex<br>● Smoking status	● Systolic blood pressure<br>● Total cholesterol
CHA_2DS_2-VASc	3	● Congestive heart failure / LV systolic dysfunction<br>● Hypertension<br>● Age 65-74 years<br>● Age ≥75 years	● Diabetes mellitus<br>● Prior Stroke / TIA / thromboembolism<br>● Vascular disease (e.g., MI, PAD, aortic plaque)<br>● Female sex
Framingham (Lipid-based)	3	● Age<br>● Sex<br>● Total cholesterol<br>● High-density lipoprotein cholesterol	● Systolic blood pressure<br>● Smoking status<br>● Diabetes mellitus
QRISK3	3	● Age<br>● Sex<br>● Total cholesterol/HDL-C ratio<br>● Systolic blood pressure<br>● Treatment for hypertension<br>● Smoking status<br>● BMI<br>● Diabetes mellitus<br>● Chronic kidney disease<br>● Atrial fibrillation<br>● Family history of premature CVD (first-degree relative <60 years)	● Ethnicity (9 categories)<br>● Deprivation score (Townsend index)<br>● Severe mental illness (e.g., schizophrenia, bipolar, moderate–severe depression)<br>● Systemic lupus erythematosus<br>● Migraine<br>● Rheumatoid arthritis<br>● Atypical antipsychotic medication use<br>● Oral corticosteroids (regular use)<br>● Erectile dysfunction (for men)<br>● Systolic blood pressure variability (standard deviation of recent readings)
SCORE2/SCORE-OP	3	● Age<br>● Sex<br>● Smoking status<br>● Systolic blood pressure	● Non-HDL cholesterol<br>● Risk region (calibration by country/region based on CVD mortality)<br>● eGFR and urine albumin-to-creatinine ratio
Framingham (BMI-based)	2	● Age<br>● Sex<br>● BMI	● Systolic blood pressure<br>● Smoking status<br>● Diabetes mellitus
10-y ASCVD/PCE	1	● Age<br>● Sex<br>● Race (White or African American)<br>● Total cholesterol<br>● HDL cholesterol	● Systolic blood pressure<br>● Status of hypertension treatment<br>● Smoking status<br>● Diabetes mellitus
ARIC-HF Score	1	● Age<br>● Sex	● Use of antihypertensive medication<br>● Diabetes mellitus

Risk score	Number of studies	Predictive factors	
		● Race/ethnicity<br>● Heart rate<br>● Systolic blood pressure	● Coronary heart disease<br>● Smoking status<br>● Body mass index
C_2HEST Score	1	● Age ≥75 years<br>● Coronary artery disease<br>● Chronic obstructive pulmonary disease	● Hypertension<br>● Systolic heart failure<br>● Thyroid disease (hyperthyroidism)
$CHADS_2$ Score	1	● Congestive heart failure or LV dysfunction<br>● Hypertension<br>● Age ≥ 75 years	● Diabetes mellitus<br>● Prior stroke or TIA
CHARGE-AF Score	1	● Age<br>● Race/ethnicity<br>● Height<br>● Weight (or BMI)<br>● Systolic blood pressure<br>● Diastolic blood pressure	● Current smoking status<br>● Antihypertensive medication use<br>● Diabetes mellitus<br>● Prior myocardial infarction<br>● Prior heart failure
CORE Score	1	● Age<br>● Sex<br>● Smoking status<br>● Systolic blood pressure	● Total cholesterol<br>● Diabetes mellitus<br>● History of coronary heart disease or prior MI
FRESCO Score	1	● Age<br>● Smoking status<br>● Diabetes mellitus	● Systolic blood pressure, stratified by hypertension treatment<br>● Total cholesterol and HDL cholesterol
$HARMS_2$-AF Score	1	● Hypertension<br>● Age 60-64 years<br>● Age ≥65 years<br>● BMI ≥30 kg/m²<br>● Male sex	● Sleep apnea<br>● Current smoking<br>● Alcohol intake 7-14 units/week<br>● Alcohol intake ≥15 units/week
HATCH Score	1	● Hypertension<br>● Age ≥75 years<br>● Prior stroke or TIA	● Chronic obstructive pulmonary disease<br>● Heart failure or systolic dysfunction
New Zealand CVD Score	1	● Age<br>● Ethnicity<br>● Socioeconomic deprivation index (NZDep quintiles)<br>● Family history of cardiovascular disease<br>● Smoking status<br>● History of atrial fibrillation<br>● Diabetes mellitus	● Systolic blood pressure<br>● Total cholesterol to HDL cholesterol ratio<br>● Preventive pharmacotherapy:<br>- Antihypertensive treatment<br>- Lipid-lowering therapy (e.g., statins)<br>- Antithrombotic use (e.g., aspirin)
PCP-HF Score	1	● Age<br>● Sex	● BMI<br>● Lipid profile (total cholesterol and HDL cholesterol)

Risk score	Number of studies	Predictive factors	
		● Race/ethnicity<br>● Systolic blood pressure<br>● Use of antihypertensive medication	● Smoking status<br>● Diabetes mellitus
Qstroke Score	1	● Age<br>● Sex<br>● Ethnic group<br>● BMI<br>● Smoking status<br>● Total cholesterol<br>● HDL cholesterol<br>● Systolic blood pressure<br>● Townsend deprivation index	● Atrial fibrillation<br>● Heart failure<br>● Ischemic heart disease<br>● Rheumatoid arthritis<br>● Chronic kidney disease (stage 3,4 or 5)<br>● Hypertension medication<br>● Diabetes mellitus<br>● Valvular heart disease<br>● Family history of heart disease
**Risk Scores Targeted to Patients with Cancer (N=9)**			
HFA-ICOS	11	**Very high factors:**<br>● Pre-existing HF or cardiomyopathy<br><br>**High factors:**<br>● Prior MI, prior CABG or PCI<br>● Stable angina<br>● Age ≥80<br>● Baseline LVEF <50%<br>● Prior thoracic radiotherapy or prior anthracycline exposure	**Medium factors:**<br>● Borderline LVEF 50–54%<br>● Age 65-79<br>● Hypertension<br>● Diabetes mellitus<br>● Chronic kidney disease (eGFR <60 mL/min/1.73 m²)<br>● Elevated baseline troponin or BNP/NT-proBNP<br>● Current or heavy former smoking<br>● Obesity (BMI >30)<br>● Prior non-anthracycline chemotherapy
Ezaz Score	4	● Age<br>● Adjuvant chemotherapy<br>● Coronary artery disease<br>● Atrial fibrillation or flutter	● Diabetes mellitus<br>● Hypertension<br>● Renal failure
Abdel-Qadir Risk Score	2	● Age<br>● Hypertension<br>● Diabetes mellitus	● Ischemic heart disease<br>● Atrial fibrillation<br>● Congestive heart failure
Italian AF Risk Score	2	● Age > 65 years<br>● Male sex<br>● Non-valvular cardiopathy (e.g. CAD)<br>● Valvular heart disease	● Hypo/hyperthyroidism<br>● Chronic lung disease<br>● Type 2 diabetes mellitus<br>● Grade ≥3 infections
Mayo CCL AF Score	2	● Age 65-74 years<br>● Age ≥75 years<br>● Male sex	● Valvular heart disease<br>● Hypertension
NSABP-31 Score	2	● Age<br>● Baseline LVEF	

Risk score	Number of studies	Predictive factors	
		● Antihypertensive medication use	
ASCO Score	1	● Age ≥65 years<br>● Cardiovascular disease history<br>● Hypertension<br>● Diabetes mellitus<br>● Chronic kidney disease (eGFR <60 mL/min/1.73 m²)<br>● Previous exposure to cardiotoxic therapy<br>● Smoking status<br>● Obesity	● Family history of premature CVD<br>● Anthracyclines use<br>● HER2-targeted therapies<br>● Tyrosine kinase inhibitors<br>● VEGF inhibitors<br>● Immune checkpoint inhibitors<br>● Chest (thoracic) radiation<br>● Carfilzomib
CHyLL Score	1	● Coronary heart disease<br>● Hypertension<br>● Ln (LADV15 + 1): Natural logarithm of the volume (%) of the left anterior descending artery receiving ≥15 Gray during radiation, plus 1	
Mayo Risk Score	1	● Age <15 or ≥ 65 years<br>● Female sex<br>● Hypertension<br>● Diabetes mellitus<br>● History of cardiomyopathy or heart failure	● Coronary artery disease<br>● Anthracycline-based chemotherapy<br>● Chest radiation<br>● Receipt of cardiotoxic drugs

Abbreviations: AF: Atrial fibrillation, ASCVD: Atherosclerotic cardiovascular disease, BMI: Body mass index, BNP: B-type natriuretic peptide, CABG: Coronary artery bypass grafting, CAD: Coronary artery disease, CHD: Coronary heart disease, CHF: Congestive heart failure, CKD: Chronic kidney disease, CVD: Cardiovascular disease, eGFR: Estimated glomerular filtration rate, HDL: High-density lipoprotein, HF: Heart failure, HER2: Human epidermal growth factor receptor 2, LAD: Left anterior descending (artery), LVEF: Left ventricular ejection fraction, MI: Myocardial infarction, NT-proBNP: N-terminal pro-B-type natriuretic peptide, PAD: Peripheral arterial disease, PCI: Percutaneous coronary intervention, TIA: Transient ischemic attack, TTE: Transthoracic echocardiogram, VEGF: Vascular endothelial growth factor.

Supplementary Table 4. Cardiovascular outcome definitions of included studies

Study, year	Outcome definition
A. Ali et al., 2025	CTRCD+HF: LVEF drop ≥10% to <53%, or ≥10% drop if baseline <50% (ASE/EACVI). Symptomatic CTRCD: clinical HF signs/symptoms
A. Astarita et al., 2025	Composite CV events: ACS, HF, arrhythmias, chest pain, syncope, sudden death, new LV dysfunction or GLS decline
B. Rivero-Santana et al., 2025	CTRCD+HF: per ESC 2022 — LVEF drop ≥10% to <50% or GLS decline >15%; may include therapy interruption or clinical HF
C. Madaudo et al., 2025	Vascular event: arterial thrombosis, VTE
T.A. Jacobson et al., 2025	Arrythmia: atrial fibrillation detected via ED, inpatient ECG/telemetry, outpatient ECG, ambulatory monitor, pacemaker, or unknown
A. Abiodun et al., 2024	Composite CV events: new angina, ACS, new arrhythmia, new HF, cardiac arrest (non-alternative), CV death
C. McCracken et al., 2024	Composite CV events: MI, stroke, HF, AF, cardiomyopathies, valvular disease, CV death
C.H. Soh et al., 2024	CTRCD+HF: HF diagnosis based on ICD-10 coding
D. Di Lisi et al., 2024	CTRCD+HF: ESC 2022 — LVEF drop ≥10% to <50% or >15% GLS drop; includes symptomatic and asymptomatic dysfunction
E.J. Polter et al., 2024	Vascular event: non-fatal MI, CHD death, ischemic stroke
F. Fernando et al., 2024	Vascular event: Ischemic: ACS, stroke, PVD<br>Composite CV events: arrhythmia, stroke, ACS, HF, syncope, PVD
E. Tawfiq et al., 2023	Composite CV events: hospitalizations or deaths from IHD, stroke, PVD, HF, other ischemic CVD.
M. Cronin et al., 2023	CTRCD+HF: LVEF drop ≥10%, or to <50%; includes HF (NYHA II-IV), trastuzumab discontinuation, or CV death.
M.C. Tjong et al., 2023	Composite CV events: unstable angina, HF visit, MI, revascularization, CV death.
O. Mulas et al., 2023	Vascular event: MI, angina, ischemic stroke, PAD

Study, year	Outcome definition
P.G. Doukas et al., 2023	CTRCD+HF: heart failure per 2017 cardiovascular/stroke endpoint definitions (symptoms+examination)
B. Mery et al., 2022	Composite CV events: AF, MI, HF, stroke, PAD, VTE
H. Strongman et al., 2022	Vascular event: coronary heart disease and cerebrovascular events
J.E.J. Jacobs et al., 2022	CTRCD+HF: LVEF drop ≥10% to below normal if asymptomatic, ≥5% with HF symptoms
S. Suntheralingam et al., 2022	CTRCD+HF: LVEF drop ≥10% to <55% (asymptomatic), or ≥5% to <55% with HF symptoms
N.M. Leoce et al., 2021	Vascular event: MI, CHD death, insufficiency, angina, stroke, PAD
N.M.L. Battisti et al., 2021	CTRCD+HF: LVEF drop ≥10% or to <50%, HF (NYHA II-IV), cardiac death, or trastuzumab interruption
W.J. Archibald et al., 2020	Arrythmia: atrial fibrillation identified via electronic medical records
G. Caocci et al., 2019	Vascular event: MI, stroke, PAD
H. Abdel-Qadir et al., 2019	Composite CV events: hospitalization for MI, angina, TIA, stroke, PVD, HF, or CV death
M.P. Ferraro et al., 2019	CTRCD: LVEF <55%, or ≥15% drop if baseline <55%, or clinical HF (CREC-modified)
M.Y.Y. Moey et al., 2019	CTRCD: LVEF drop >15% from baseline
M. Rushton et al., 2017	CTRCD: LVEF drop ≥10% to <50%
W. Law et al., 2017	Composite CV events: HF, arrhythmia, pericardial disease, MI, CV death
WS. Hu et al., 2017	Not reported
D. Rea et al., 2015	Vascular event: MI, PAD, stroke

Study, year	Outcome definition
M. Breccia et al., 2015	Vascular event: MI, unstable angina, or other clinical ischemic disease
G. Ezaz et al., 2014	CTRCD+HF: ≥1 inpatient or 2 outpatient ICD-9 codes ≥30 days apart
E.H. Romond et al., 2012	CTRCD+HF: CV death or CHF with dyspnea + LVEF drop ≥10% to <55%, or >5% drop to below normal limits

Abbreviations: ACS: acute coronary syndrome, AF: atrial fibrillation, ASE: American Society of Echocardiography, CHD: coronary heart disease, CHF: congestive heart failure, CREC: Cardiac Review and Evaluation Committee, CV: cardiovascular, CTRCD: cancer therapy-related cardiac dysfunction, EACVI: European Association of Cardiovascular Imaging, ECG: electrocardiogram, ED: emergency department, ESC: European Society of Cardiology, GLS: global longitudinal strain, HF: heart failure, ICD: International Classification of Diseases, IHD: ischemic heart disease, LV: left ventricle, LVEF: left ventricular ejection fraction, MI: myocardial infarction, NYHA: New York Heart Association, PAD: peripheral artery disease, PVD: peripheral vascular disease, TIA: transient ischemic attack, VTE: venous thromboembolism.

Supplementary Figure 1. The summary ROC curves and summary estimation points for cardiovascular risk prediction models in patients with any cancer

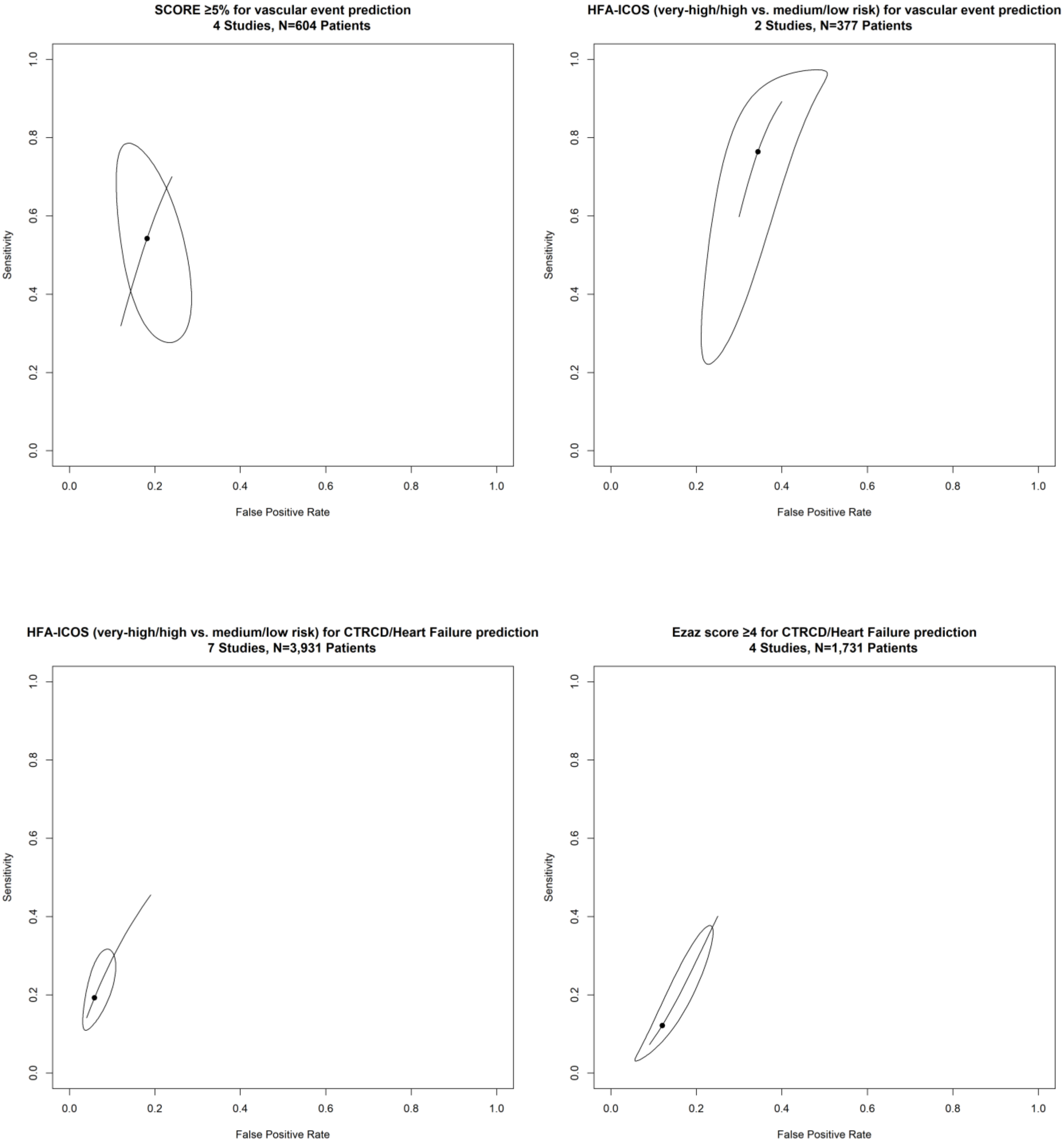

Framingham (Lipid-based) ≥20% for composite CV event prediction
2 Studies, N=31,686 Patients

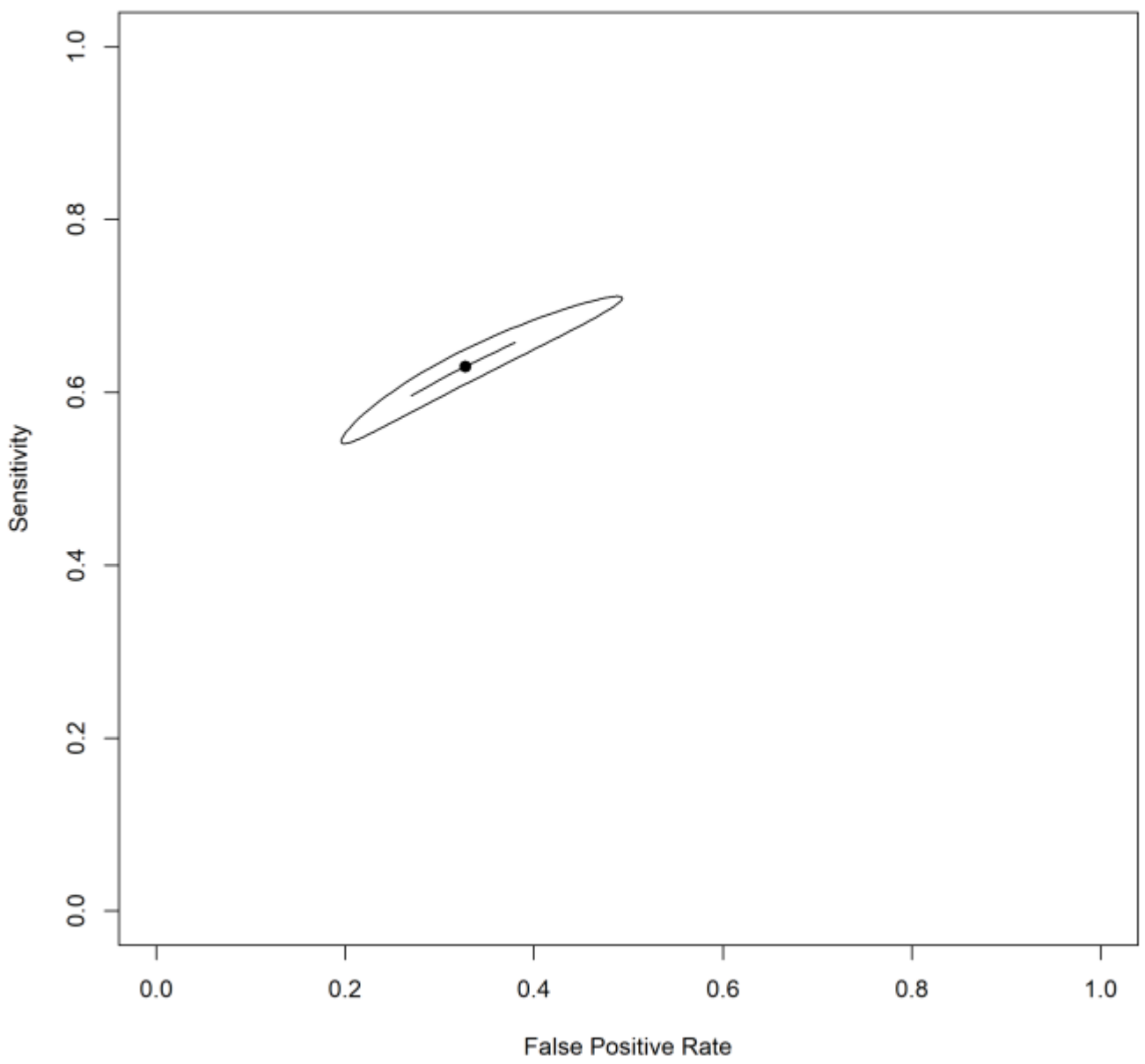

QRISK3 ≥10% for composite CV event prediction
2 Studies, N=33,432 Patients

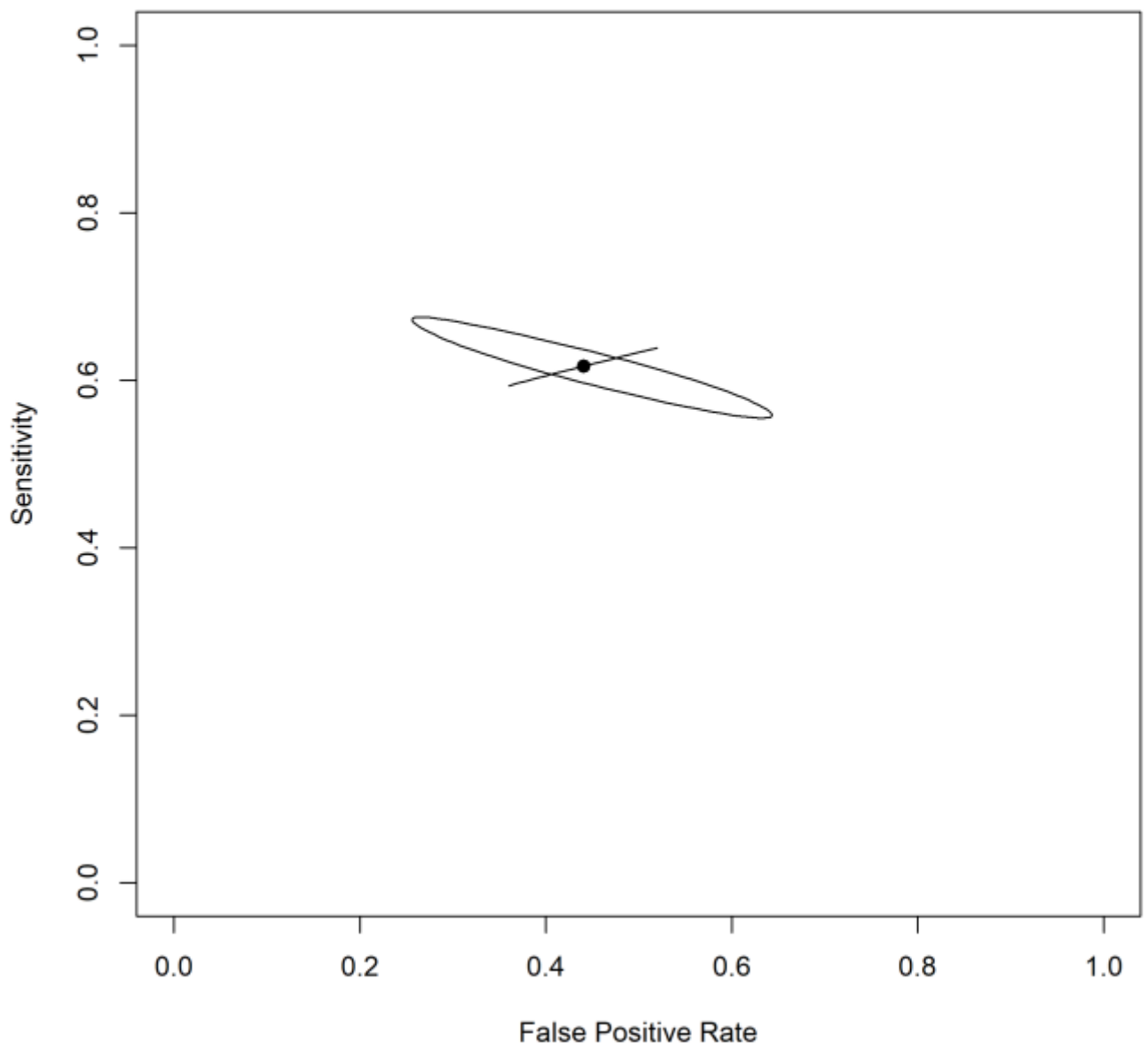

CHyLL score ≥5 for composite CV event prediction
2 Studies, N=303 Patients

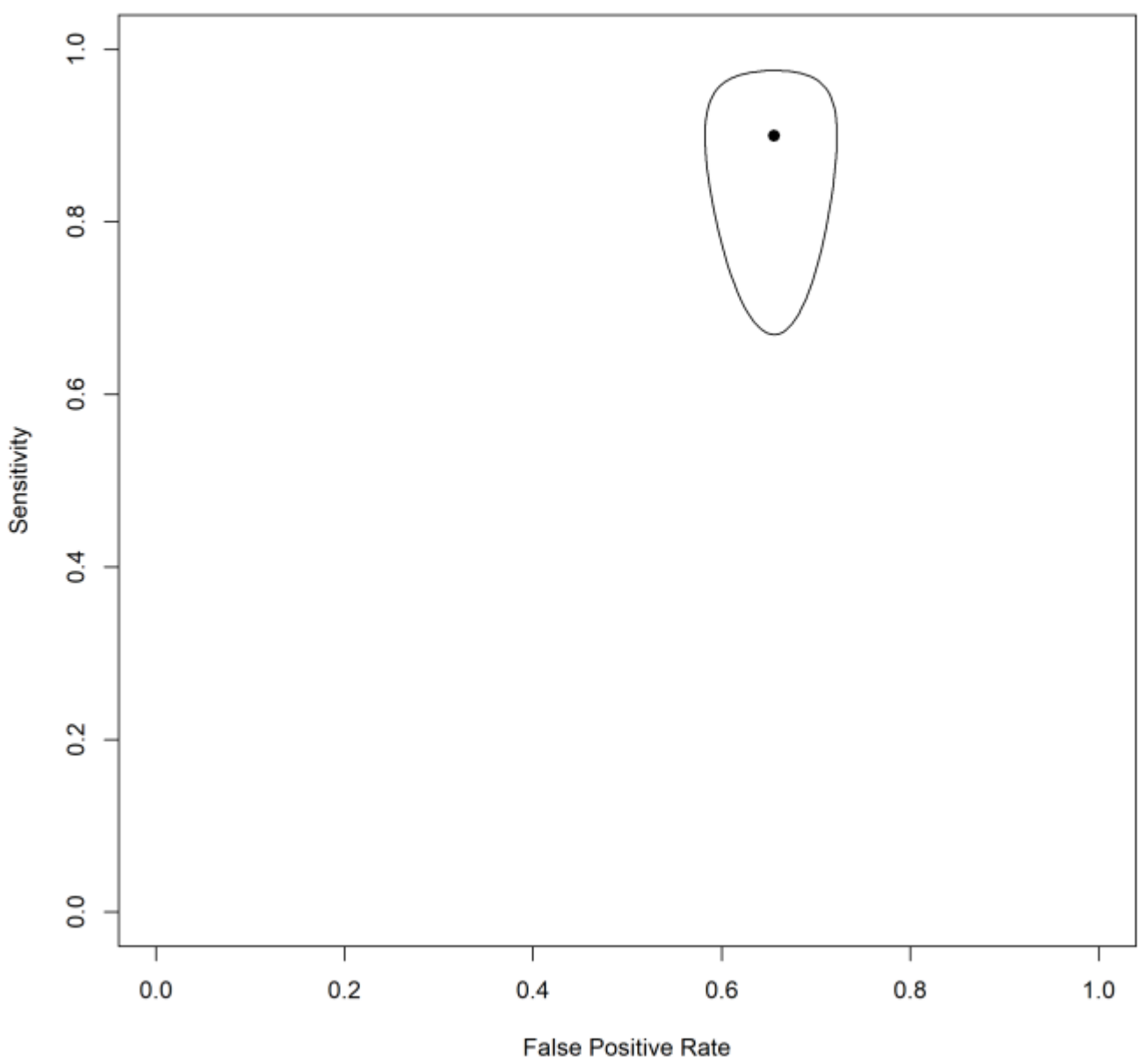

HFA-ICOS (very-high/high vs. medium/low risk) for composite CV event prediction
2 Studies, N=398 Patients

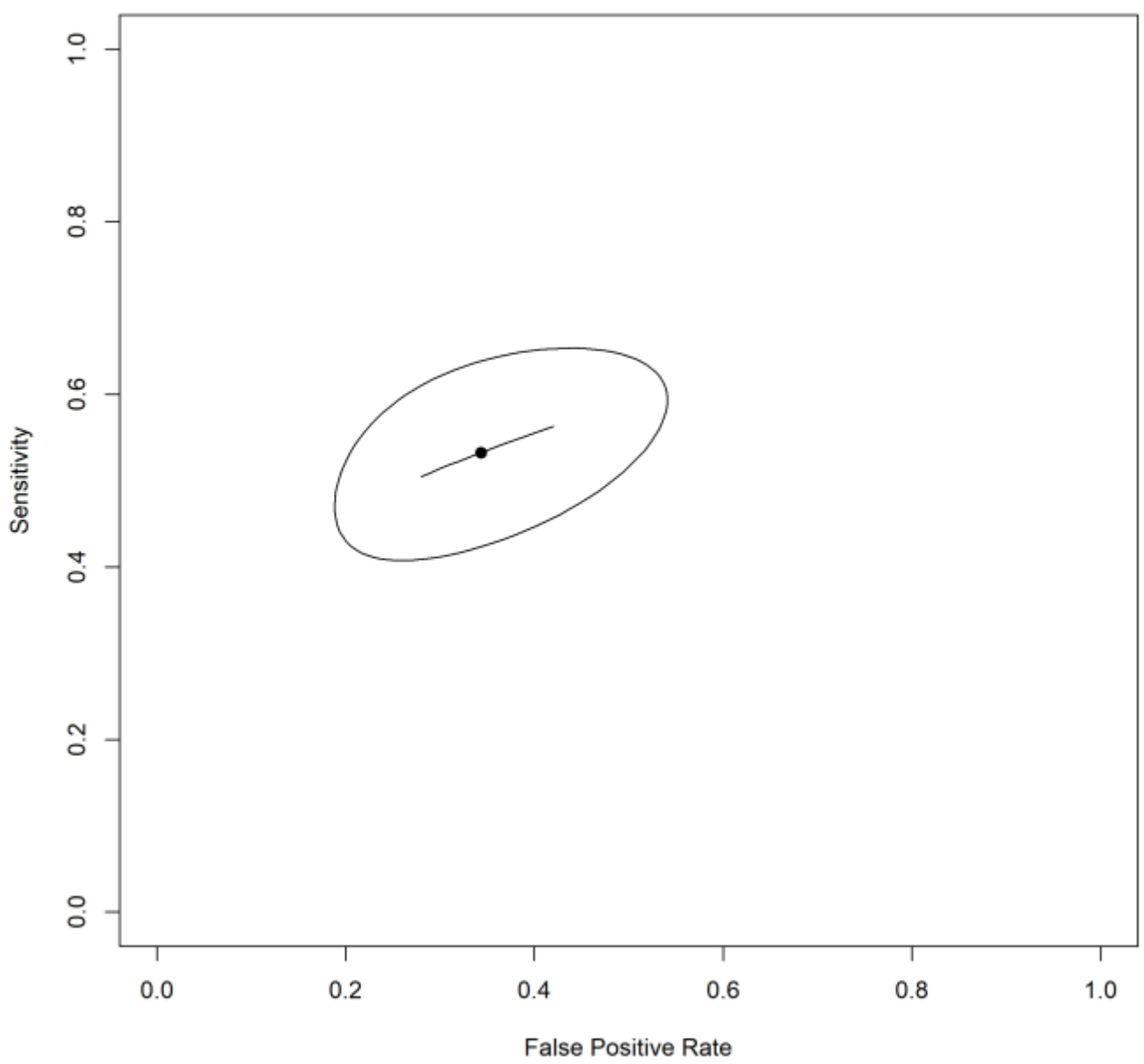

Subgroup Analysis – Patients with Breast Cancer

Supplementary Table 5. Cardiovascular risk prediction models classification metrics (sensitivity, specificity, and accuracy) in patients with breast cancer

Outcomes	Risk scores	No. of studies, patients	Sensitivity	Specificity	Accuracy
**General Cardiovascular Risk Scores**					
Vascular Event	Framingham (Lipid-based) ≥20%	1 Study, N=152	50.0%	69.3%	69.1%
	Qstroke Score (high vs. low risk)	1 Study, N=10,167	66.9%	61.9%	62.0%
	CHA_2DS_2-VASc Score ≥2	1 Study, N=10,167	52.3%	56.1%	56.0%
CTRCD/Heart Failure	PCP-HF Score (high vs. low risk)	1 Study, N=10,167	72.9%	65.0%	65.1%
	Framingham (Lipid-based) ≥20%	1 Study, N=152	62.5%	70.8%	70.4%
Arrhythmia	CHARGE-AF Score (high vs. low risk)	1 Study, N=10,167	69.0%	64.8%	65.0%
	Framingham (Lipid-based) ≥20%	1 Study, N=152	60.0%	70.1%	69.7%
Composite CV event	Framingham (Lipid-based) ≥20%	2 Studies, N=10,319	63.0%	67.3%	62.2%
	QRISK3 ≥10%	1 Study, N=10,167	63.9%	64.0%	64.0%
	Framingham (BMI-based) ≥20%	1 Study, N=10,167	66.9%	62.6%	63.0%
	SCORE2/SCORE-OP ≥7.5%	1 Study, N=10,167	66.0%	62.7%	63.0%
**Risk Scores Targeted to Patients with Cancer**					
CTRCD/Heart Failure	HFA-ICOS (very-high/high vs. medium/low risk)	4 Studies, N=2,563	18.2%	92.7%	81.2%
	Ezaz Score ≥4	4 Studies, N=1,731	12.2%	88.0%	69.8%
	NSABP-31 Score ≥65	1 Study, N=629	40.4%	70.5%	63.3%

Abbreviations: AF: atrial fibrillation, BMI: body mass index, CHARGE-AF: Cohorts for Heart and Aging Research in Genomic Epidemiology-Atrial Fibrillation, CTRCD: cancer therapy-related cardiac dysfunction, CV: cardiovascular, HFA-ICOS: Heart Failure Association-International Cardio-Oncology Society, HF: heart failure, NSABP-31: National Surgical Adjuvant Breast and Bowel Project trial 31, PCP-HF: Pooled Cohort Equations to Prevent Heart Failure, SCORE2/SCORE-OP: Systematic COronary Risk Evaluation 2 / SCORE-Older Persons.

* The highest values of sensitivity, specificity, and accuracy for each outcome are highlighted in red.

Supplementary Figure 2. The summary ROC curves and summary estimation points for cardiovascular risk prediction models in patients with breast cancer

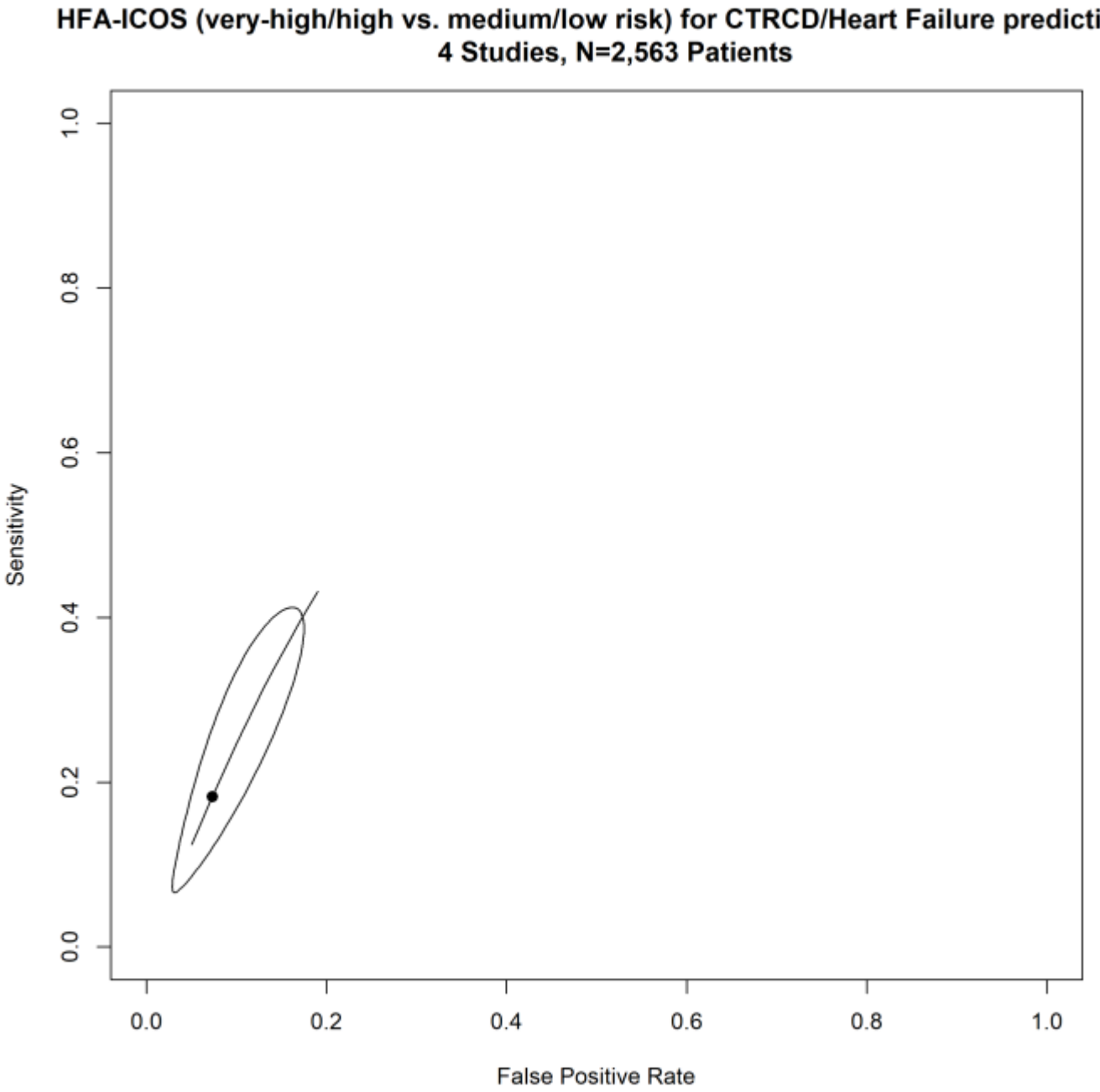

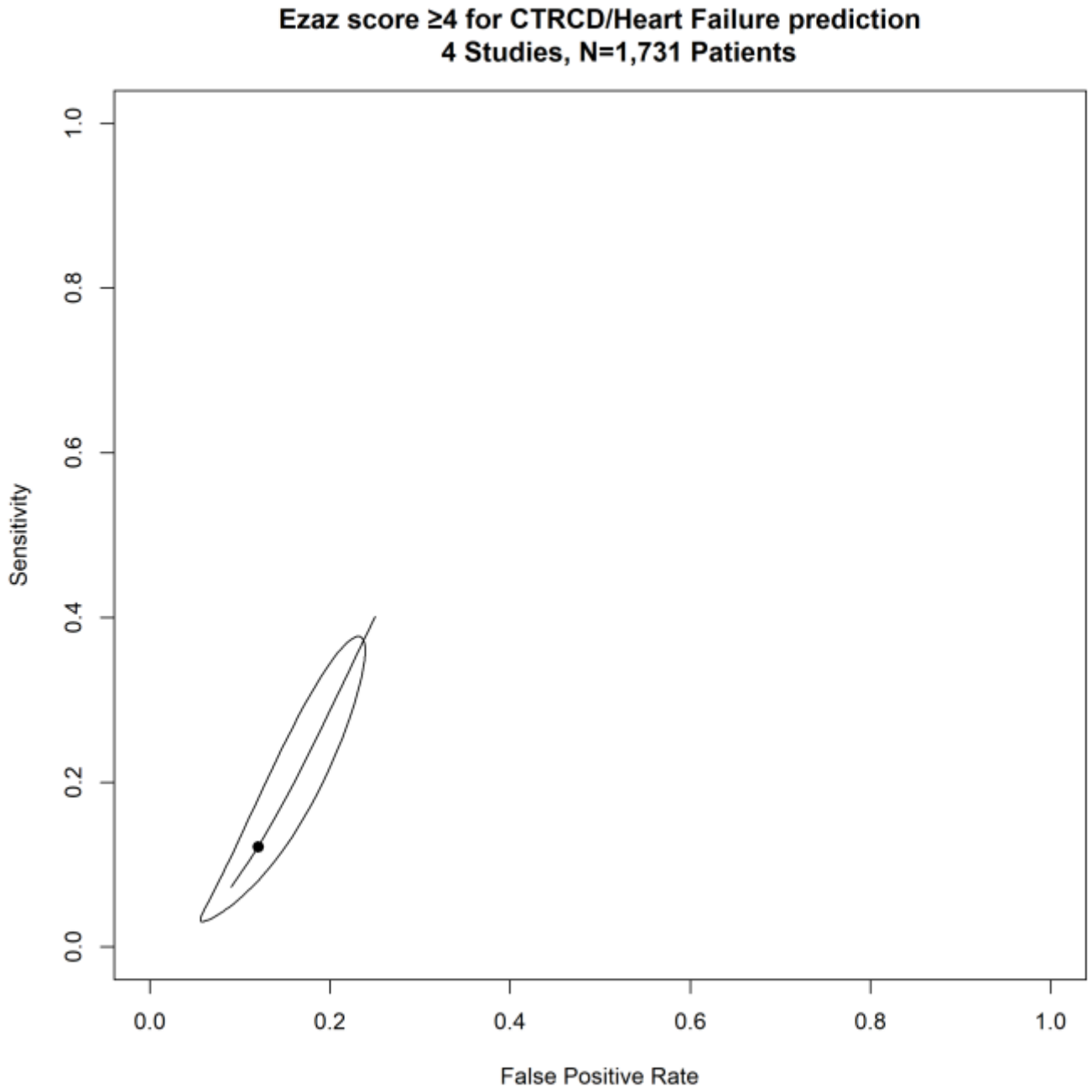

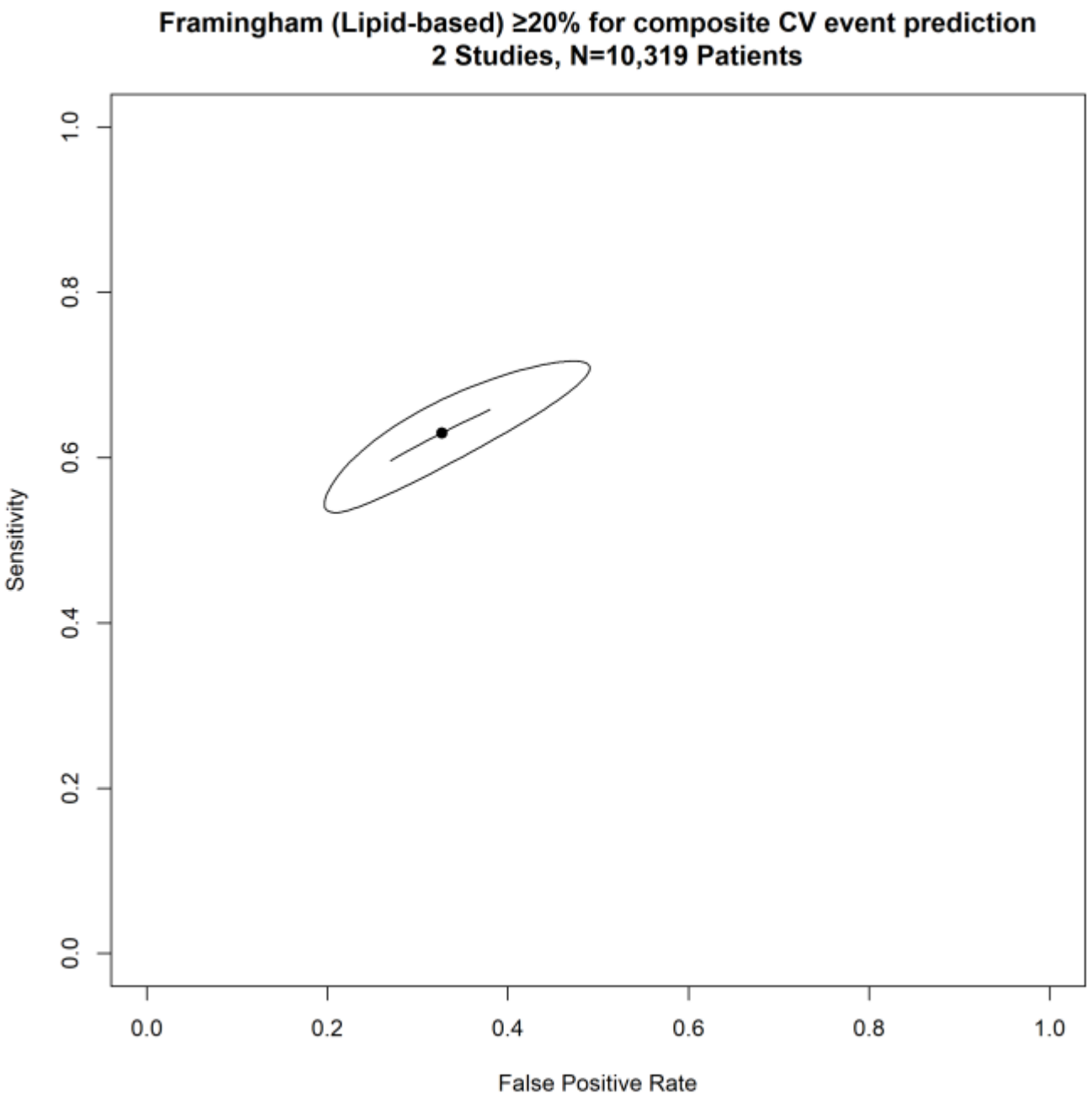

Subgroup Analysis – Patients with Hematologic Malignancies

Supplementary Table 6. Cardiovascular risk prediction models classification metrics (sensitivity, specificity, and accuracy) in patients with hematologic malignancies

Outcomes	Risk scores	No. of studies, patients	Sensitivity	Specificity	Accuracy
**General Cardiovascular Risk Scores**					
Vascular Event	SCORE ≥5%	4 Studies, N=604	54.2%	81.9%	77.6%
	SCORE2/SCORE-OP ≥7.5%	1 Study, N=380	60.6%	50.7%	51.6%
	Qstroke Score (high vs. low risk)	1 Study, N=2,538	46.5%	57.4%	57.0%
	CHA_2DS_2-VASc Score ≥2	1 Study, N=2,538	47.7%	56.3%	56.0%
CTRCD/Heart Failure	PCP-HF Score (high vs. low risk)	1 Study, N=2,538	47.3%	59.8%	59.0%
Arrhythmia	CHARGE-AF Score (high vs. low risk)	1 Study, N=2,538	53.0%	62.9%	62.0%
	Framingham (BMI-based) ≥20%	1 Study, N=290	33.3%	85.4%	76.2%
Composite CV event	Framingham (Lipid-based) ≥20%	1 Study, N=2,538	58.9%	61.5%	61.0%
	QRISK3 ≥10%	1 Study, N=2,538	55.9%	62.3%	61.0%
	Framingham (BMI-based) ≥20%	1 Study, N=2,538	52.9%	61.9%	60.0%
	SCORE2/SCORE-OP ≥7.5%	1 Study, N=2,538	52.9%	61.9%	60.0%
**Risk Scores Targeted to Patients with Cancer**					
Vascular Event	HFA-ICOS (very-high/high vs. medium/low risk)	2 Studies, N=377	76.4%	65.6%	69.2%
CTRCD/Heart Failure	HFA-ICOS (very-high/high vs. medium/low risk)	1 Study, N=193	14.7%	96.9%	82.4%
Arrhythmia	Italian AF Score ≥5	1 Study, N=290	35.3%	92.1%	82.1%
	Mayo CCL AF Score ≥4	1 Study, N=290	64.7%	66.1%	65.9%
Composite CV event	HFA-ICOS (very-high/high vs. medium/low risk)	2 Studies, N=398	53.3%	65.7%	62.7%

Abbreviations: AF: atrial fibrillation, BMI: body mass index, CHARGE-AF: Cohorts for Heart and Aging Research in Genomic Epidemiology-Atrial Fibrillation, CTRCD: cancer therapy-related cardiac dysfunction, CV: cardiovascular, HFA-ICOS: Heart Failure Association-International Cardio-Oncology Society, HF: heart failure, NSABP-31: National Surgical Adjuvant Breast and Bowel Project trial 31, PCP-HF: Pooled Cohort Equations to Prevent Heart Failure, SCORE2/SCORE-OP: Systematic COronary Risk Evaluation 2 / SCORE-Older Persons.

* The highest values of sensitivity, specificity, and accuracy for each outcome are highlighted in red.

Supplementary Figure 3. The summary ROC curves and summary estimation points for cardiovascular risk prediction models in patients with hematologic malignancies

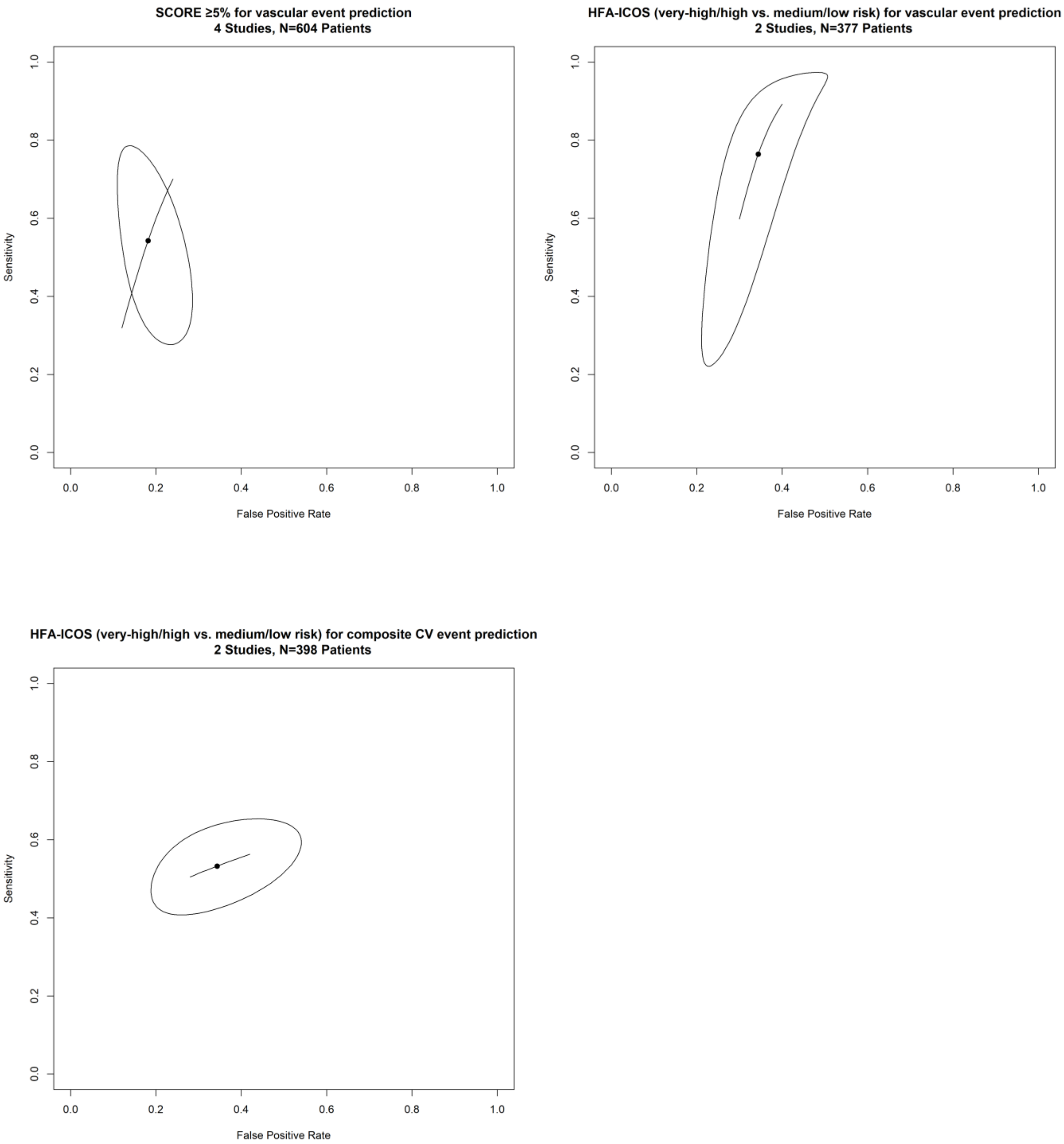

Sensitivity Analysis

Supplementary Figure 4. Sensitivity analysis of prospective studies summarizing pooled discrimination performance (AUC/C-index with 95% CI) for general and cancer-specific cardiovascular risk scores across outcomes

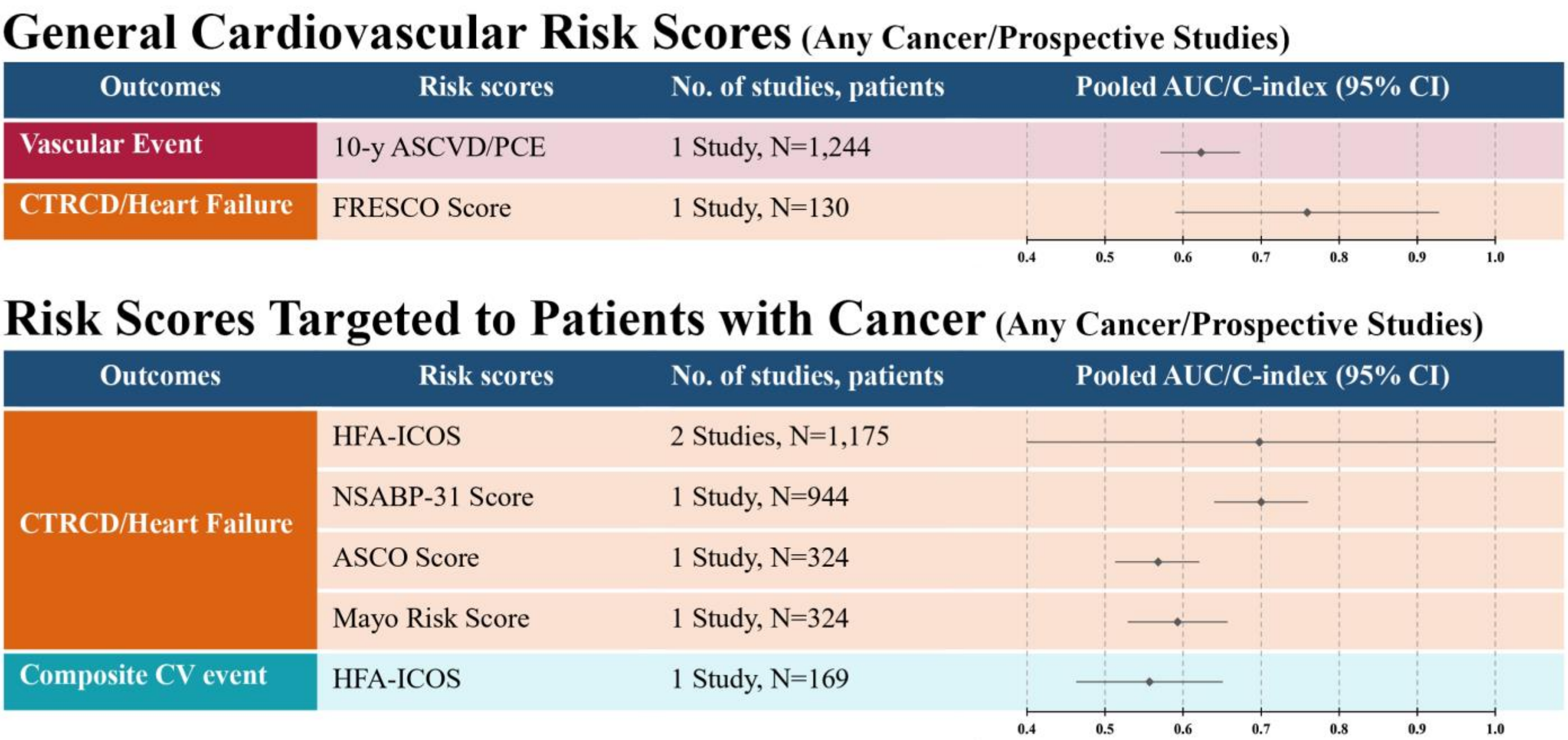

Abbreviations: ASCVD: Atherosclerotic cardiovascular disease, AUC: area under the curve, CTRCD: cancer therapy-related cardiac dysfunction, CI: confidence interval, CV: cardiovascular, HFA-ICOS: Heart Failure Association-International Cardio-Oncology Society, PCE: pooled cohort equation.

Supplementary Table 7. Sensitivity analysis comparing pooled AUCs of risk scores for predicting cardiovascular outcomes according to follow-up duration (>1 year vs. ≤1 year follow-up results)

Risk Score	Predicting Outcome	> 1-Year Follow-up Results		≤ 1-Year Follow-up Results		P-value*
		No. of Studies, Patients	Pooled AUC (95% CI)	No. of Studies, Patients	Pooled AUC (95% CI)	
HFA-ICOS	CTRCD/Heart Failure	5 studies, N=35,730	0.70 (0.59-0.80)	3 studies, N=1,233	0.59 (0.45-0.72)	0.019
HFA-ICOS	Composite CV event	1 study, N=229	0.61 (0.52-0.71)	1 study, N=169	0.56 (0.46-0.65)	0.460

* P-value was calculated using a Z-test for comparison of independent pooled AUCs between >1-year and ≤1-year follow-up periods.

Abbreviations: AUC: area under the curve, CI: confidence interval, CV: cardiovascular, HFA-ICOS: Heart Failure Association-International Cardio-Oncology Society, HF: heart failure.

Supplementary Table 8. Sensitivity analysis of AUC estimates after excluding studies with imputed standard errors

Studies with Imputed SE*	Risk Score	Predicting Outcome	All Studies (No. Studies, Patients)	Overall Estimate AUC (95% CI)	Sensitivity Analysis after Excluding Study with Imputed SE		
					After Exclusion Estimate AUC (95% CI)†	Δ AUC	P-value‡
Strongman et al., 2022	QRISK3	Vascular event	1 study, N=81,420	0.74 (0.73-0.75)	Not feasible	—	—
Romond et al., 2012	NSABP-31 Score	CTRCD/Heart Failure	2 studies, N=1,573	0.65 (0.45-0.85)	0.60 (0.55-0.65)	-0.05	0.635
Ferraro et al., 2019	FRESCO Score	CTRCD/Heart Failure	1 study, N=130	0.76 (0.59-0.93)	Not feasible	—	—
Jacobson et al., 2025	CHA_2DS_2-VASc	Arrhythmia	2 studies, N=760,506	0.68 (0.58-0.78)	0.68 (0.68-0.69)	0	1.000
Jacobson et al., 2025	Italian AF Score	Arrhythmia	2 studies, N=457	0.71 (0.42-1.00)	0.73 (0.65-0.80)	+0.02	0.896
Jacobson et al., 2025	Mayo CCL AF Score	Arrhythmia	2 studies, N=457	0.69 (0.59-0.80)	0.70 (0.63-0.77)	+0.01	0.877
Jacobson et al., 2025	C_2HEST Score	Arrhythmia	1 study, N=167	0.69 (0.60-0.78)	Not feasible	—	—
Jacobson et al., 2025	$HARMS_2$-AF	Arrhythmia	1 study, N=167	0.72 (0.63-0.81)	Not feasible	—	—
Astarita et al., 2025	HFA-ICOS	Composite CV event	2 studies, N=398	0.58 (0.24-0.93)	0.61 (0.52-0.71)	+0.03	0.870
Mery et al., 2022	Abdel-Qadir Score	Composite CV event	2 studies, N=30,753	0.73 (0.46-1.00)	0.80 (0.79-0.81)	+0.07	0.612

* Studies were classified as having imputed SEs, when 95% CI or SEs for the AUC were not reported in the original publication and were therefore imputed from studies with comparable sample size and event rate.

† Sensitivity analyses were performed by excluding studies with imputed SEs. When fewer than 2 studies remained after exclusion, pooled AUC estimation was not feasible.

‡ P-values compare the overall pooled AUC estimate with the new AUC estimate after exclusion of studies with imputed standard errors, using an approximate Z-test. P-values were calculated only when pooled estimates were available for both analyses.

Abbreviations: AUC: area under the receiver operating characteristic curve; CI: confidence interval; CTRCD: cancer therapy-related cardiac dysfunction; CV: cardiovascular; HFA-ICOS: Heart Failure Association–International Cardio-Oncology Society; SE: standard error.

Meta-Regression

Supplementary Figure 5. Bubble plot of the meta-regression assessing the association between median follow-up duration (months) and discriminative ability (AUC) of cardiovascular risk scores for predicting vascular events

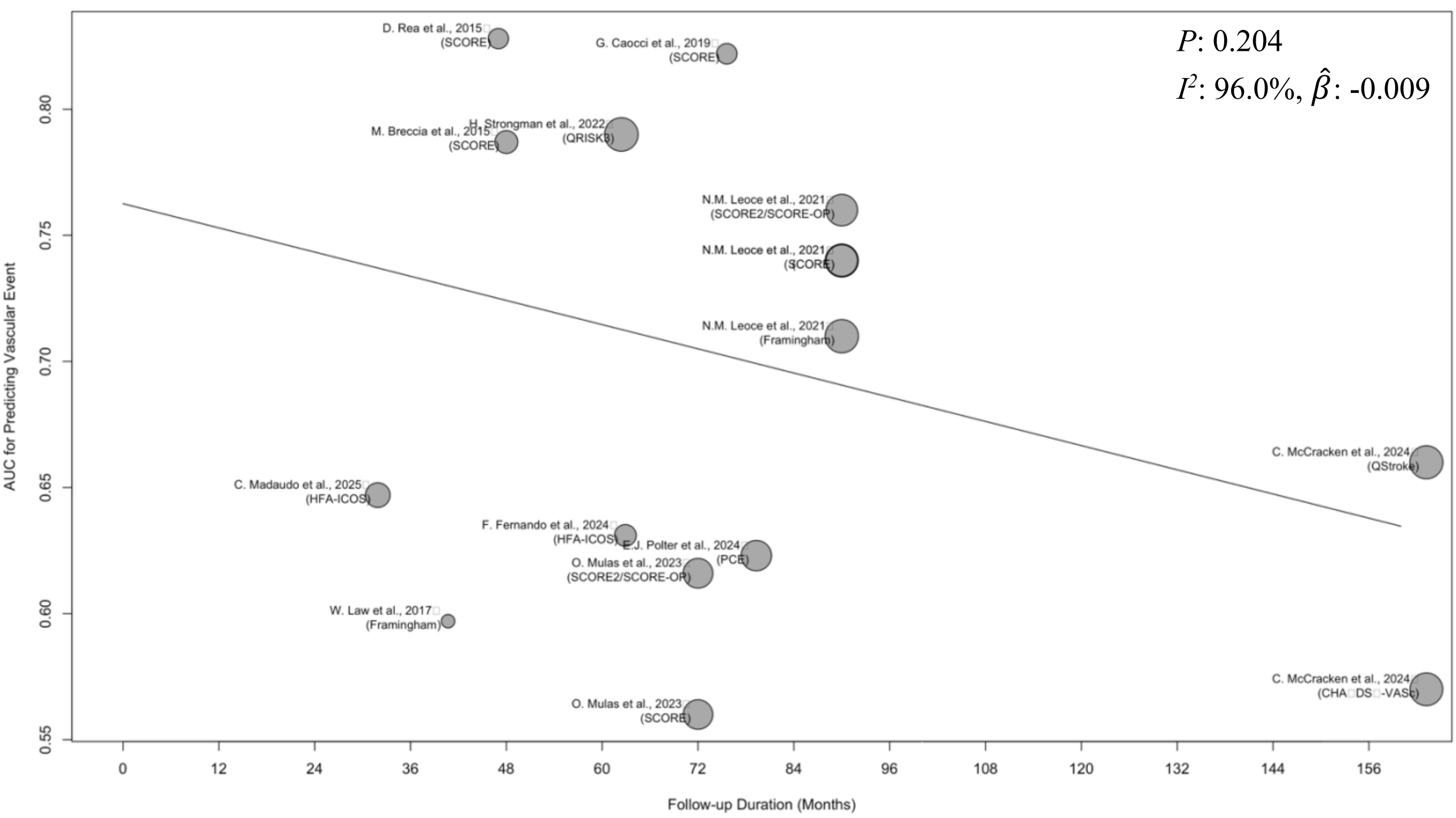

Each bubble represents a study; bubble size is proportional to study weight in the meta-regression. The solid line depicts the fitted regression line.

Meta-regression estimated regression coefficient ($\hat{\beta}$) represents the expected change in the AUC of cardiovascular risk scores for predicting vascular events associated with each additional year of median follow-up duration. I^2 represents residual heterogeneity between-study variation after accounting for the moderator in the meta-regression model.

Supplementary Figure 6. Bubble plot of the meta-regression assessing the association between median follow-up duration (months) and discriminative ability (AUC) of cardiovascular risk scores for predicting CTRCD/heart failure

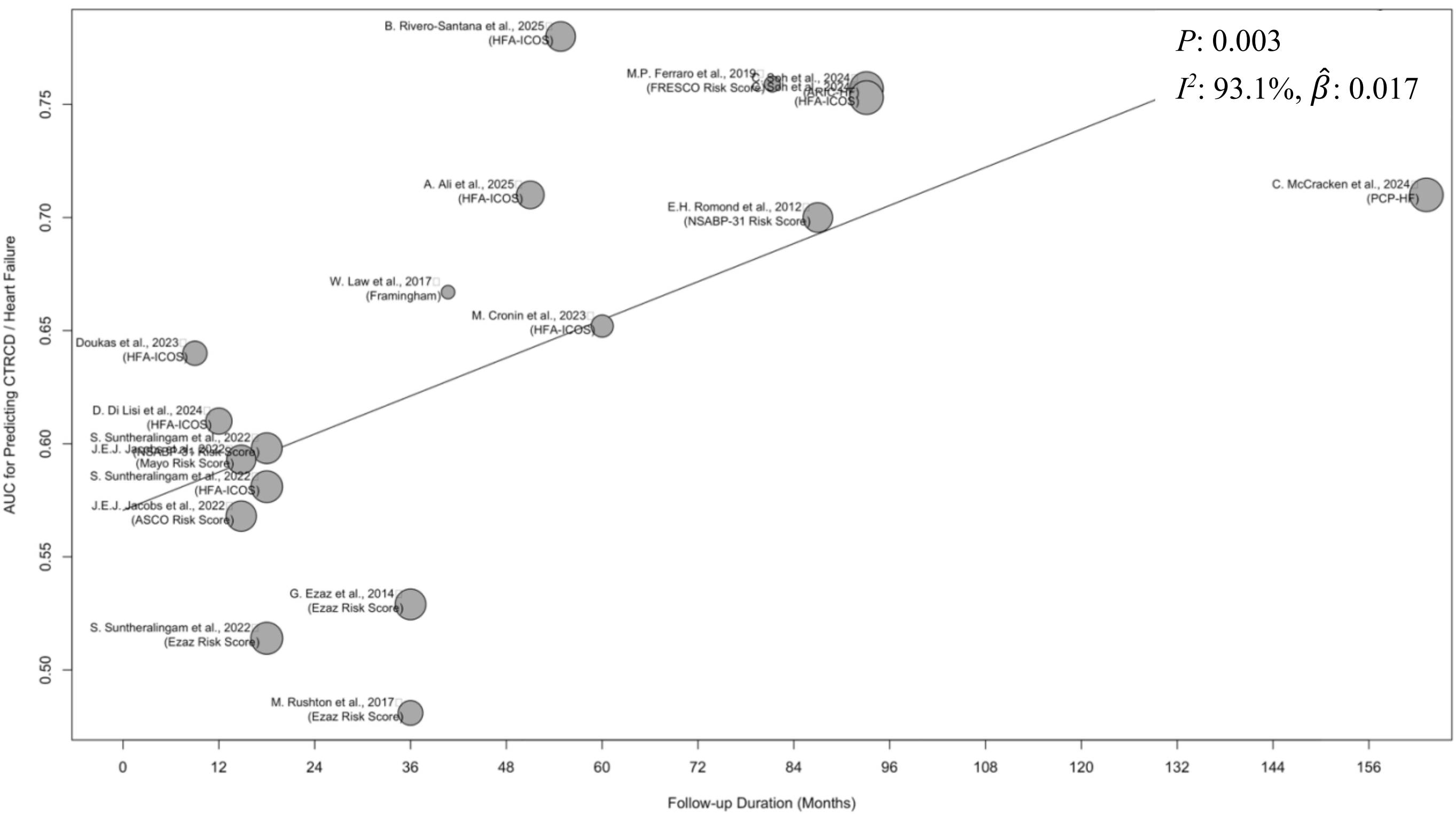

Each bubble represents a study; bubble size is proportional to study weight in the meta-regression. The solid line depicts the fitted regression line.

Meta-regression estimated regression coefficient ($\hat{\beta}$) represents the expected change in the AUC of cardiovascular risk scores for predicting CTRCD/heart failure associated with each additional year of median follow-up duration. I^2 represents residual heterogeneity between-study variation after accounting for the moderator in the meta-regression model.

Supplementary Figure 7. Bubble plot of the meta-regression assessing the association between median follow-up duration (months) and discriminative ability (AUC) of cardiovascular risk scores for predicting arrhythmia

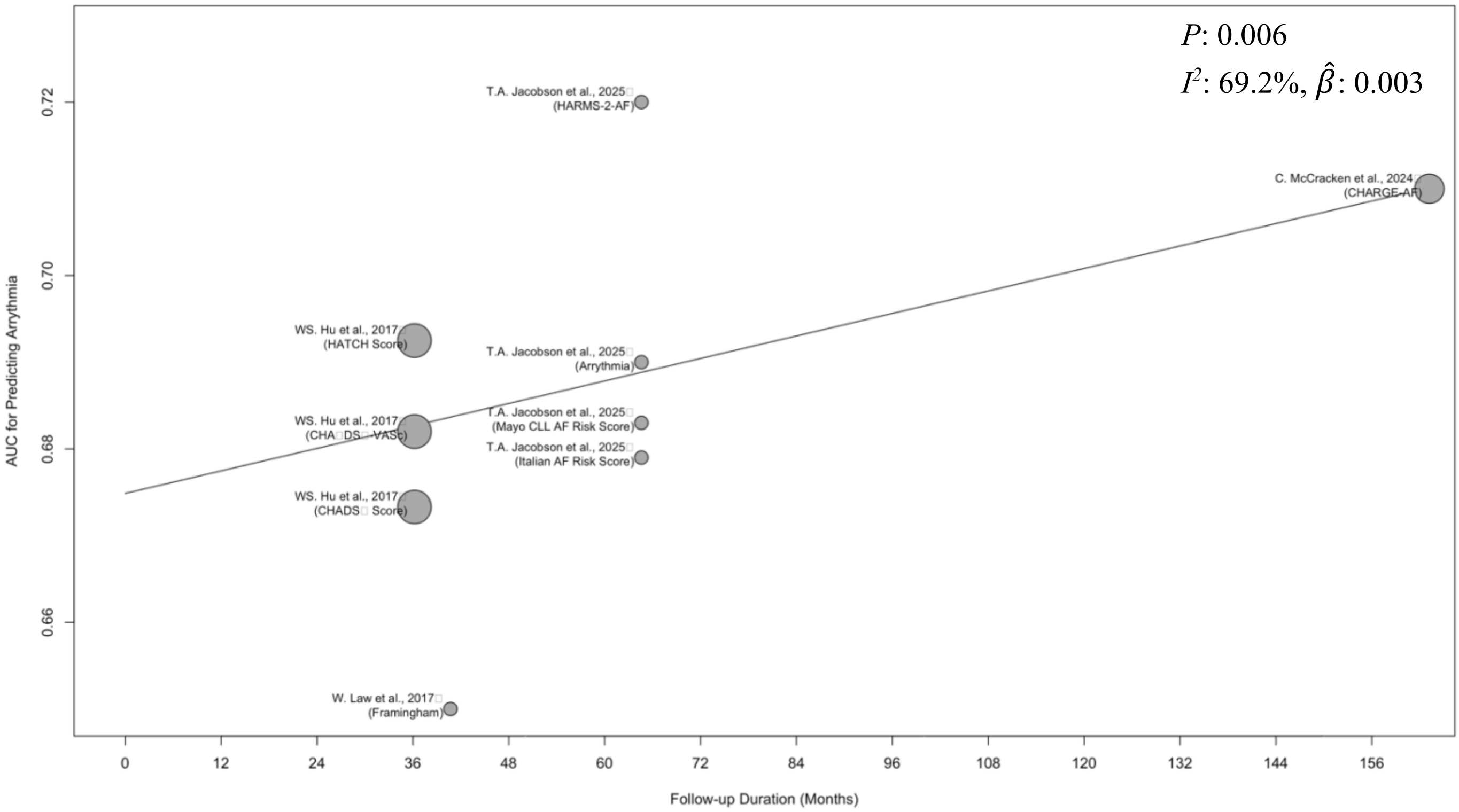

Each bubble represents a study; bubble size is proportional to study weight in the meta-regression. The solid line depicts the fitted regression line.

Meta-regression estimated regression coefficient ($\hat{\beta}$) represents the expected change in the AUC of cardiovascular risk scores for predicting arrhythmia associated with each additional year of median follow-up duration. I^2 represents residual heterogeneity between-study variation after accounting for the moderator in the meta-regression model.

Supplementary Figure 8. Bubble plot of the meta-regression assessing the association between median follow-up duration (months) and discriminative ability (AUC) of cardiovascular risk scores for predicting composite cardiovascular outcome

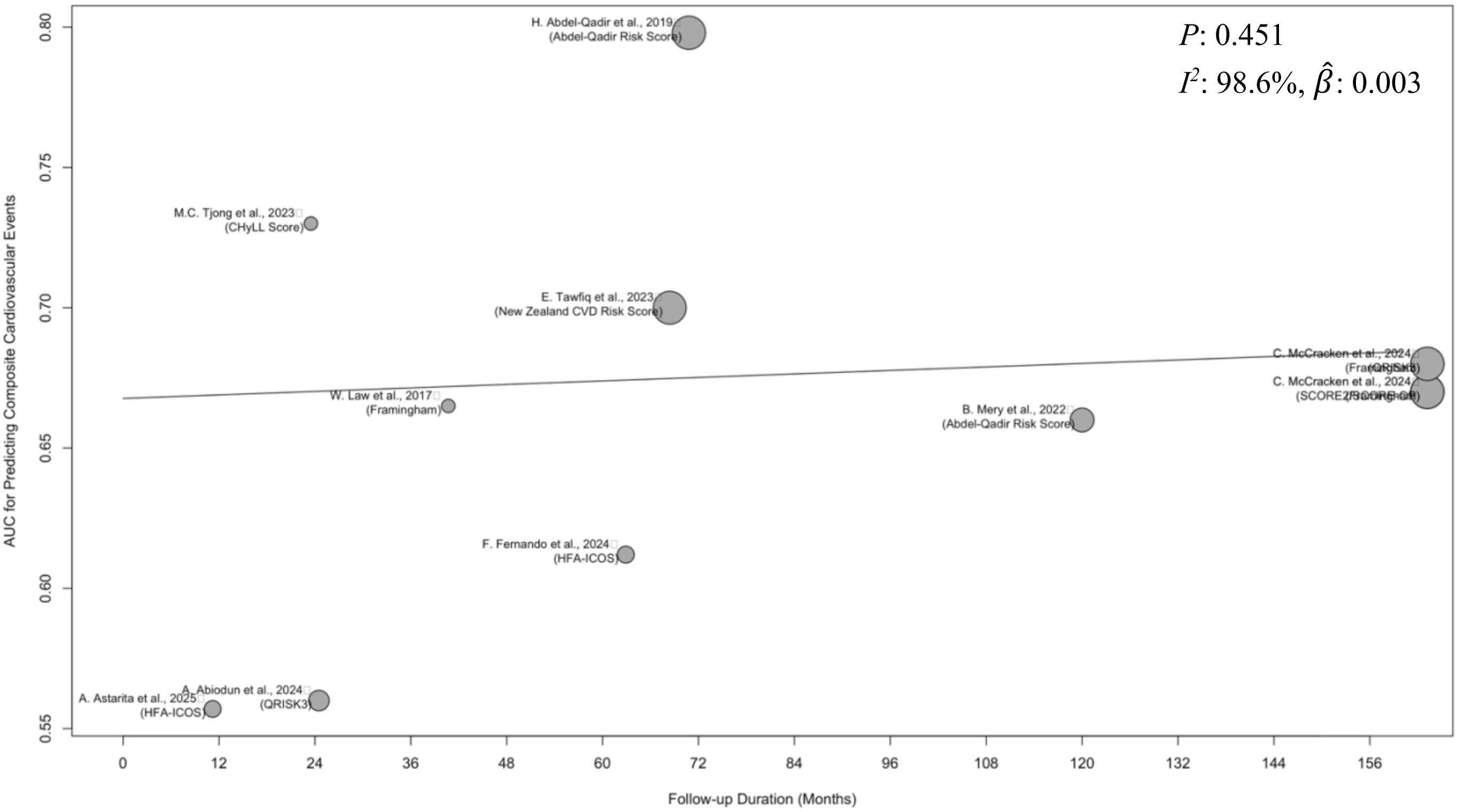

Each bubble represents a study; bubble size is proportional to study weight in the meta-regression. The solid line depicts the fitted regression line.

Meta-regression estimated regression coefficient ($\hat{\beta}$) represents the expected change in the AUC of cardiovascular risk scores for predicting composite cardiovascular outcome associated with each additional year of median follow-up duration. I^2 represents residual heterogeneity between-study variation after accounting for the moderator in the meta-regression model.

Supplementary Figure 9. Bubble plot of the meta-regression assessing the association between cohort population mean age (years) and discriminative ability (AUC) of cardiovascular risk scores for predicting vascular events

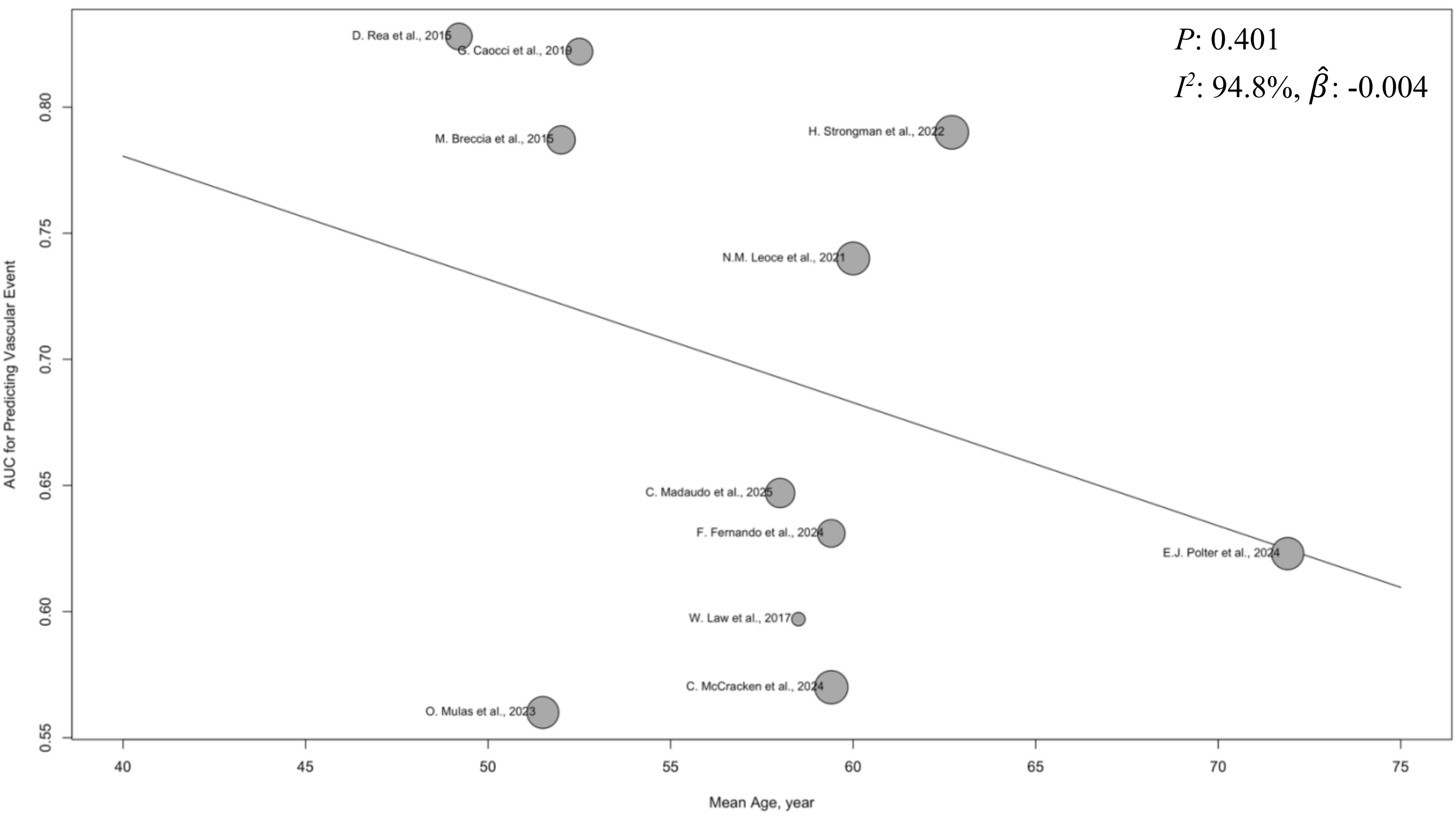

Each bubble represents a study; bubble size is proportional to study weight in the meta-regression. The solid line depicts the fitted regression line.

Meta-regression estimated regression coefficient ($\hat{\beta}$) represents the expected change in the AUC of cardiovascular risk scores for predicting vascular events associated with each one-year increase in study population mean age. I^2 represents residual heterogeneity between-study variation after accounting for the moderator in the meta-regression model.

Supplementary Figure 10. Bubble plot of the meta-regression assessing the association between cohort population mean age (years) and discriminative ability (AUC) of cardiovascular risk scores for predicting CTRCD/heart failure

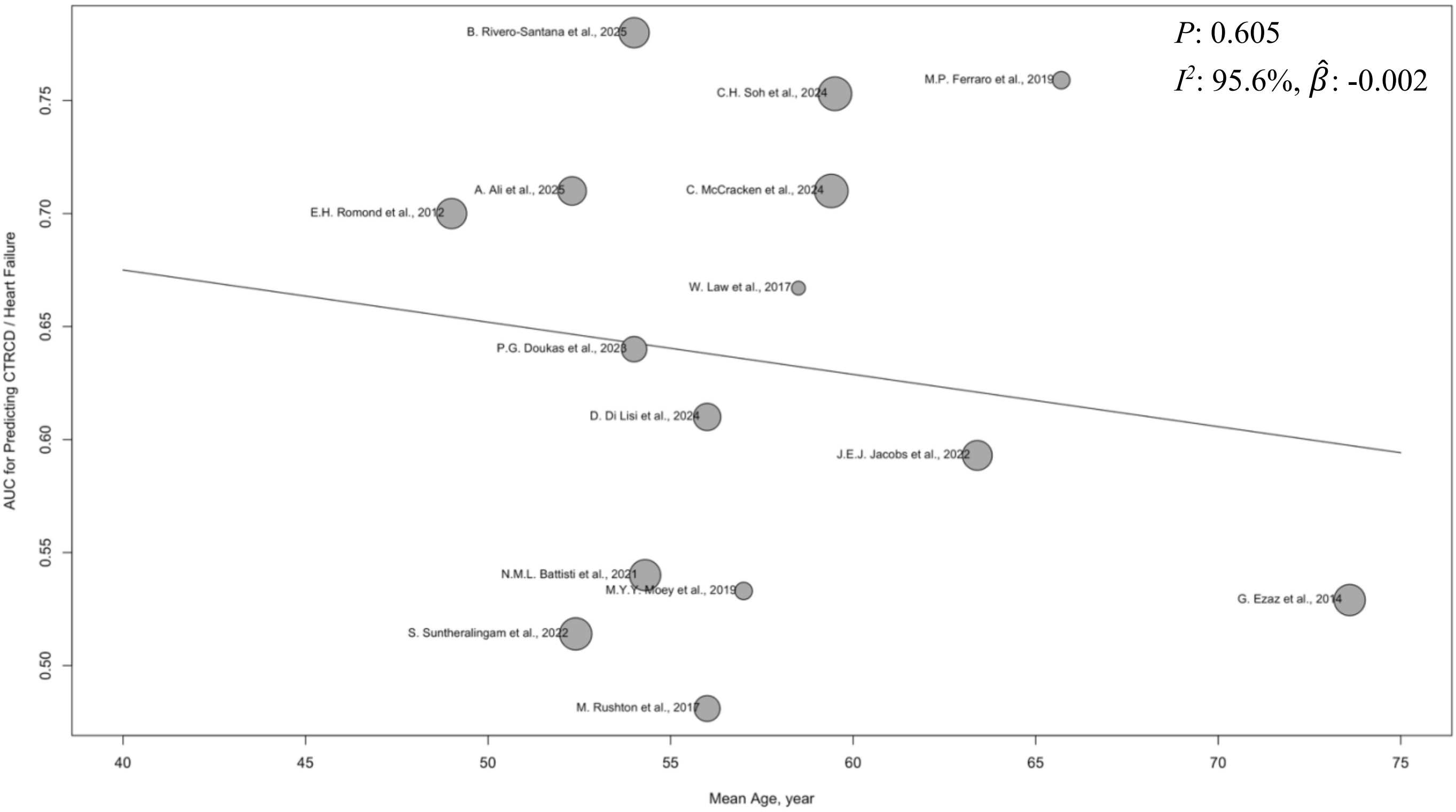

Each bubble represents a study; bubble size is proportional to study weight in the meta-regression. The solid line depicts the fitted regression line.

Meta-regression estimated regression coefficient ($\hat{\beta}$) represents the expected change in the AUC of cardiovascular risk scores for predicting CTRCD/heart failure associated with each one-year increase in study population mean age. I^2 represents residual heterogeneity between-study variation after accounting for the moderator in the meta-regression model.

Supplementary Figure 11. Bubble plot of the meta-regression assessing the association between cohort population mean age (years) and discriminative ability (AUC) of cardiovascular risk scores for predicting arrhythmia

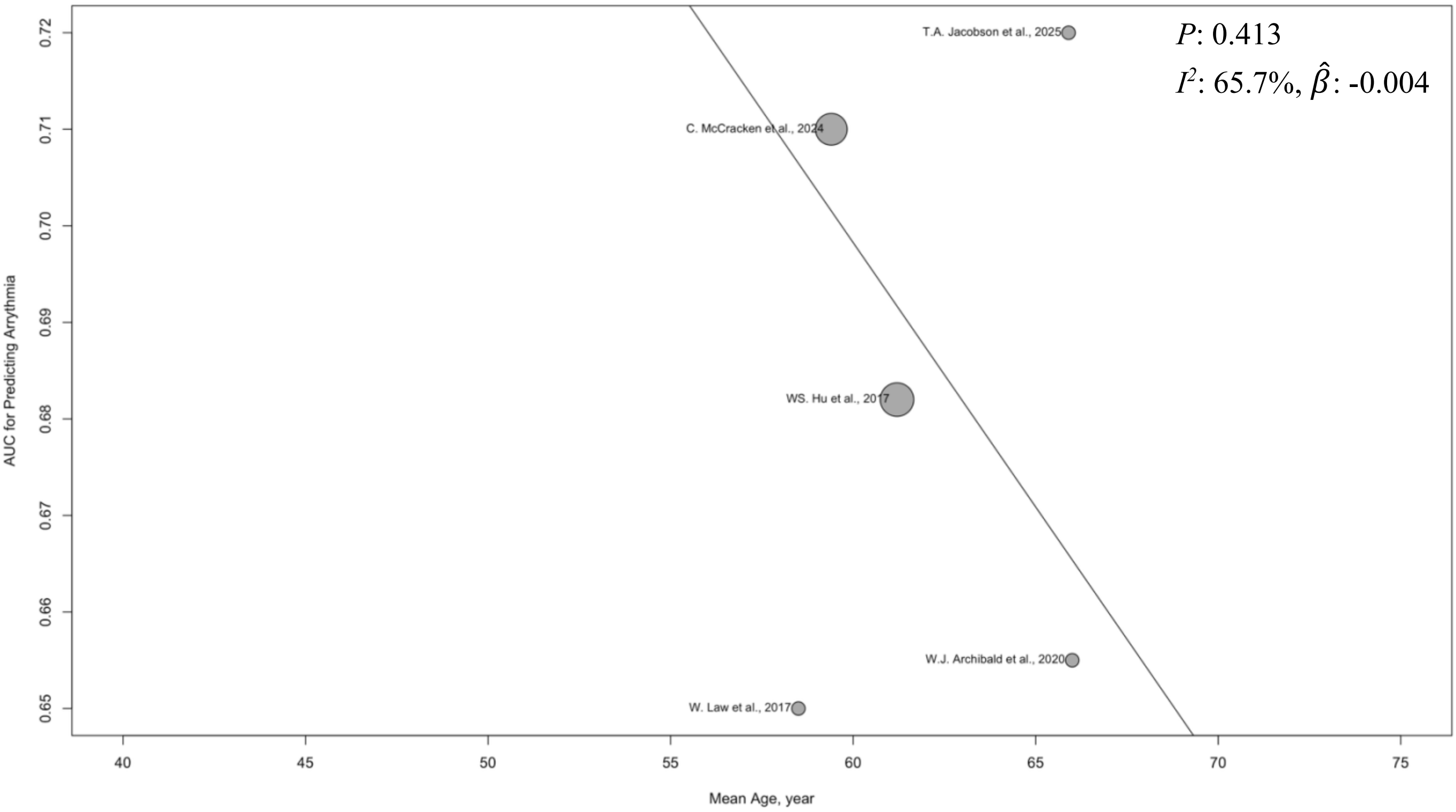

Each bubble represents a study; bubble size is proportional to study weight in the meta-regression. The solid line depicts the fitted regression line.

Meta-regression estimated regression coefficient ($\hat{\beta}$) represents the expected change in the AUC of cardiovascular risk scores for predicting arrhythmia associated with each one-year increase in study population mean age. I^2 represents residual heterogeneity between-study variation after accounting for the moderator in the meta-regression model.

Supplementary Figure 12. Bubble plot of the meta-regression assessing the association between cohort population mean age (years) and discriminative ability (AUC) of cardiovascular risk scores for predicting composite cardiovascular outcome

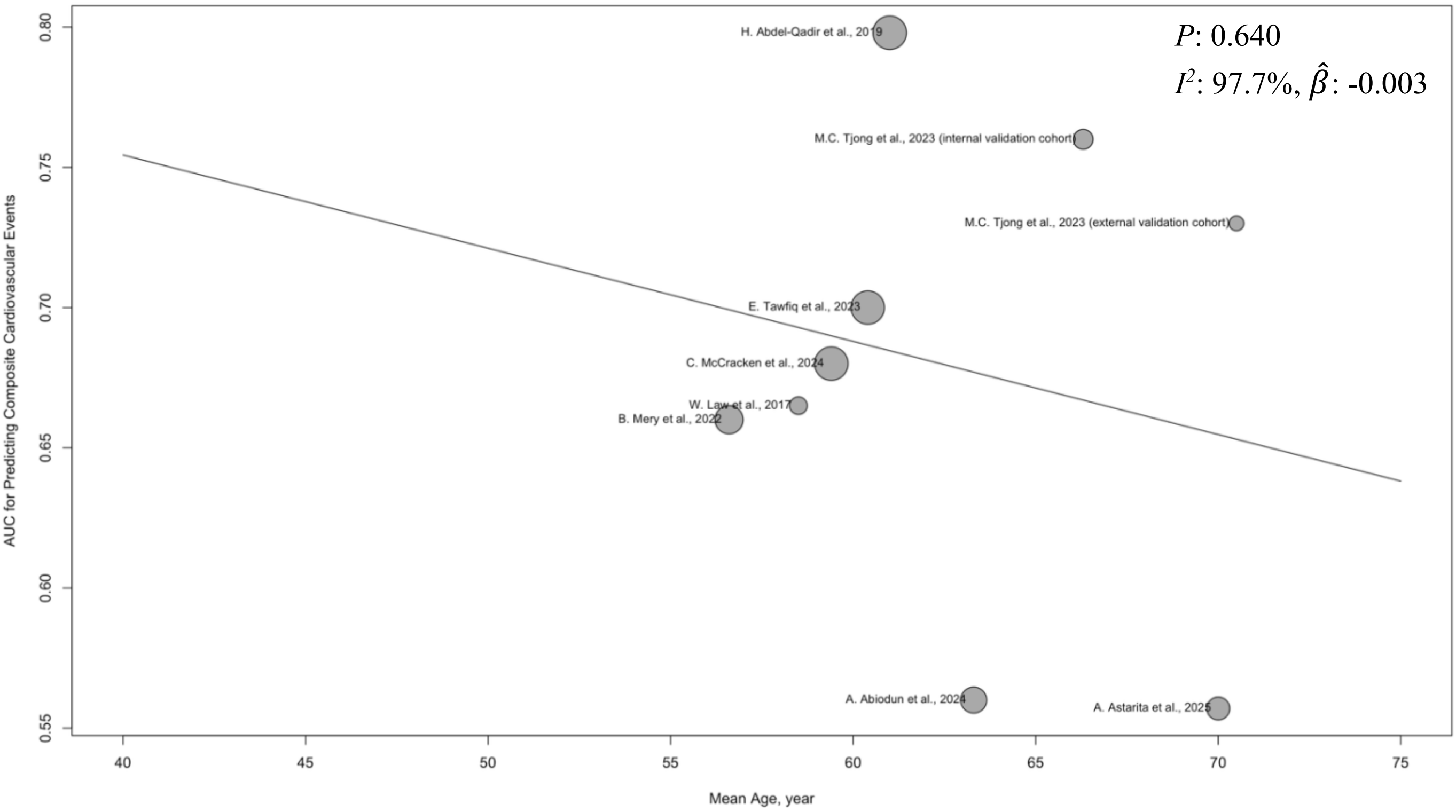

Each bubble represents a study; bubble size is proportional to study weight in the meta-regression. The solid line depicts the fitted regression line.

Meta-regression estimated regression coefficient ($\hat{\beta}$) represents the expected change in the AUC of cardiovascular risk scores for predicting composite cardiovascular outcome associated with each one-year increase in study population mean age. I^2 represents residual heterogeneity between-study variation after accounting for the moderator in the meta-regression model.

Quality Assessment

PROBAST Quality Assessment Results for Risk of Bias and Applicability

The risk of bias and applicability of the included studies were evaluated using the PROBAST tool. In the Participants domain, 10 studies (29.4%) were rated as high risk of bias, mostly due to retrospective designs or inclusion of very selective cohorts that limit generalizability, while 24 studies (70.6%) were rated low risk; no studies were rated unclear for this domain. In the Predictors domain, all 34 studies (100%) were rated as low risk of bias, with no studies rated high or unclear. In the Outcomes domain, 11 studies (32.3%) were rated as high risk, 3 studies (8.8%) as unclear risk, primarily due to insufficient detail on outcome definitions or timing, and 20 studies (58.8%) as low risk. In the Analysis domain, 17 studies (50.0%) were judged as high risk, largely due to inadequate sample size and low (<10 events-per-variable ratios), whereas 17 studies (50.0%) were rated low risk.

Applicability concerns followed a similar pattern. In the Participants domain, 9 studies (26.5%) had high applicability concerns, 1 (2.9%) was unclear, and 24 (70.6%) were low. High concern most often reflected study populations that did not fully represent the intended target population, potentially limiting generalizability. In the Predictors domain, all studies (100%) were of low concern, indicating that predictors were generally available, clearly defined, and measured in a manner applicable to clinical practice. In the Outcomes domain, 13 studies (38.2%) were of high concern, 3 (8.8%) unclear, and 18 (52.9%) low concern, primarily due to differences in outcome definitions or measurement protocols compared with recommendations for clinical implementation.

Supplementary Figure 13. Methodological quality summary of the included studies based on PROBAST criteria

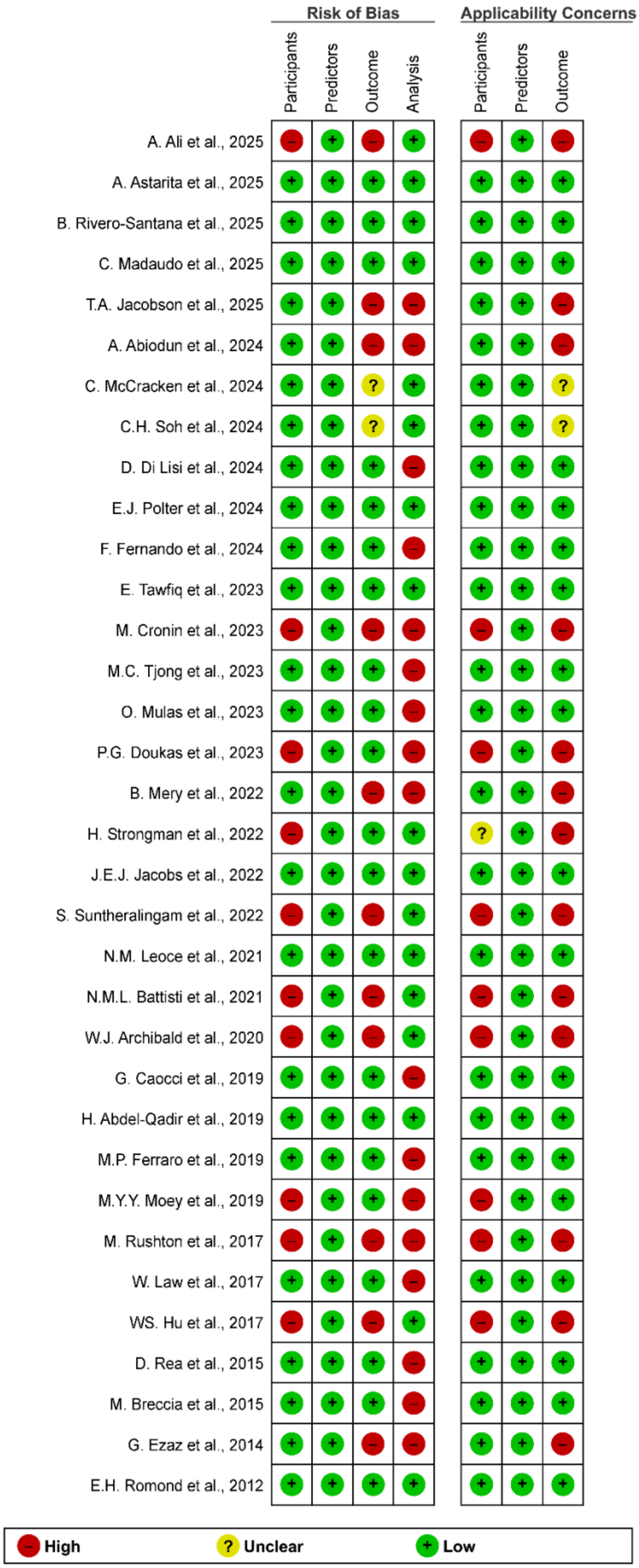

Supplementary Figure 14. Methodological quality graph of the included studies based on PROBAST criteria

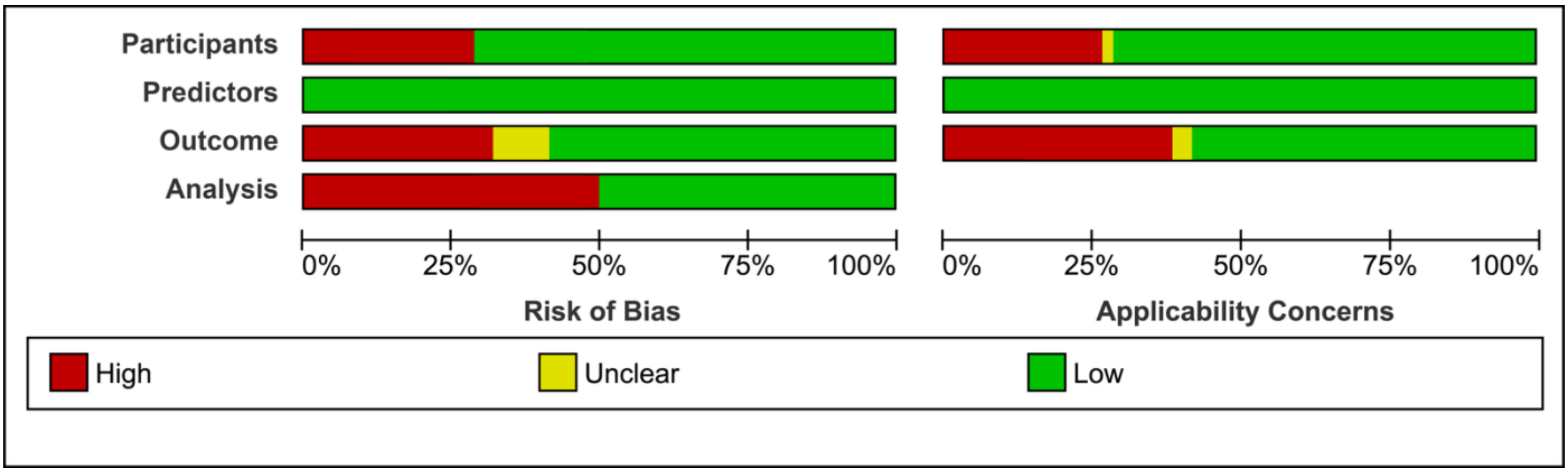

PRISMA 2020 Checklist

Section and Topic	Item #	Checklist item	Location where item is reported
**TITLE**			
Title	1	Identify the report as a systematic review.	Page 1, lines 1-2
**ABSTRACT**			
Abstract	2	See the PRISMA 2020 for Abstracts checklist.	Text Abstract
**INTRODUCTION**			
Rationale	3	Describe the rationale for the review in the context of existing knowledge.	Page 2, lines 21-39
Objectives	4	Provide an explicit statement of the objective(s) or question(s) the review addresses.	Page 2, line 40 to Page 3, line 47
**METHODS**			
Eligibility criteria	5	Specify the inclusion and exclusion criteria for the review and how studies were grouped for the syntheses.	Page 3, line 63 to Page 4, line 68 & Supplementary Material
Information sources	6	Specify all databases, registers, websites, organisations, reference lists and other sources searched or consulted to identify studies. Specify the date when each source was last searched or consulted.	Page 3, lines 57-62 & Supplementary Material
Search strategy	7	Present the full search strategies for all databases, registers and websites, including any filters and limits used.	Supplementary Material
Selection process	8	Specify the methods used to decide whether a study met the inclusion criteria of the review, including how many reviewers screened each record and each report retrieved, whether they worked independently, and if applicable, details of automation tools used in the process.	Page 4, lines 70-73 & Supplementary Material
Data collection process	9	Specify the methods used to collect data from reports, including how many reviewers collected data from each report, whether they worked independently, any processes for obtaining or confirming data from study investigators, and if applicable, details of automation tools used in the process.	Supplementary Material
Data items	10a	List and define all outcomes for which data were sought. Specify whether all results that were compatible with each outcome domain in each study were sought (e.g. for all measures, time points, analyses), and if not, the methods used to decide which results to collect.	Page 4, lines 74-82
	10b	List and define all other variables for which data were sought (e.g. participant and intervention characteristics, funding sources). Describe any assumptions made about any missing or unclear information.	Supplementary Material
Study risk of bias assessment	11	Specify the methods used to assess risk of bias in the included studies, including details of the tool(s) used, how many reviewers assessed each study and whether they worked independently, and if applicable, details of automation tools used in the process.	Page 4, line 84 to Page 5, line 90
Effect measures	12	Specify for each outcome the effect measure(s) (e.g. risk ratio, mean difference) used in the synthesis or presentation of results.	Page 5, line 92 to Page 6, line 114
Synthesis methods	13a	Describe the processes used to decide which studies were eligible for each synthesis (e.g. tabulating the study intervention characteristics and comparing against the planned groups for each synthesis (item #5)).	Page 3, line 63 to Page 4, line 68 & Supplementary Material
	13b	Describe any methods required to prepare the data for presentation or synthesis, such as handling of missing summary statistics, or data conversions.	Page 5, lines 100-105
	13c	Describe any methods used to tabulate or visually display results of individual studies and syntheses.	Page 5, lines 92-105
	13d	Describe any methods used to synthesize results and provide a rationale for the choice(s). If meta-analysis was performed, describe the model(s), method(s) to identify the presence and extent of statistical heterogeneity, and software package(s) used.	Page 5, lines 100-102 & Page 6, lines 113-114
	13e	Describe any methods used to explore possible causes of heterogeneity among study results (e.g. subgroup analysis, meta-regression).	Page 6, lines 115-125

Section and Topic	Item #	Checklist item	Location where item is reported
	13f	Describe any sensitivity analyses conducted to assess robustness of the synthesized results.	Page 6, lines 117-122
Reporting bias assessment	14	Describe any methods used to assess risk of bias due to missing results in a synthesis (arising from reporting biases).	N/A
Certainty assessment	15	Describe any methods used to assess certainty (or confidence) in the body of evidence for an outcome.	N/A
**RESULTS**			
Study selection	16a	Describe the results of the search and selection process, from the number of records identified in the search to the number of studies included in the review, ideally using a flow diagram.	Page 6, line 130 to Page 7, line 133 & Figure 1
	16b	Cite studies that might appear to meet the inclusion criteria, but which were excluded, and explain why they were excluded.	Page 7, line 133
Study characteristics	17	Cite each included study and present its characteristics.	Page 7, lines 133-151 & Table 1 & Supplementary Tables 1-2
Risk of bias in studies	18	Present assessments of risk of bias for each included study.	Page 13, lines 267-273
Results of individual studies	19	For all outcomes, present, for each study: (a) summary statistics for each group (where appropriate) and (b) an effect estimate and its precision (e.g. confidence/credible interval), ideally using structured tables or plots.	Supplementary Tables 1-4
Results of syntheses	20a	For each synthesis, briefly summarise the characteristics and risk of bias among contributing studies.	Page 8, line 8 to Page 11, line 233
	20b	Present results of all statistical syntheses conducted. If meta-analysis was done, present for each the summary estimate and its precision (e.g. confidence/credible interval) and measures of statistical heterogeneity. If comparing groups, describe the direction of the effect.	Page 8, line 153 to Page 11, line 4
	20c	Present results of all investigations of possible causes of heterogeneity among study results.	Page 9, line 176 to Page 13, line 265
	20d	Present results of all sensitivity analyses conducted to assess the robustness of the synthesized results.	Page 11, line 235 to Page 12, line254
Reporting biases	21	Present assessments of risk of bias due to missing results (arising from reporting biases) for each synthesis assessed.	N/A
Certainty of evidence	22	Present assessments of certainty (or confidence) in the body of evidence for each outcome assessed.	N/A
**DISCUSSION**			
Discussion	23a	Provide a general interpretation of the results in the context of other evidence.	Page 14, line 289 to Page 16, line 335
	23b	Discuss any limitations of the evidence included in the review.	Page 17, line 369 to Page 18, line 396
	23c	Discuss any limitations of the review processes used.	Page 17, line 369 to Page 18, line 396
	23d	Discuss implications of the results for practice, policy, and future research.	Page 16, line 337 to Page 17, line 367
**OTHER INFORMATION**			
Registration and protocol	24a	Provide registration information for the review, including register name and registration number, or state that the review was not registered.	Page 3, lines 53-55 & Reference #16
	24b	Indicate where the review protocol can be accessed, or state that a protocol was not prepared.	Page 3, lines 53-55 & Reference #16
	24c	Describe and explain any amendments to information provided at registration or in the protocol.	N/A
Support	25	Describe sources of financial or non-financial support for the review, and the role of the funders or sponsors in the review.	EHJ Declarations Form

Section and Topic	Item #	Checklist item	Location where item is reported
Competing interests	26	Declare any competing interests of review authors.	EHJ Declarations Form
Availability of data, code and other materials	27	Report which of the following are publicly available and where they can be found: template data collection forms; data extracted from included studies; data used for all analyses; analytic code; any other materials used in the review.	EHJ Declarations Form
